# Elevated SLC40A1 impairs cardiac function and exacerbates mitochondrial dysfunction, oxidative stress, and apoptosis in ischemic myocardia

**DOI:** 10.7150/ijbs.89368

**Published:** 2024-01-01

**Authors:** Renqian Feng, Di Wang, Tiantian Li, Xulin Liu, Tingwei Peng, Mingchuan Liu, Gaotong Ren, Haowei Xu, Haixia Luo, Denghui Lu, Bingchao Qi, Mingming Zhang, Yan Li

**Affiliations:** 1 Department of Cardiology, Tangdu Hospital, Air Force Medical University, Xi'an, 710032, China.; 2 Department of Orthodontics, Stomatology Hospital, Air Force Medical University, Xi'an, 710032, China.; 3 Department of Cardiology, NO. 988 Hospital of Joint Logistic Sopport Force, Zhengzhou, 450007, China.

**Keywords:** Iron, SLC40A1, Steap4, Heart failure, Myocardial infarction

## Abstract

Iron homeostasis is crucial for optimal cardiac function. Iron deficiency and overload have been linked to the development of cardiomyopathy and heart failure (HF) via intricate mechanisms. Although the crucial role of SLC40A1 in iron metabolism by facilitating the efflux of cellular iron has been confirmed, its specific molecular functions in cardiovascular diseases remain poorly understood. In this study, we generated mice with inducible cardiomyocyte-specific overexpression of SLC40A1 for the first time. The overexpression of SLC40A1 in the cardiomyocytes of adult mice resulted in significant iron deficiency, leading to mitochondrial dysfunction, oxidative stress, and apoptosis, subsequently resulting in the development of fatal HF. Notably, SLC40A1 upregulation was observed in the ischemic region during the initial phase of myocardial infarction (MI), contributing to iron loss in the cardiomyocytes. Conversely, the cardiomyocyte-specific knockdown of SLC40A1 improved cardiac dysfunction after MI by enhancing mitochondrial function, suppressing oxidative stress, and reducing cardiomyocytes apoptosis. Mechanistically, Steap4 interacted with SLC40A1, facilitating SLC40A1-mediated iron efflux from cardiomyocytes. In short, our study presents evidence for the involvement of SLC40A1 in the regulation of myocardial iron levels and the therapeutic benefits of cardiomyocyte-specific knockdown of SLC40A1 in MI in mice.

## Introduction

Heart failure (HF) is a significant global public health concern that profoundly impacts an individual's well-being due to its high prevalence, mortality rates, and hospitalization rates 1. Iron plays a crucial role in supporting the high-energy requirements of cardiomyocytes because of its capacity to transport electrons effectively and facilitate various vital biochemical reactions 2. Systemic iron deficiency (ID) is prevalent in approximately 50% of the patients diagnosed with HF. Moreover, many studies have provided evidence that ID has a detrimental impact on the clinical and prognostic outcomes of patients with HF 3-5. Direct analysis of the heart tissue from patients with HF has revealed a frequent occurrence of myocardial iron deficits 6. According to the HF guidelines, ferric carboxymaltose is suggested to improve exercise capacity and enhance the quality of life in patients with HF 7. However, it is worth noting that the effectiveness of systematic iron supplementation in increasing myocardial iron content is limited 8, 9.

Myocardial infarction (MI) is the leading cause of HF 10. The primary pathological factors contributing to HF after MI are cardiomyocyte death and dysfunction of the remaining viable cardiomyocytes 11. Elevated myocardial iron content frequently occurs after reperfusion, particularly when an ischemia-reperfusion (I/R) injury leads to a microvascular injury 12, 13. Nevertheless, a recent study indicated that the administration of iron chelator does not reduce the infarct size of I/R 14. Still, iron deficiency can exacerbate I/R injury 15, 16. Myocardial ischemia and I/R are distinct pathological phenomena. However, the role of iron in ischemic cardiomyocytes remains unclear. Consequently, the issue of effectively balancing iron levels in cardiomyocytes following MI warrants further examination and discussion.

SLC40A1, also known as FPN1, MTP1, or IREG1, plays a pivotal role in regulating iron homeostasis and is currently the only identified iron exporter in mammals 17. The transmembrane protein SLC40A1 is primarily localized to the cell membrane and endosomes 18. It functions as an exporter of iron in the form of Fe^2+^^19^, which plays a crucial role in facilitating the transport of absorbed, stored, and recycled iron from duodenal cells, hepatocytes, and macrophages to plasma 17. The transcriptional regulation of SLC40A1 is influenced by various factors, including hypoxia, iron levels, and inflammatory signals 20. However, its role in MI remains unclear.

In this study, we investigated the physiological function of SLC40A1 in normal myocardia, as well as its pathological implications in MI, and elucidated the underlying molecular mechanisms involved.

## Materials and Methods

### Animals

The animal experiments adhered to the protocols outlined in the Guide for the Care and Use of Laboratory Animals and received approval from the Animal Care Committee of the Air Force Medical University. Shanghai Model Organisms Center, Inc. provided *Slc40a1* transgenic mice (C57BL/6J background), whereas the animal center at the Air Force Medical University provided additional experimental mice (with a C57BL/6 J background). Each mouse was housed under controlled conditions, with a temperature maintained at 21 ± 0.5°C, humidity at 60 ± 5%, and a 12-hour light-dark cycle. The mice were given ad libitum access to food and water. Random assignment of the animals was conducted, and the experimenters remained blind to the histology assays, *in vivo* function tests, and outcome evaluation.

### Genomic screening

The Genomic DNA Kit (Tiangen, China) was used to extract genomic DNA from the tail of *Slc40a1* transgenic (TG) and *Slc40a1* non-transgenic (NTG) mice, following the instructions provided by the manufacturer. The TG mice utilized in this research comprised of double-heterozygotes for Rosa26-LSL (LoxP-Stop-LoxP)-*Slc40a1* and Myh6-Cre genotypes. The genotype of NTG group was either Rosa26-LSL-*Slc40a1* or Myh6-Cre heterozygote. Upon reaching the age of 8-10 weeks, the TG mice exhibited the initiation of exogenous *Slc40a1* expression in their hearts subsequent to consumption of tamoxifen (0.1875‰, Sigma-Aldrich, USA)-containing chow. The diet administered to NTG mice remained consistent with that of the TG mice.

### Model of mouse MI

To induce MI, male mice aged 8-9 weeks were anesthetized with isoflurane (1-2%) using the same procedure as previously described 21. Briefly, the mouse hearts were promptly extracted from the thoracic cavity by squeezing through the left thoracic incision. Next, the left anterior descending (LAD) coronary artery was ligated on the left side using a silk thread (6-0). The effectiveness of the procedure could be determined by observing alterations in the electrocardiogram and the appearance of a pale region in the ischemic area. During the same surgical procedure, sham-operated control mice had their coronary arteries no ligated.

### Cell isolation and culture

Neonatal rat/mouse cardiomyocytes (NRCMs/NMCMs) were obtained from Sprague-Dawley rats/C57BL/6J mice that were 1-2 days old. The cardiac tissue was rinsed with PBS three times to eliminate blood. Following fragmentation, the specimen underwent digestion using a solution of type Ⅱ collagenase (0.8 mg/ml, Thermo Fisher Scientific, USA) for a total of 6 to 7 cycles. Finally, an additional amount of medium was included to ensure the completion of the digestion procedure. To remove as many fibroblasts as possible, a differential attachment approach was used. During the first 48 hours, the primary cardiomyocytes were cultured in a full medium. HEK-293T cells were exclusively used for FRET assays. For hypoxic conditions, cells were incubated in the Forma Steri-Cult (Thermo Fisher Scientific, USA) with 1% oxygen, 94% nitrogen, and 5% carbon dioxide.

### Real-time quantitative polymerase chain reaction (qPCR)

The RNAiso Plus (Takara, Japan) was utilized to extract total RNA from heart tissues for qPCR. Reverse transcription was performed using the PrimeScript RT Reagent Kit with gDNA Eraser (Takara, Japan). The SYBR Premix Ex Ta II (Takara, Japan) was employed for qPCR following the manufacturer's guidelines. The primer sequences can be found in Table S1. The cycling temperature for 40 cycles was adjusted to 95°C for 15 seconds, followed by 55°C for 15 seconds, and then 72°C for 15 seconds. β-Actin was utilized as an internal reference for control purposes.

### Western blotting

To homogenize and lyse left ventricle tissues (below the ligature in the MI group) and cultured cardiomyocytes, the RIPA buffer from Beyotime in China was utilized. After separating proteins (20 μg) using SDS-PAGE gels, they were then transferred onto polyvinylidene fluoride (PVDF) membranes. These membranes were blocked with a 5% milk solution and underwent overnight incubation at a temperature of 4 °C with primary antibodies. Table S2 contains detail information of all antibodies. Detection of the bands was performed using a chemiluminescence system (Bio-Rad, USA) after 1 h of incubation at 37°C with HRP-conjugated secondary antibodies. The LabImage software (Bio-Rad, USA) was used to analyze and measure the intensity of immunoblot band. As an internal control, we utilized β-Actin.

### Staining in histology

After being fixed overnight with 4% paraformaldehyde, the mouse heart slices underwent staining in histology. Subsequently, they were embedded in paraffin and sliced into pieces measuring 5-μm. The cardiac collagen content was estimated using Masson's trichrome staining. The size of the infarct is determined by dividing the total diameter of the infarct by the circumference of the left ventricle. Mouse hearts were collected for triphenyl tetrazolium chloride (TTC) staining approximately 8 hours after MI, followed by the continuous cutting of four 1.0 mm thick sections. After incubating the slices with a 1% TTC solution (Solarbio, China), a photograph was captured. The images were analyzed using the ImageJ software.

### Echocardiography

Transthoracic echocardiography in M-mode was performed using the VINNO 6 ultrasound machine manufactured by VINNO, Inc. in China. Before the trial, mouse chest hairs were eliminated with depilatory lotion. The mice were anesthetized using 2% isoflurane and then placed on a heating pad. All mouse hearts were imaged in long-axis views in M-mode, with heart rates ranging from 450 to 600 beats per minute. VINNO 6 software (VINNO, China) was used to indirectly measure the left ventricle (LV) end-diastolic internal diameter, LV end-systolic internal diameter, LV posterior wall thickness at diastole ends and systole ends, and LV anterior wall thickness at diastole ends and systole ends. Additional analysis yielded the acquisition of the ejection fraction and fractional shortening. Researchers, who had no knowledge of the experimental treatments, performed all the measurements.

### Construction and administration of AAV9 vectors

The animal model utilized Adeno-associated virus serotype 9 (AAV9) to suppress the target gene, which was regulated by a promoter called cardiac troponin T (cTnT). GeneChem Technology (China) and Hanbio Biotechnology (China) constructed AAV9-cTnT-sh*Slc40a1* and AAV9-cTnT-sh*Steap4*, along with their corresponding control AAV9-cTnT-Ctrl, respectively. Following the administration of 2% isoflurane anesthesia, intrathoracic injection of 10^11^ viral particles was performed on the mice at the age of 4 weeks. The control group received AAV9-Ctrl using the identical procedure. Table S3 contains the shRNA target sequences.

### Target gene expression upregulation and downregulation in cardiomyocytes

Ad-*Slc40a1* and Ad-Ctrl constructs were generated by Hanbio Biotechnology (China) using adenoviral vectors carrying *Slc40a1*. Ad-sh*Slc40a1* and Ad-shCtrl were provided by GeneChem Technology (Shanghai, China), in the form of adenoviral vectors carrying shRNA-*Slc40a1*.Protein expression was observed 48 hours after viral infection. The plasmid containing Steap4 and the negative control plasmid (OE-NC) were created and produced by Tsingke Biotech (China). Cardiomyocytes were transfected with the plasmid using Lipofectamine 3000 from Invitrogen (USA). Protein expression was measured 72 hours after transfection. The shRNA target sequences are shown in Table S3.

### Immunohistochemistry and immunofluorescence analysis

As mentioned earlier 22, immunohistochemical staining was conducted on cardiac sections, along with immunofluorescence analysis of primary neonatal cardiomyocytes. The images were analyzed using the ImageJ software. The antibodies employed and their respective dilution rates are documented in Table S2

### Transmission electron microscopy (TEM)

TEM was employed to examine the structure of myocardial mitochondrial cristae, and the method for preparing heart samples followed the same protocol as described previously 22. The left ventricles of mice were sliced into 1-2 mm wide longitudinal strips along fiber orientation. After 24 hours of glutaraldehyde prefixation, postfixation was carried out at 4 °C for 2 hours using a 1% solution of osmium tetroxide. Following the process of dehydration and embedding, the specimens were sliced into extremely thin sections and then treated with uranium acetate and lead citrate for staining. The transmission electron microscope (JEM-1230; JEOL, Japan) was used to capture all the images.

### Mitochondrial respiratory function measurement

Measurement of respiratory function in mitochondria was conducted using a 24-well seahorse test plate (Agilent Seahorse Bioscience, USA). The plate was evenly filled with cardiomyocytes obtained from a recently born rat. Following a 2-day incubation, adenovirus was introduced for another 2 days before performing a mitochondrial oxygen consumption rate (OCR) test using an XF24 Extracellular Flux Analyzer (Agilent Seahorse Bioscience), as previously recorded. The concentrations used for the four reagents were as follows: 1 µM oligomycin, 1 µM FCCP, 1 µM rotenone, and 0.5 µM antimycin A. The software XF Cell Mito Stress Test Generator (Agilent Seahorse Bioscience) was employed for the collection and analysis of OCR data.

### TUNEL staining

Apoptosis in cardiac tissue was evaluated using the TUNEL assay kit (Sigma-Aldrich, USA). To determine the ratio of apoptotic nuclei, the total count of TUNEL-positive nuclei was divided by the total count of DAPI-positive nuclei. Each paraffin segment had six random fields evaluated.

### DHE staining

The levels of reactive oxygen species (ROS) in the entire heart muscle were evaluated by exposing the frozen sections to a solution of dihydroethidium (DHE) from Beyotime, China, for a duration of 30 minutes at a temperature of 37 °C. FV3000 confocal laser scanning microscope (Olympus, Japan) was used to capture all images, which were then processed using ImageJ software.

### Live Cell Imaging

FerroOrange (Dojindo, Japan) staining was used to assess the levels of free ferrous in neonatal mouse cardiomyocytes (NMCMs), in accordance with the instructions provided by the manufacturer. Afterwards, the confocal laser scanning microscope (FV3000; Olympus, Japan) was used to obtain all images, which were then analyzed with the ImageJ software.

### Fluorescence resonance energy transfer (FRET) measurements

Lipofectin was used to transfect SLC40A1-ECFP (donor) and Steap4-EYFP (acceptor) into 293T cells. Cells were observed using a confocal laser scanning microscope (FV3000; Olympus, Japan) quipped with an excitation filter of 405 nm and an emission filter of 430-470 nm to detect ECFP. Cells expressing EYFP were detected by using a 488 nm excitation filter and a 510-580 nm emission filter. For the purpose of FRET analysis, the filters employed for FRET consisted of a 405 nm excitation filter and an emission filter ranging from 515 to 525 nm. The FRET measurements were conducted using CellSens software (FV3000; Olympus, Japan), as previously reported 23. In summary, the normalized FRET was calculated using fluorescence signals from the donor, acceptor, and FRET channels after background subtraction.

### Co-immunoprecipitation (Co-IP)

Co-IP was conducted using the Pierce Classic Magnetic IP/Co-IP Kit (Thermo Fisher Scientific, USA) following the guidelines provided by the manufacturer. To summarize, fresh mouse hearts were homogenized and lysed to obtain cell lysates. These lysates were then subjected to immunoprecipitation using either Flag or Steap4 antibody, and the process was carried out overnight at a temperature of 4ºC. Protein A/G magnetic beads were added at room temperature and left to attach to the antigen/antibody complex for 1 hour. Following multiple washes to remove impurities, the antigen/antibody complex was eluted. Protein samples containing dithiothreitol were then subjected to heating and separated via electrophoresis. Proteins were immunoblotted with Flag or Steap4 antibody after being transferred to PVDF membranes to confirm the effectiveness of immunoprecipitation.

### LC-MS/MS analysis for Flag-interacting proteins

The LC-MS/MS analysis was performed at Novogene Genetics (China), utilizing a Q Exactive HF-X mass spectrometer (Thermo Fisher Scientific) with an EASY-nLC 1200 UHPLC system, as stated earlier 22.

### Isolation of mitochondria

In accordance with the manufacturer's instructions, mouse hearts were used for mitochondrial isolation using the Tissue Mitochondria Isolation Kit (Beyotime, China).

### Measurements of NADP^+^/NADPH, MDA and ATP content

The NADP^+^/NADPH levels in the heart were evaluated by utilizing the NADP^+^/NADPH Assay Kit (Beyotime, China), while the MDA levels in cardiac tissues were determined using the MDA Content Assay Kit (Solarbio, China). Additionally, the cardiac ATP content was computed by following the manufacturer's instructions provided with the ATP Assay Kit (Beyotime, China).

### Measurements of serum, NMCMs and cardiac tissue total iron content

Serum was obtained by centrifuging whole blood samples at a speed of 3000 rpm for a duration of 10 minutes. Serum iron and cardiac total iron were assessed using the Total Iron Colorimetric Assay Kit (Elabscience, China) as per the provided instructions. The NMCMs' iron levels were determined by employing the Cell Total Iron Colorimetric Assay Kit (Elabscience, China) in accordance with the instructions provided by the manufacturer.

### Analysis of public datasets

The RNA-seq data (GDS651/223044_at and GDS2206/IMAGp998I15113) for human heart tissues were acquired from the GEO Profiles (www.ncbi.nlm.nih.gov/geoprofiles). The GSE1145 dataset, a high-throughput sequencing dataset, was utilized to investigate heart failure resulting from various causes. Its RNA samples were extracted from 11 non-failure (NF) hearts, 15 idiopathic dilated cardiomyopathy (IDC) hearts, and 11 ischemic cardiomyopathy (ICM) hearts. Additionally, the GSE3586 dataset, another high-throughput sequencing dataset, was utilized to study dilated cardiomyopathy. Its RNA samples were extracted from 15 NF hearts and 13 dilated cardiomyopathy (DCM) hearts.

### RNA sequencing and data analysis

RNA Whole-genome gene expression analysis was performed at Novogene Genetics (China) on heart tissues obtained from TG and NTG mice (n=3 per group), following the previously described method 24. In summary, RNAiso Plus (Takara, Japan) was utilized for the extraction of total RNA, and cDNA sequences were obtained using the HiSeq3000 sequencing system (Illumina). The genetic material and gene data for Mus musculus were obtained from the National Center for Biotechnology Information (NCBI) database. Afterward, the differentially expressed genes underwent a heat map analysis and GO gene ontology enrichment analysis. To evaluate the significant enrichment of the gene sets, a P.adjust (Padj) threshold of less than 0.5 was employed for the GO enrichment analysis.

### Analysis of oxylipins

As mentioned earlier 24, oxylipins were evaluated using liquid chromatography-mass spectrometry (LC-MS) technique, specifically utilizing the highly sensitive SCIEX QTRAP 6500 + mass spectrometry platform, well-known for its outstanding sensitivity, at Novogene Genetics (China).

### Statistics analysis

The experimental data was analyzed using GraphPad Prism 9.0 software (GraphPad Software, USA). The mean ± SEM was used to present all values. Student's t test was employed to compare two groups, while one-way ANOVA was utilized to compare more than two groups, followed by post-hoc tests with minimal differences. The Gehan-Breslow-Wilcoxon test was used to evaluate survival data. Statistical significance was determined by setting a threshold of P < 0.05.

## Results

### Cardiomyocyte-specific overexpression of SLC40A1 resulted in the development of fatal HF in mice

**Figure 1:**
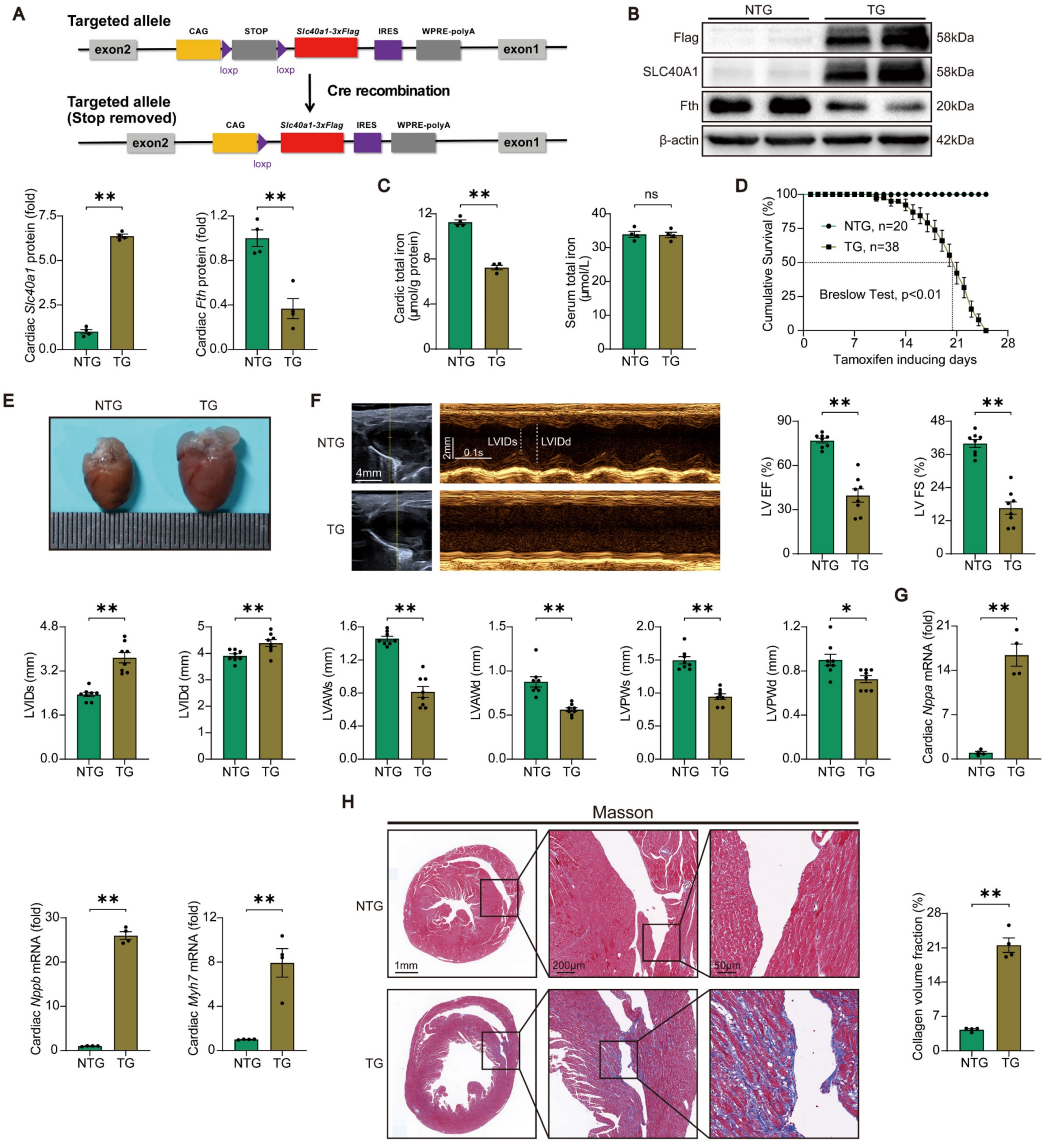
** TG mice developed fatal heart failure.** (A) A schematic diagram illustrating the methodology employed for the generation of Cre-inducible *Slc40a1* transgenic mice is presented. Upon Cre-mediated recombination, the *Slc40a1* allele is activated in specific cells. (B) Western blot analysis of cardiac *Slc40a1* and *Fth* proteins in NTG and TG mice (n = 4). (C) Concentrations of total iron in the heart and serum were quantified in NTG and TG mice (n = 4). (D) Cumulative survival rates of NTG (n = 20) and TG (n = 38) mice were recorded. (E) Representative images of the heart in both NTG and TG mice are displayed. (F) Echocardiograph analysis in long-axis M-mode (n = 8). The parameters measured included left ventricle (LV), ejection fraction (EF), fractional shortening (FS), LV end-systolic internal diameter (LVIDs), LV end-diastolic internal diameter (LVIDd), LV anterior wall thickness at the ends of systole (LVAWs), LV anterior wall thickness at the ends of diastole (LVAWd), LV posterior wall thickness at the ends of systole (LVPWs), and LV posterior wall thickness at the ends of diastole (LVPWd). (G) Expression levels of cardiac *Nppa*, *Nppb*, and *Myh7* mRNA were measured using qPCR (n = 4). (H) Masson trichrome staining images and quantification of fibrotic areas (n = 4). The data is presented as the mean value plus or minus the standard error of the mean. Statistical significance is denoted by *p < 0.05 and **p < 0.01.

Initially, we assessed the relative expression levels of SLC40A1 in various tissues. Our findings indicate that while SLC40A1 expression was not the most prominent in the myocardium, it exhibited the highest expression at 55kDa in this particular tissue (Figure S1A). Previous studies have reported SLC40A1 as a multimer 25, and our subsequent gene manipulation experiments confirmed the major form of myocardial SLC40A1 at 55kDa. To elucidate the role of SLC40A1 in the myocardium, we employed the Myh6-Cre-LSL (LoxP-Stop-LoxP) system to generate a transgenic mouse model with specific overexpression of SLC40A1 in the cardiomyocyte (Fig. 1A and Figure S2A). As anticipated, the overexpression of SLC40A1 resulted in a significant decrease in cardiac ferritin and total iron content, while the serum iron level did not exhibit a significant change (Fig. 1B-C). It is noteworthy that the TG mice had enlarged hearts, as evidenced by an increase in heart weight/body weight ratio, and experienced a rapid decline in survival, with a median survival rate of less than 3 weeks (Fig. 1D-E and Figure S3A). Subsequently, we conducted echocardiography on both NTG and TG mice fed with a tamoxifen-containing diet for 18 days (due to a rapid increase in the number of new deaths per day for more than 18 days). The results showed that the left ventricular systolic function was severely impaired and the end-systolic and end-diastolic thickness of the anterior and posterior wall of the left ventricle decreased significantly in TG mice compared with NTG mice (Fig. 1F). Consistent with these results, TG mice exhibited elevated cardiac levels of *Nppa*, *Nppb*, and *Myh7*, which are linked to heart failure (Fig. 1G). Furthermore, the hearts of TG mice exhibited severe fibrosis (Fig. 1H). The findings indicated that the iron-export function of cardiac SLC40A1 is crucial for the maintenance of cardiac iron homeostasis and normal cardiac function.

### Cardiomyocyte-specific overexpression of SLC40A1 elicited alterations of expression of related genes in cardiac iron metabolism, mitochondrial respiratory chain, oxidative stress, and the release of Cytochrome c

**Figure 2:**
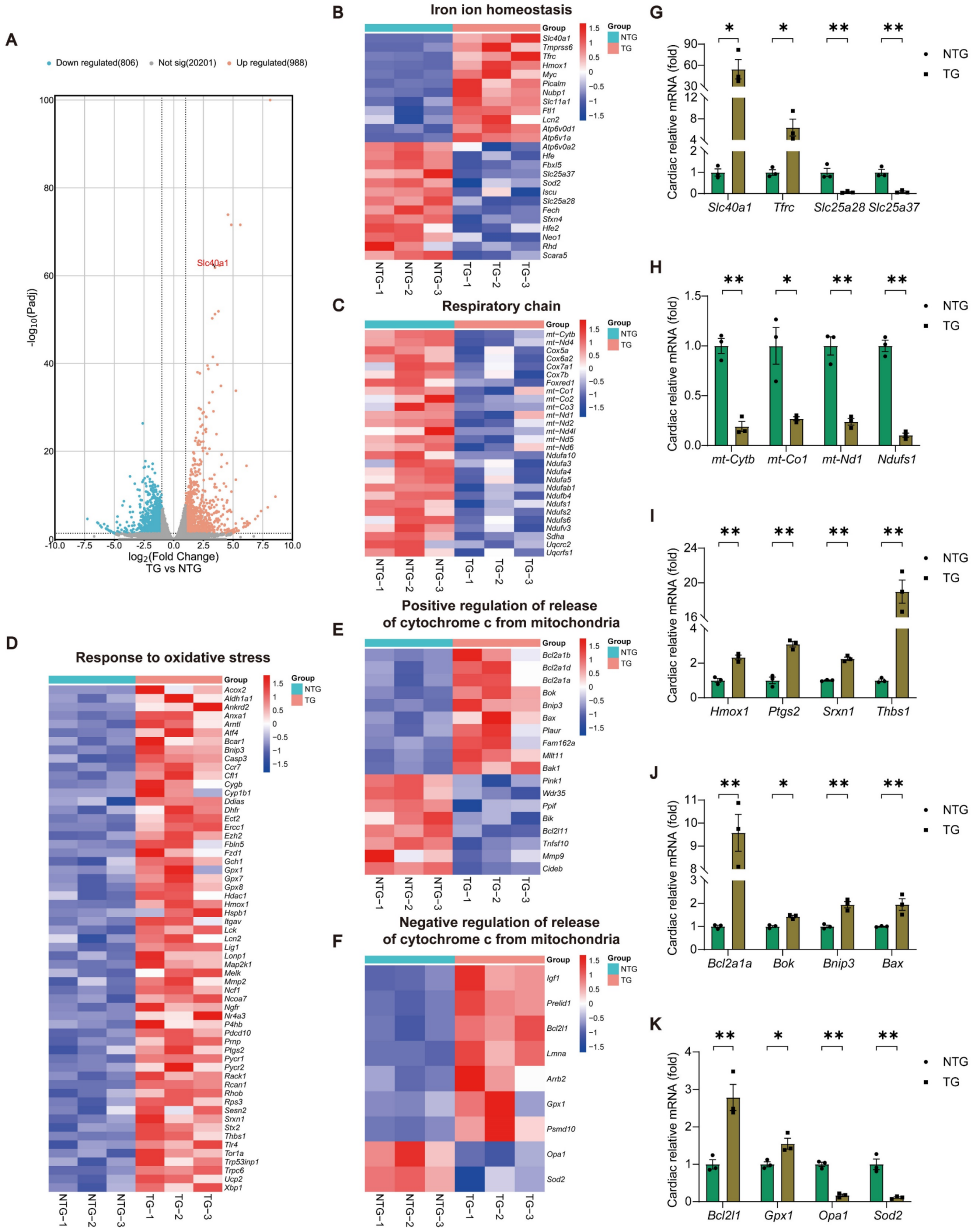
** TG mice myocardia exhibited alterations in the expression of genes related to cardiac iron metabolism, mitochondrial respiratory chain, oxidative stress, and the release of Cytochrome c, in comparison to NTG mice.** (A) A volcano plot is used to illustrate the differential expression of these candidates in relation to all genes (n = 3). *Padj*, *p*.adjust. (B) Heat map displays noteworthy alterations in genes associated with iron ion homeostasis in the GO database (n = 3). (C) Heat map reveals significant down-regulation of genes related to the respiratory chain in the GO database (n = 3). (D) Heat map reveals significant up-regulation of genes related to the response to oxidative stress in the GO database (n = 3). (E, F) Heat map displays noteworthy alterations in genes associated with positive/negative regulation of release of cytochrome c from mitochondria in the GO database (n = 3). (G-K) Validation of the expression of key genes within relevant pathways using qPCR experiment (n = 3). The data is presented as the mean value plus or minus the standard error of the mean. Statistical significance is denoted by *p < 0.05 and **p < 0.01.

In order to determine the molecular mechanism responsible for the progression of HF in TG mice, we conducted RNA-seq analyses of myocardial tissue on both NTG and TG mice (Fig. 2A and Table S5). Through Gene Ontology (GO) enrichment analysis, we observed substantial changes in many pathways closely associated with mitochondrial function, ROS generation, and apoptosis in TG mice (Figure S4A). Consequently, we separately analyzed the expression of related genes with significant changes in iron ion homeostasis, respiratory chain, response to oxidative stress, and positive/negative regulation of release of cytochrome c from mitochondria pathways (Fig. 2B-F). Following this, we performed a qPCR analysis to confirm the expression of crucial genes in related pathways, and noticed consistency between their expression patterns and the sequencing findings (Fig. 2G-K). Based on these findings, we tentatively identified mitochondrial dysfunction, oxidative stress, and apoptosis as the potential significant contributors to HF in TG mice.

### Cardiomyocyte-specific overexpression of SLC40A1 induced myocardial mitochondrial dysfunction, oxidative stress, and apoptosis

**Figure 3:**
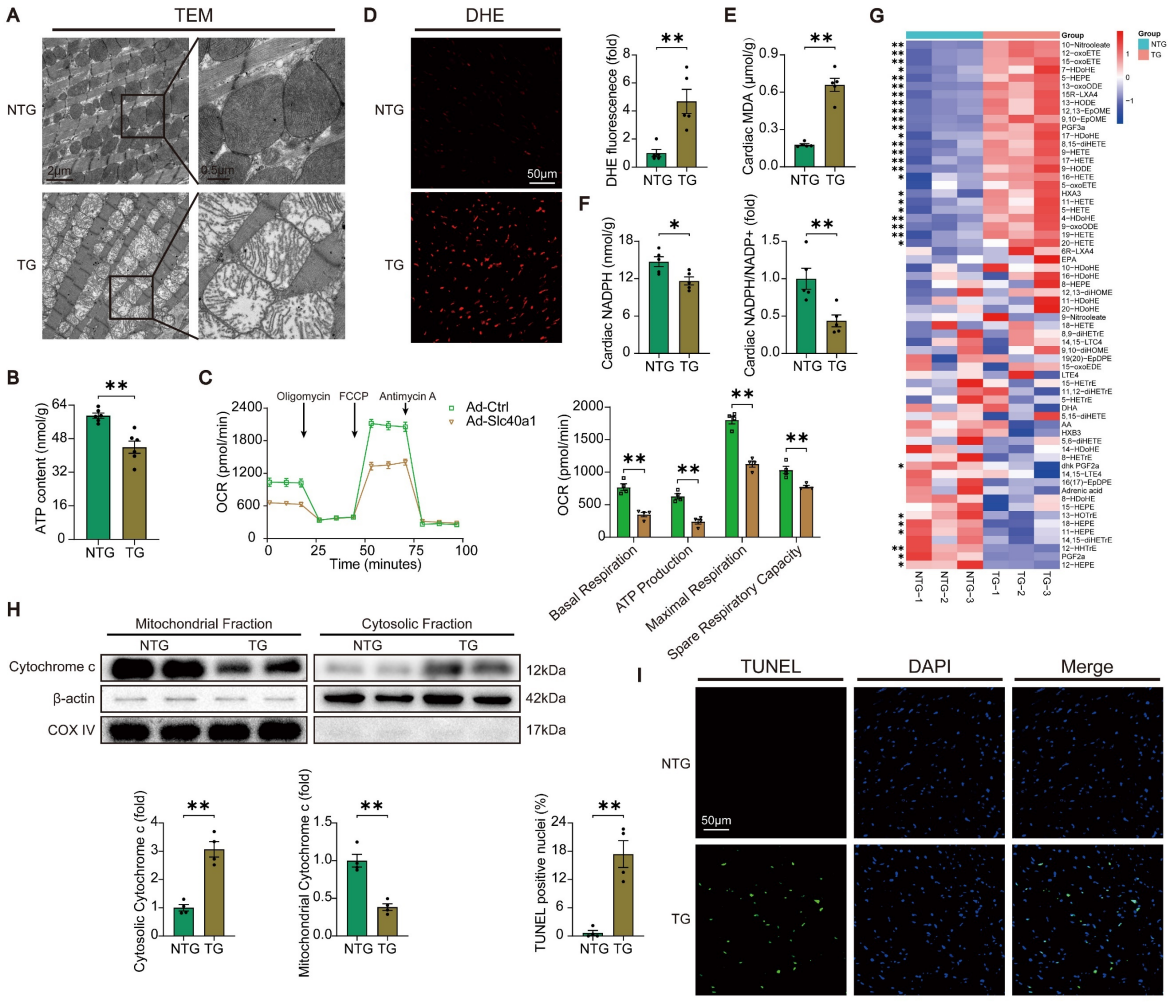
** TG mice myocardia exhibited impaired mitochondrial function, heightened oxidative stress, and increased apoptosis.** (A) Electron micrographs of heart tissue illustrate the morphology of mitochondria. (B) ATP content of heart tissues (n = 6). (C) OCR of primary neonatal cardiomyocytes was measured (n = 4). (D) Representative images and quantitative analysis of DHE staining (n = 5). (E, F) Cardiac MDA and NADPH content were measured in NTG and TG mice (n = 5). (G) Summary of cardiac oxylipins in NTG and TG mice (n = 3). (H) A representative cytochrome c blot and analysis in mitochondrial and cytosolic fractions (n = 4). Loading controls were established using β-actin and Cox IV. (I) Mouse hearts were stained with TUNEL (green) and DAPI (blue) in order to detect apoptotic cells (n = 4). TUNEL positive nuclei are quantified. The data is presented as the mean value plus or minus the standard error of the mean. Statistical significance is denoted by *p < 0.05 and **p < 0.01.

In order to acquire a more profound comprehension of the elements that contribute to heart failure in TG mice, we initially investigated the structure of mitochondria using TEM. TEM analysis at higher magnification showed that mitochondria from hearts of TG mice had reduced electron density and decreased number of mitochondrial cristae (Fig. 3A). Given that normal myocardial contraction necessitates ATP production by mitochondria, we proceeded to evaluate the ATP content and mitochondrial oxygen consumption rate (OCR) as indicators of mitochondrial respiratory capacity. Consistent with expectations, a decrease in ATP content was observed in TG mice. (Fig. 3B). The expression of SLC40A1 in NRCMs was significantly increased by Ad-*Slc40a1*, which was delivered using an adenoviral vector (Figure S6A). The cardiomyocytes treated with Ad-*Slc40a1* showed a significant decrease in mitochondrial respiratory capacity, particularly in basal respiration, ATP production, maximal respiration, and spare respiration, when compared to cardiomyocytes treated with control adenovirus (Ad-Ctrl) (Fig. 3C). Notably, the presence of cardiac iron deficiency in TG mice coincided with severe oxidative stress, as indicated by an increase in DHE intensity and MDA content, a decrease in NADPH levels, and significant variations in many cardiac oxylipins involved in ROS signaling (Fig. 3D-G). However, there was an absence of substantial alteration in the myocardial expression of crucial genes associated with ferroptosis in TG mice (Figure S5A and S5B). Excessive levels of ROS also have the potential to initiate DNA damage and apoptosis. We found that the release of cytochrome c from mitochondria increased in TG mice (Fig. 3H). Consistently, TUNEL tests demonstrated that TG mice exhibited significant apoptosis in comparison to NTG mice (Fig. 3I). Taken together, these data suggested that iron deficiency caused by overexpression of SLC40A1 in cardiomyocytes could induce myocardial mitochondrial dysfunction, oxidative stress, and apoptosis in mice.

### Steap4, one of the interacting proteins of SLC40A1, had the capability to facilitate the efflux of iron from cardiomyocytes

**Figure 4:**
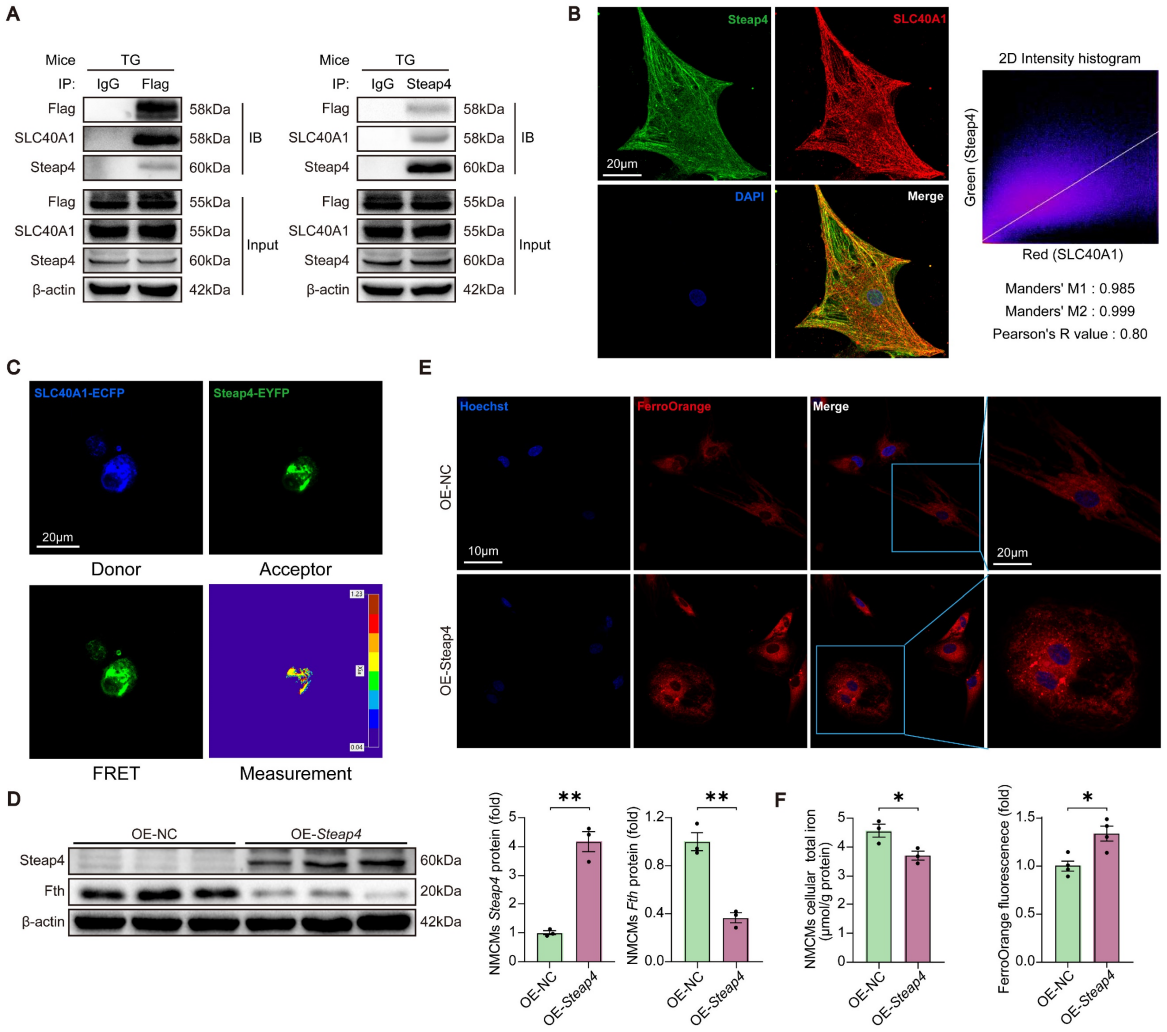
** Steap4 facilitated SLC40A1-mediated iron efflux in cardiomyocytes.** (A) A Co-IP experiment demonstrated SLC40A1 and Steap4 interaction. IB, immunoblot; IP immunoprecipitation. (B) Representative images of immunofluorescence staining for Steap4 (green), SLC40A1 (red), and DAPI (blue) in primary neonatal cardiomyocytes. (C) A strong interaction between SLC40A1 and Steap4 was observed through the FRET experiment (n = 3). (D) NMCMs Steap4 and Fth proteins was measured using western blot analysis (n = 3). NMCMs, neonatal mouse cardiomyocytes. (E) Intracellular Fe^2+^ levels were analyzed fluorometrically using FerroOrange staining (n = 4). (F) Total iron levels in the NMCMs were measured (n = 4). The data is presented as the mean value plus or minus the standard error of the mean. Statistical significance is denoted by *p < 0.05 and **p < 0.01.

It is widely acknowledged that SLC40A1 is the only iron exporter in mammals. However, it is not clear whether other proteins are involved in SLC40A1-mediated iron efflux. We identified several proteins related to iron metabolism as possible SLC40A1 interactors through Co-IP and LC-MS/MS (Table S4). Because SLC40A1 transports Fe^2+^ rather than Fe^3+^, Steap4 (six transmembrane epithelial antigen of prostate 4), a kind of ferrireductase, had attracted our attention. In order to further substantiate the interaction between SLC40A1 and Steap4, a series of supplementary experiments were devised. Initially, immunoprecipitation (IP) assays were conducted using various antibodies. As anticipated, a conspicuous interaction between SLC40A1 and Steap4 was observed (Fig. 4A). Subsequently, immunofluorescence was employed to demonstrate the co-localization of SLC40A1 and Steap4 in primary neonatal cardiomyocytes (Fig. 4B). Meanwhile, the FRET measurement indicated a significant interaction between SLC40A1 and Steap4 (Fig. 4C). Besides, Steap4 was overexpressed in cardiomyocytes in order to elucidate its involvement in iron metabolism. Notably, the overexpression of Steap4 led to the degradation of ferritin, an elevation in Fe^2+^ levels, and a reduction in total iron content in primary neonatal cardiomyocytes (Fig. 4D-F). These findings suggested that Steap4 exhibits ferrireductase activity, thereby facilitating iron efflux in cardiomyocytes.

### SLC40A1 and Steap4 were up-regulated in the ischemic area at initial phase of MI

**Figure 5:**
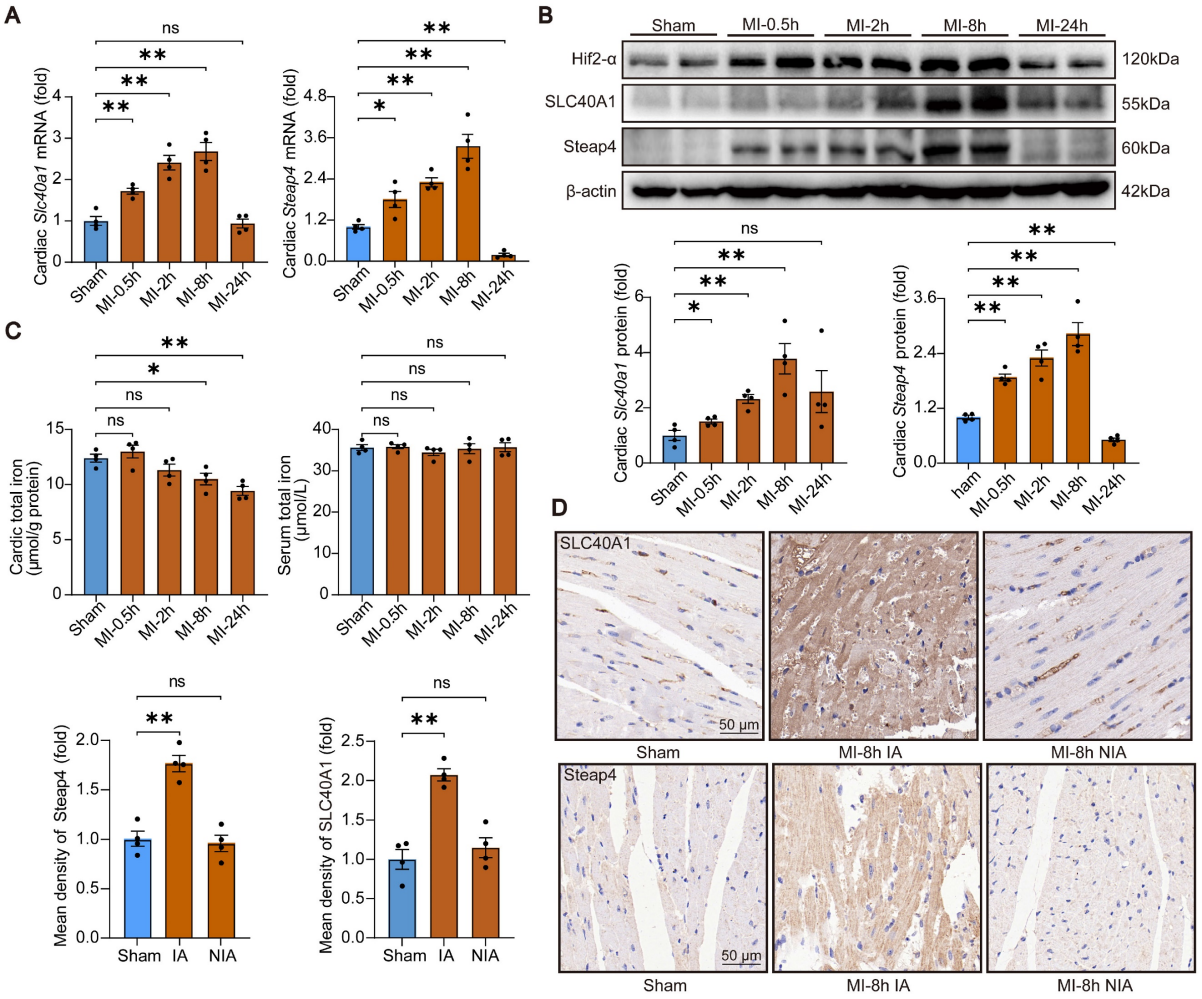
** SLC40A1 and Steap4 were up-regulated in the ischemic area at initial phase of MI.** (A) Expression of *Slc40a1* and *Steap4* mRNA in the heart following MI at various time points or sham surgery was analyzed by qPCR (n = 4). (B) *Slc40a1* and *Steap4* proteins expression in hearts subjected to MI at various time points or sham surgery using Western blotting (n = 4). (C) Heart and serum iron levels were measured after MI and sham surgery (n = 4). (D) Immunohistochemistry of SLC40A1 and Steap4 was quantified and representative images were obtained from both sham-operated and MI mice (n = 4), including the non-infarct area (NIA) and infarct area (IA). The data is presented as the mean value plus or minus the standard error of the mean. Statistical significance is denoted by *p < 0.05 and **p < 0.01.

In order to investigated the involvement of SLC40A1 in cardiovascular disease, an analysis was conducted on publicly accessible RNA sequencing data pertaining to human heart failure. It was observed that the expression of *Slc40a1* was higher in dilated cardiomyopathy (DCM) hearts compared to non-failure (NF) hearts (Figure S7A). Additionally, another analysis revealed elevated mRNA levels of *Slc40a1* in idiopathic dilated cardiomyopathy (IDC) and ischemic cardiomyopathy (ICM) hearts (Figure S7B). Previous studies have reported that both of SLC40A1 and Steap4 expression could be transcriptionally regulated by Hif-2α (hypoxia inducible factor 2, alpha subunit) transcription factor 26-28. In light of these, an experimental model involving adult mice with MI was employed to explore the impact of SLC40A1 and Steap4 on cardiac function. Initially, the expression levels of SLC40A1 and Steap4 were assessed in heart tissues. Notably, both the mRNA and protein levels of SLC40A1 and Steap4 exhibited significant up-regulation at 0.5h, 2h, and 8h post-MI, but not at the 24-hour time point (Fig. 5A and B). This phenomenon may be attributed to the substantial loss of cardiomyocytes in the infarcted region 24 hours after MI. Furthermore, the results of immunohistochemical staining indicated that MI primarily augmented the expression of SLC40A1 and Steap4 in the infarct area, not in non-infarct area (Fig. 5C). As expected, the total iron content in the ischemic myocardium exhibited a significant decrease starting at 8 hours post-MI, while there was no significant change in serum iron (Fig. 5D). Given these results, we hypothesized that ischemia increases the expression of SLC40A1 and Steap4, thereby promoting the efflux of iron from the myocardium. This mechanism ultimately leads to iron deficiency within the ischemic myocardium.

### Cardiomyocyte-specific knockdown of SLC40A1 alleviated cardiac dysfunction after MI in mice

**Figure 6:**
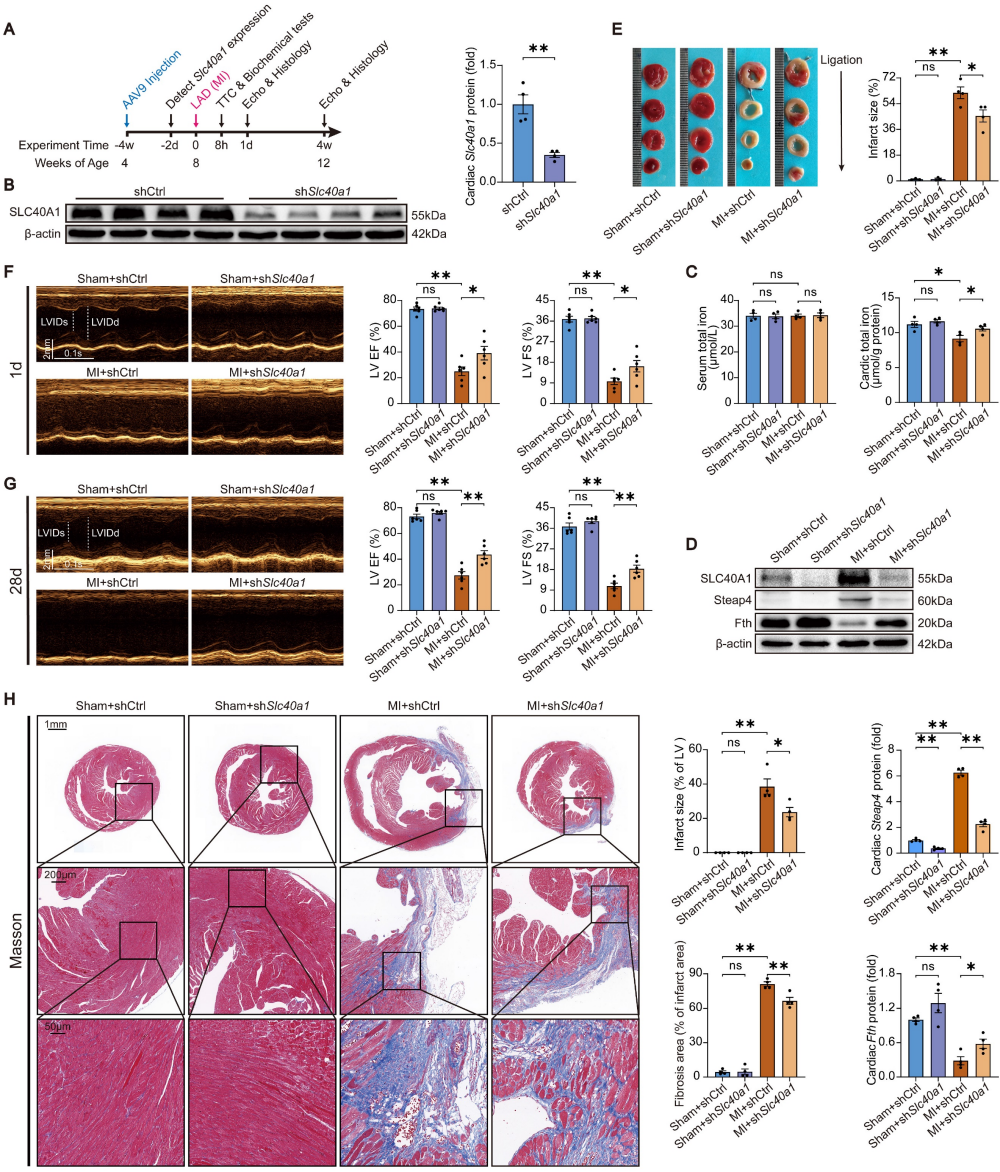
** Knockdown of SLC40A1 in cardiomyocytes alleviated cardiac dysfunction after MI.** (A) *Slc40a1* gene therapy experimental design timeline. (B) Western blotting was performed to assess the expression of heart *Slc40a1* proteins, with associated quantitative analysis (n = 4). (C) Total iron levels in the heart and serum were measured at 8 h post-MI (n = 4). (D) Cardiac *Steap4* and *Fth* proteins were measured using western blot analysis, normalized to β-actin (n = 4). (E) 8 h after MI, infarct size measured by TTC staining (n = 3, 3, 4, 4). (F, G) Long-axis M-mode echocardiogram images and analysis on the first and 28th days after MI (n = 6). (H) Image and quantification of the infarct size and fibrotic area on the 28th day post-MI using Masson trichrome staining (n = 4). The data is presented as the mean value plus or minus the standard error of the mean. Statistical significance is denoted by *p < 0.05 and **p < 0.01.

C57BL/6 J mice were injected with AAV9-cTnT-sh*Slc40a1* or AAV9-cTnT-shCtrl at four weeks of age to examine the influence of iron on myocardial ischemic injury (Fig. 6A). The hearts of mice injected with AAV9-sh*Slc40a1* exhibited a significant decrease in SLC40A1 expression (Fig. 6B). Consistent with expectations, AAV9-mediated knockdown of SLC40A1 hindered the decline in iron content and the degradation of ferritin within 8 hours post-MI (Fig. 6C and D). Excitingly, the sh*Slc40a1* group demonstrated a notable reduction in infarct size in comparison to the shCtrl group, as determined by TTC staining analysis (Fig. 6E). Subsequently, echocardiography was conducted on the first and 28th day following MI. The sh*Slc40a1* group exhibited a significant amelioration of cardiac dysfunction, as indicated by the increased EF and FS values (Fig. 6F and G). The mice with SLC40A1 cardiac knockdown exhibited reduced infarct size and myocardial fibrosis, as shown by Masson trichrome staining, in contrast to the control mice (Fig. 6H). These findings suggested that cardiomyocyte-specific knockdown of SLC40A1 impedes the iron loss in ischemic cardiomyocytes, consequently reducing the infarct size and enhancing cardiac function after MI.

### Cardiomyocyte-specific knockdown of SLC40A1 alleviated myocardial mitochondrial dysfunction, oxidative stress, and apoptosis after MI in mice

**Figure 7:**
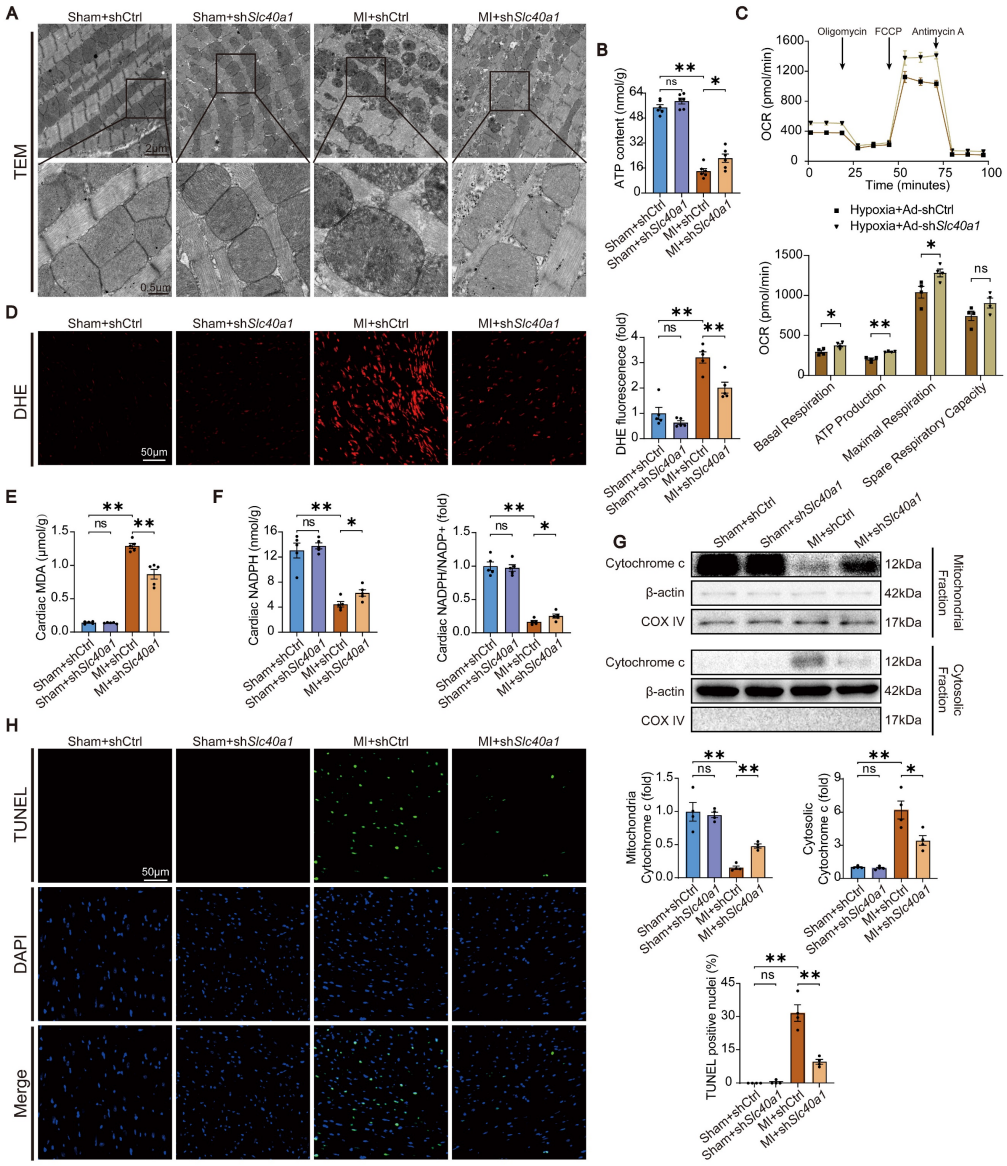
** Knockdown of SLC40A1 in cardiomyocytes alleviated myocardial mitochondrial dysfunction, suppresses oxidative stress, and reduces apoptosis after MI.** (A) Micrographs of mitochondria in heart tissue taken by electron microscopy. (B) ATP content of heart tissues (n = 6). (C) Measurement of OCR and subsequent quantitative analysis in primary neonatal cardiomyocytes (n = 4). (D) Representative images and quantitative analysis of DHE staining (n = 5). (E, F) MDA and NADPH content in the heart were measured (n = 5). (G) A representative cytochrome c blot and analysis in mitochondrial and cytosolic fractions (n = 4). Loading controls were established using β-actin or Cox IV. (H) Mouse hearts were stained with TUNEL (green) and DAPI (blue) in order to detect apoptotic cells (n = 4). TUNEL positive nuclei are quantified. The data is presented as the mean value plus or minus the standard error of the mean. Statistical significance is denoted by *p < 0.05 and **p < 0.01.

The occurrence of myocardial ischemia frequently results in mitochondrial injury, oxidative stress, and apoptosis. This phenomenon bears resemblance to the cardiac injury induced by cardiomyocyte-specific overexpression of SLC40A1. In comparison to the sham controls, TEM imaging showed that the hearts of the mice with MI exhibited swollen mitochondria with disordered cristae (Fig. 7A). Interestingly, knockdown of SLC40A1 alleviated MI-induced swelling of mitochondria and disorder of cristae (Fig. 7A). Alterations in organelle morphological structures are frequently accompanied by corresponding changes in their functions. Therefore, investigations were conducted to assess the mitochondrial respiratory function through the evaluation of ATP content and OCR. We found that knockdown of SLC40A1 significantly decelerated the substantial decline in ATP content following myocardial ischemia (Fig. 7B).

The shRNA-*Slc40a1* adenoviral vector successfully decreased the SLC40A1 expression in NRCMs (Figure S8A). In comparison to cardiomyocytes infected with control-shRNA adenovirus (Ad-shCtrl), the knockdown of SLC40A1 in cardiomyocytes resulted in a significant enhancement of mitochondrial respiratory capacity, encompassing basic respiration, ATP generation, and maximum respiration following hypoxia treatment (Fig. 7C). Interestingly, knockdown of SLC40A1 significantly attenuated the oxidative stress induced by myocardial ischemia for a duration of 8 hours, as evidenced by DHE staining, MDA level detection, and NADPH content determination (Fig. 7D-F). Next, we evaluated apoptosis-associated indicators and noted that knockdown of SLC40A1 resulted in a reduction in the discharge of mitochondrial cytochrome c and the quantity of TUNEL staining positive cells induced by ischemia (Fig. 7G and H). These findings further substantiated that the suppression of iron deficiency in infarcted myocardium through knockdown of SLC40A1 can markedly enhance myocardial anti-ischemic capacity.

### The fatal HF induced by cardiomyocyte-specific overexpression of SLC40A1 was partially rescued by cardiomyocyte-specific knockdown of Steap4

**Figure 8:**
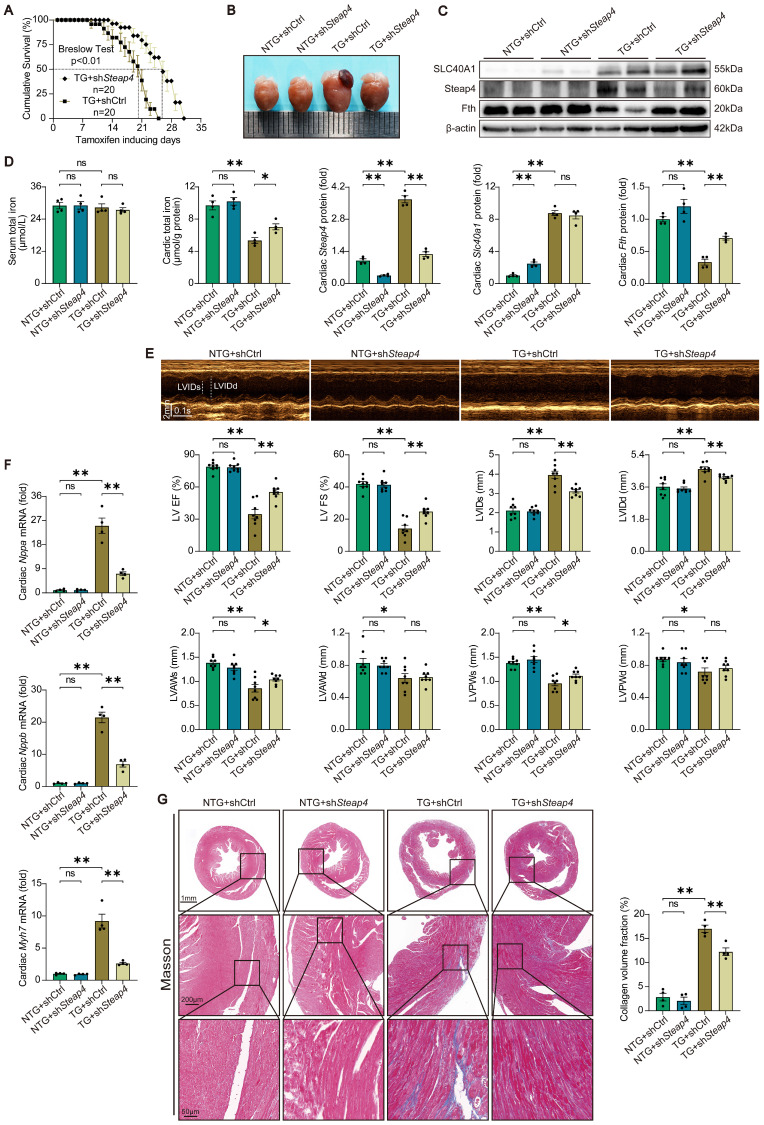
** Knockdown of Steap4 in cardiomyocytes partially rescued the fatal heart failure in TG mice.** (A) Cumulative survival rates of TG mice in shCtrl group (n = 20) and sh*Steap4* (n = 20) group were recorded. (B) Representative images of the heart. (C) Cardiac *Slc40a1*, *Steap4*, and *Fth* proteins were measured using western blot analysis, normalized to β-actin (n = 4). (D) Total iron levels in the heart and serum were measured (n = 4). (E) Long-axis M-mode echocardiogram images and its analysis (n = 8). (F) Cardiac *Nppa*, *Nppb*, *Myh7* mRNA were measured using qPCR, normalized to β-actin (n = 4). (G) Masson trichrome staining images and quantification of fibrotic areas (n = 4). The data is presented as the mean value plus or minus the standard error of the mean. Statistical significance is denoted by *p < 0.05 and **p < 0.01.

To further substantiated that Steap4 plays an important role in SLC40A1-mediated iron efflux, we employed AAV9 to knockdown Steap4 in cardiomyocytes. Specifically, AAV9-cTnT-sh*Steap4* or AAV9-cTnT-shCtrl was administered via intrathoracic injection to genotyped NTG or TG mice. Following a 4-week period post AAV9 injection, the mice were subjected to a tamoxifen-containing diet. Excitingly, cardiomyocyte-specific knockdown of Steap4 exhibited a noteworthy extension in the median survival time of TG mice and a reduction in cardiac enlargement (Fig. 8A and B). Consistent with expectations, the knockdown of Steap4 effectively impeded the degradation of myocardial ferritin and the decline in total iron content in TG mice (Fig. 8C and D). Intriguingly, we found that interference with the expression of SLC40A1 elicits alterations in the level of Steap4 protein (Fig. 6D and Fig. 8C). This observation further underscored the intimate association between Steap4 and SLC40A1. Following this, echocardiography was employed to assess the cardiac function of the mice. The results showed that knockdown of Steap4 considerably mitigated left ventricular contractile dysfunction, dilatation, and thinning of the end-systolic anterior and posterior wall thickness in TG mice (Fig. 8E). Furthermore, the knockdown of Steap4 decelerated the advancement of HF in TG mice, as demonstrated by the reduction in the expression of *Nppa*, *Nppb*, and *Myh7* (Fig. 8F). Additionally, the cardiac fibrosis occurring in TG mice was also alleviated by knocking down Steap4 (Fig. 8G). The morphological irregularities observed in TG mouse myocardial mitochondria, including reduced electron density, decreased ridge count, and vacuolization, can be ameliorated by knocking down Steap4 (Figure S9A). Importantly, the reduction in NADPH levels, which serves as a crucial electron donor for Steap4's ferrireductase activity, was significantly attenuated in TG mice upon Steap4 knockdown (Figure S9B). Lastly, the knockdown of Steap4 resulted in a decrease in myocardial apoptosis in TG mice (Figure S9C). These findings provided additional evidence supporting the significance of Steap4 as a facilitator in the iron efflux function of SLC40A1.

## Discussion

**Figure 9:**
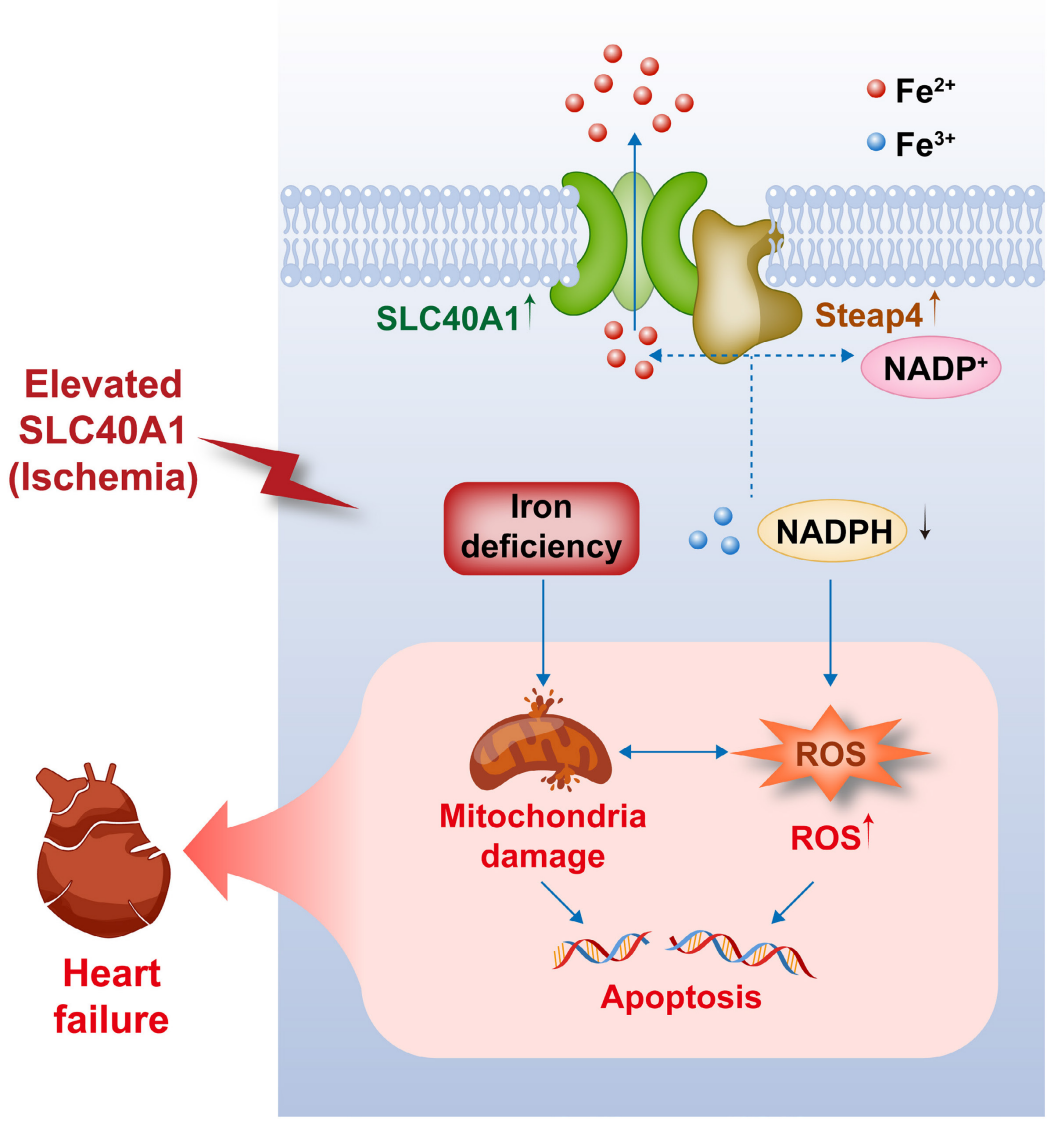
** This schematic diagram elucidates the pathways by which an ischemia-induced upregulation of SLC40A1 and Steap4 exacerbated mitochondrial dysfunction, oxidative stress, and apoptosis, thereby contributing to the progression of heart failure.** The upregulation of SLC40A1 and Steap4, induced by ischemia, promotes the efflux of iron from cardiomyocytes. Consequently, the reduction in iron and NADPH content within cardiomyocytes further exacerbates mitochondrial dysfunction and enhances the accumulation of ROS. This cascade of events culminates in cell apoptosis, ultimately leading to the onset of heart failure.

Our study yielded several significant findings. First, we observed that the overexpression of SLC40A1, specifically in cardiomyocytes, led to severe iron deficiency and induced mitochondrial dysfunction, oxidative stress, and apoptosis, subsequently resulting in the development of fatal HF in mice. Second, we discovered that Steap4 interacted with SLC40A1 and facilitated iron efflux, thereby jointly regulating iron balance in cardiomyocytes. Third, we observed an upregulation of both SLC40A1 and Steap4 in the ischemic area during the initial phase of MI, accompanied by a decrease in iron content. Additionally, the cardiomyocyte-specific knockdown of SLC40A1 was found to ameliorate cardiac dysfunction following MI by enhancing mitochondrial function, suppressing oxidative stress, and diminishing cardiomyocyte apoptosis. Therefore, targeting SLC40A1 may provide a new treatment strategy for HF following MI (Fig. 9).

Iron is a vital trace element that plays a crucial role in various essential intracellular processes owing to its chemical properties facilitating its involvement in redox reactions 29. Given the high-energy requirements of cardiomyocytes, iron metabolism is particularly important in these cells. Nevertheless, the free form of unbound iron is a highly reactive element that generates ROS and induces oxidative stress through the Fenton reaction, posing a potential risk to cellular integrity 24. Therefore, iron metabolism in cardiomyocytes is subject to stringent regulation. Cardiomyocytes primarily take up iron via Tfrc (transferrin receptor) located in the cell membrane, facilitating receptor-mediated endocytosis of serum transferrin 30. Within endosomes, Steap3 (six-transmembrane epithelial antigen of the prostate 3) catalyzes the reduction of Fe^3+^ to Fe^2+^, which is subsequently transported to the cytosol via Slc11a2 (solute carrier family 11 member 2). Then, transferrin and Tfrc are recycled to the cell surface 31. The absence of Tfrc in cardiac tissues leads to cardiac hypertrophy in mice, which is attributable to profound iron deficiency and concomitant mitochondrial dysfunction 32. Fth (Ferritin heavy chain) plays a central role in cardiomyocyte iron storage. Mice lacking Fth, specifically in cardiomyocytes, demonstrate decreased levels of cardiac iron and heightened oxidative stress 24.

As previously stated, SLC40A1 is the only identified iron-exporting protein in mammals. The specific removal of SLC40A1 in cardiomyocytes through the utilization of Myh6-Cre leads to an excessive accumulation of iron in the heart, causing impairment of cardiac function and reduced lifespan 33. Conversely, other researchers have not observed apparent abnormalities in cardiac tissues derived from conditional SLC40A1-deficient mice bred with MCK-Cre 34. However, our study revealed that the knockdown of SLC40A1 in cardiomyocytes using AAV9 served as a favorable treatment for MI. The expression of MCK-Cre recombinase in cardiomyocytes begins later than that of Myh6-Cre recombinase. These findings suggest that the downregulation of SLC40A1 is well tolerated by the cardiomyocytes in adult mice. To elucidate the role of SLC40A1 in the myocardium, we constructed a TG model of cardiomyocyte-specific SLC40A1 overexpression in adult mice. Our findings revealed that TG mice exhibited more severe HF and shorter median survival than mice with cardiomyocyte-specific SLC40A1 knockout reported by Lakhal-Littleton et al 33. This implies that myocardial iron deficiency leads to more extensive myocardial damage than iron overload.

Steap4, also referred to as STAMP2 or TIARP, belongs to the STEAP family and is present in various cellular membrane structures, such as the cell membrane, endosome, and Golgi apparatus 35. It functions as a ferrireductase that converts Fe^3+^ to Fe^2+^ via NADPH utilization 36. The role of Steap4 in the myocardium remains unexplored, and its association with SLC40A1 remains unclear. Previous studies suggested that the iron excreted by SLC40A1 originates from the labile iron pool 29, 37. However, to our knowledge, our study is the first to reveal an interaction between SLC40A1 and Steap4, which facilitated SLC40A1-mediated iron efflux. It has been documented that Steap4, unlike Steap3 which promotes iron uptake 38, promotes intracellular iron excretion 39, 40. These results support our findings. Furthermore, our findings indicated that the modulation of SLC40A1 expression resulted in a significant alteration in the protein levels of Steap4. This observation supports a strong association between Steap4 and SLC40A1. Previous studies reported that elevated Steap4 expression can induce oxidative stress in cells 26, 41. This correlation is expected as both the depletion of NADPH and the generation of Fe^2+^ are linked to cellular oxidative stress.

The overexpression of SLC40A1 in cardiomyocytes resulted in iron deficiency, along with the induction of mitochondrial dysfunction, oxidative stress, and apoptosis. Conversely, the cardiomyocyte-specific knockdown of SLC40A1 has been found to ameliorate mitochondrial dysfunction, oxidative stress, and apoptosis following MI. Iron deficiency can induce mitochondrial dysfunction in cardiomyocytes owing to the significant requirement of iron for electron transfer within the mitochondria 42, 43. Iron deficiency also impairs the antioxidant capacity of cardiomyocytes, resulting in ROS accumulation. Thus, mitochondrial damage and ROS accumulation can induce apoptosis. In addition, our study revealed that the cardiomyocyte-specific overexpression of SLC40A1 induces severe oxidative stress, implicating the involvement of Steap4 in this mechanism.

Ischemic cardiomyopathy is the principal etiology of HF. MI causes the remaining cardiomyocytes to die or become dysfunctional when the blood supply is suddenly interrupted or reduced 11. Multiple studies have demonstrated that the re-establishment of blood flow in the coronary arteries following ischemia can result in an increase in the iron content within the cardiac tissue 44-47. Intramyocardial hemorrhage is a frequently observed complication of reperfusion and serves as the primary factor contributing to heightened levels of iron in the heart 12, 48. Iron deposition after intramyocardial hemorrhage can induce inflammation, edema, and lipid accumulation, thereby expediting the progression of HF after MI 12, 48-50. However, several studies have demonstrated that the administration of iron chelators does not yield the intended outcome of diminishing infarct size 14, 51. Conversely, iron deficiency exacerbates adverse ventricular remodeling after reperfusion 15. These investigations underscore the intricate nature of iron metabolism in the myocardium following I/R injury, necessitating a comprehensive evaluation of both myocardial and microenvironmental iron levels. Furthermore, it is important to differentiate ischemia and I/R injury as distinct pathological processes. A recent study provided evidence supporting the beneficial effects of iron supplementation after MI 52. In our study, we found that SLC40A1 and Steap4 were upregulated in the ischemic areas during the initial phase of MI. As expected, the total iron content in the ischemic myocardium significantly decreased starting at 8 h post-MI. Importantly, the targeted knockdown of SLC40A1 in cardiomyocytes effectively prevented iron loss and reduced the myocardial infarct size. Based on these findings, it may be justifiable to proactively administer iron supplementation during the initial post-MI phase.

The acknowledgment of our study's limitations is necessary. First, the reduction of Fe^3+^ to Fe^2+^ could not be directly observed. Additional, further investigations are needed to understand the reciprocal regulation between SLC40A1 and Steap4.

## Conclusions

To our knowledge, this is the first study to demonstrate that iron loss induced by SLC40A1 upregulation in the ischemic myocardium exacerbated myocardial injury by exacerbating mitochondrial dysfunction, oxidative stress, and apoptosis. Mechanistically, Steap4 interacted with SLC40A1 and facilitated iron efflux. Furthermore, we revealed that cardiomyocyte-specific overexpression of SLC40A1 can lead to fatal HF in mice. Therefore, targeting SLC40A1 may provide a new treatment strategy for HF following MI.

## Supplementary Material

Supplementary figures and tables.
