# Supplementary material for: Elevated SLC40A1 impairs cardiac function and exacerbates mitochondrial dysfunction, oxidative stress, and apoptosis in ischemic myocardia
Source: Int J Biol Sci. 2024 Jan 1;20(2):414–32. doi: 10.7150/ijbs.89368 (PMC10758104; doi:10.7150/ijbs.89368)
Supplement: Supplementary file 1 — Supplementary figures and tables. [file ijbsv20p0414s1.pdf]

Supplementary materials

Table S1

The primer sequences used for quantitative PCR were as follows:

Species	Gene	Forward primer	Reverse primer
Mouse	*Bax*	TGAAGACAGGGGCCTTTTTG	AATTCGCCGGAGACACTCG
Mouse	*Bcl2a1a*	GGCTGAGCACTACCTTCAGTA	TGGCGGTATCTATGGATTCCAC
Mouse	*Bcl2l1*	AGTGGAGGTACACCCCTCAG	AAGGCTGGGATCACAAACGTG
Mouse	*Bnip3*	TCCTGGGTAGAACTGCACTTC	GCTGGGCATCCAACAGTATTT
Mouse	*Bok*	AGGTAGTGTCCCTGTATTCCG	AAGGTCTTGCGTACAAACTCC
Mouse	*Gpx1*	AGTCCACCGTGTATGCCTTCT	GAGACGCGACATTCTCAATGA
Mouse	*Hmox1*	GGTGATGGCTTCCTTGTACC	AGTGAGGCCCATACCAGAAG
Mouse	*mt-co1*	TCGGAGCCCCAGATATAGCA	TTTCCGGCTAGAGGTGGGTA
Mouse	*mt-cytb*	GGCTACGTCCTTCCATGAGG	AGCGAAGAATCGGGTCAAGG
Mouse	*mt-nd1*	TCCGAGCATCTTATCCACGC	GTATGGTGGTACTCCCGCTG
Mouse	*Myh7*	ACTGTCAACACTAAGAGGGTCA	TTGGATGATTTGATCTTCCAGGG
Mouse	*Ndufs1*	AGGATATGTTCGCACAACTGG	TCATGGTAACAGAATCGAGGGA
Mouse	*Nppa*	TGACAGGATTGGAGCCCAGAG	AGCTGCGTGACACACCACAAG
Mouse	*Nppb*	GCTGCTTTGGGCACAAGATAG	GGTCTTCCTACAACAACTTCA
Mouse	*Opa1*	ACAGCAAATTCAAGAGCACGA	TTGCGCTTCTGTTGGGCAT
Mouse	*Ptgs2*	CTGCGCCTTTTCAAGGATGG	GGGGATACACCTCTCCACCA
Mouse	*Slc25a28*	AGCATTGCGTGATGTACCCG	CCTGTTGCTGTGACGTTCA
Mouse	*Slc25a37*	CGGAGGATGGATGGGGACT	GCTGGTCGGTAGGTTCTCGTA
Mouse	*Slc40a1*	GTGGAGTACTTCTTGCTCTGG	CTGCTTCAGTTCTGACTCCTC
Mouse	*Sod2*	CAGACCTGCCTTACGACTATGG	CTCGGTGGCGTTGAGATTGTT
Mouse	*Srxn1*	CCCAGGGTGGCGACTACTA	GTGGACCTCACGAGCTTGG
Mouse	*Steap4*	GGGAAGTCACTGGGATTGAAAA	CCGAATAGCTCAGGACCTCTG
Mouse	*Tfrc*	CTCAGTTTCCGCCATCTCAGT	GCAGCTCTTGAGATTGTTTGCA
Mouse	*Thbs1*	GGGGAGATAACGGTGTGTTTG	CGGGGATCAGGTTGGCATT
Mouse	*β-Actin*	GTCCCTCACCCTCCCAAAAG	GCTGCCTCAACACCTCAACCC

Table S2

Primary antibodies used for western blotting, immunoprecipitation, immunofluorescence, and immunohistochemistry were as follows:

Antibody	Host	Working dilutions	Catalog No.	Supplier
Flag	Mouse	WB: 1/1000<br>IP: 1/100	MA1-91878	Thermo Fisher Scientific, USA
Flag	Rabbit	WB: 1/2000	ab205606	Abcam, UK
SLC40A1	Rabbit	WB: 1/1000	NBP1-21502	Novus, USA
SLC40A1	Mouse	WB: 1/1000	ab239583	Abcam, UK

Steap4	Rabbit	IHC: 1/100<br>IF: 1/100<br>WB: 1/500<br>IP: 1/50	11944-1-AP	Proteintech, China
Steap4	Goat	IHC: 1/50<br>WB: 1/500<br>IF: 1/100	NB100-68162	Novus, USA
Fth	Rabbit	WB: 1/1000	A19544	ABclonal, China
Hif2-α	Rabbit	WB: 1/1000	A7553	ABclonal, China
Cox-IV	Rabbit	WB: 1/5000	11242-1-AP	Proteintech, China
Cytochrome c	Rabbit	WB: 1/5000	ab133504	Abcam, UK
β-Actin	Rabbit	WB: 1/5000	81115-1-RR	Proteintech, China
Gpx4	Rabbit	WB: 1/1000	A11243	ABclonal, China

Table S3

shRNA target sequences were as follows:

**Target**	**Species**	**Target sequences**
Slc40a1	Mouse	GTGGATCCATCCTTAGTATTT
Steap4	Mouse	CAGAGTCAAATGCGGAATA
Slc40a1	Rat	TCTTACACAAGAATGAGCTTCTGAA (1)
Slc40a1	Rat	TAACATCCGTGAACTTGAATGTGAA (2)
Slc40a1	Rat	CGAGATGGATGGGTCTCCTACTATA (3)

Table S4

Mass spectrum analysis of Flag (SLC40A1)-interacting proteins

**Protein name**	**Unique peptides**	**Molecular mass (kDa)**	**Abundance**	**MS score**
SLC40A1	10	62.7	2.3×10^8	41
Steap4	5	53	3.7×10^6	9
Tfrc	5	85.7	1.5×10^6	4
Ndufs4	2	19.8	8.5×10^5	6
Abcb7	2	82.5	2.4×10^5	0

Following Co-IP assays of TG mouse heart tissue and electrophoresis, the whole band (Flag-interacting proteins) assessed by LC-MS/MS. Tfrc, Transferrin receptor protein 1; Ndufs4, NADH dehydrogenase [ubiquinone] iron-sulfur protein 4, mitochondrial; Abcb7, Iron-sulfur clusters transporter Abcb7, mitochondrial.

Table S5

List of genes that were significantly up-regulated (Fold change＞0)/down-regulated (Fold change＜0) in TG mice compared to NTG mice based on RNA-seq analysis (Padj＜0.5)

Gene	ID	Fold change	Padj
Nmrk2	ENSMUSG00000004939	8.117495592	0
Postn	ENSMUSG00000027750	4.56749557	1.32E-74
Gdf15	ENSMUSG00000038508	5.608992285	2.72E-72
Asns	ENSMUSG00000029752	4.845394302	2.72E-72
Ctgf	ENSMUSG00000019997	3.852524426	5.09E-63
Slc40a1	ENSMUSG00000025993	3.472875306	1.55E-62
Col8a1	ENSMUSG00000068196	3.765430053	1.33E-52
Atf3	ENSMUSG00000026628	3.473179931	5.97E-52
Meox1	ENSMUSG00000001493	3.242655617	5.30E-51
Mthfd2	ENSMUSG00000005667	3.300745885	3.30E-42
Thbs1	ENSMUSG00000040152	3.677770777	1.89E-40
Cdkn1a	ENSMUSG00000023067	2.843242936	3.04E-40
Eln	ENSMUSG00000029675	2.91912677	1.83E-39
Rcan1	ENSMUSG00000022951	2.000307896	1.00E-38
Fhl1	ENSMUSG00000023092	2.196092801	1.82E-38
Timp1	ENSMUSG00000001131	3.975318782	1.31E-35
Tnc	ENSMUSG00000028364	5.238270091	1.68E-34
Phgdh	ENSMUSG00000053398	3.26872831	3.35E-34
Tfrc	ENSMUSG00000022797	2.984757092	8.96E-32
Col3a1	ENSMUSG00000026043	2.118238904	1.55E-30
Hmox1	ENSMUSG00000005413	2.266246653	2.87E-30
Lgals3	ENSMUSG00000050335	3.34316456	8.23E-30
Loxl2	ENSMUSG00000034205	2.521607813	1.29E-29
Uck2	ENSMUSG00000026558	2.441825395	2.27E-29
Tnfrsf12a	ENSMUSG00000023905	2.704975841	1.36E-28
Adamtsl2	ENSMUSG00000036040	2.744419469	1.70E-28
Emp1	ENSMUSG00000030208	2.196201045	4.54E-28
Gm29216	ENSMUSG00000101249	-2.610648509	4.55E-27
Abra	ENSMUSG00000042895	2.420241512	1.03E-26
Otud1	ENSMUSG00000043415	2.270436428	2.41E-26
Psat1	ENSMUSG00000024640	2.727379357	8.49E-26
Nr4a1	ENSMUSG00000023034	2.626090237	1.38E-25
Mfap5	ENSMUSG00000030116	2.707412893	3.91E-25
Ddit4	ENSMUSG00000020108	2.005292345	5.10E-25
Egln3	ENSMUSG00000035105	2.46871723	9.63E-25
Pycr1	ENSMUSG00000025140	3.595876973	1.28E-24
Fkbp5	ENSMUSG00000024222	1.875809062	9.13E-24
Sparc	ENSMUSG00000018593	2.110127471	4.13E-23
Tuba1a	ENSMUSG00000072235	1.809697335	4.28E-23

Fbn1	ENSMUSG00000027204	2.487849683	6.45E-23
Fstl1	ENSMUSG00000022816	2.278901025	7.63E-23
Cebpd	ENSMUSG00000071637	2.013648875	1.18E-22
Col5a2	ENSMUSG00000026042	2.183033629	3.06E-22
Cyr61	ENSMUSG00000028195	2.031796172	3.42E-22
Gm13889	ENSMUSG00000087006	2.655410771	1.95E-21
Wisp2	ENSMUSG00000027656	2.431831737	1.95E-21
Tyms	ENSMUSG00000025747	3.00543714	2.82E-21
Dbn1	ENSMUSG00000034675	2.473087793	8.69E-21
Col1a2	ENSMUSG00000029661	1.660053418	1.74E-20
Rflnb	ENSMUSG00000020846	1.655433681	1.95E-20
Col4a1	ENSMUSG00000031502	1.709711772	3.20E-20
Serpine1	ENSMUSG00000037411	2.817375581	3.66E-20
Pclaf	ENSMUSG00000040204	4.042724322	5.66E-20
Nr4a3	ENSMUSG00000028341	4.0299369	6.48E-20
Col1a1	ENSMUSG00000001506	1.674452839	4.60E-19
Sgk1	ENSMUSG00000019970	1.785017433	8.91E-19
Mfap4	ENSMUSG00000042436	2.211971972	8.91E-19
Oaf	ENSMUSG00000032014	1.71008253	9.31E-19
Hbegf	ENSMUSG00000024486	1.778233868	1.62E-18
Inmt	ENSMUSG00000003477	-2.386909946	1.75E-18
Apold1	ENSMUSG00000090698	2.321757706	2.43E-18
Egr2	ENSMUSG00000037868	4.484918296	5.60E-18
Entpd5	ENSMUSG00000021236	-1.77391649	7.37E-18
C1qtnf6	ENSMUSG00000022440	2.280506528	8.63E-18
Maff	ENSMUSG00000042622	2.221793096	9.44E-18
Phlda3	ENSMUSG00000041801	1.78751192	9.67E-18
Aspn	ENSMUSG00000021388	1.987810993	1.03E-17
Trib3	ENSMUSG00000032715	2.151958076	1.18E-17
Ptn	ENSMUSG00000029838	2.140089197	1.40E-17
Tubb6	ENSMUSG00000001473	1.614755365	1.49E-17
Sprr1a	ENSMUSG00000050359	6.127593852	2.15E-17
Ppl	ENSMUSG00000039457	-2.111324319	3.23E-17
Spp1	ENSMUSG00000029304	4.01204838	3.68E-17
Gm49477	ENSMUSG00000116066	-2.432555088	3.69E-17
Gal3st3	ENSMUSG00000047658	-2.518152682	5.54E-17
Igf1	ENSMUSG00000020053	1.765006168	7.30E-17
Ivd	ENSMUSG00000027332	-1.793960993	1.20E-16
Prelid1	ENSMUSG00000021486	1.475315086	1.45E-16
Pfkp	ENSMUSG00000021196	2.1498582	1.51E-16

Atp6v1h	ENSMUSG00000033793	1.457308629	3.40E-16
Haus8	ENSMUSG00000035439	1.830560757	3.96E-16
Csrp2	ENSMUSG00000020186	2.060650328	4.53E-16
Col4a2	ENSMUSG00000031503	1.515177102	5.47E-16
Kdelr3	ENSMUSG00000010830	1.832464337	6.42E-16
Tpm4	ENSMUSG00000031799	1.517363756	6.46E-16
Ppip5k2	ENSMUSG00000040648	-1.583851943	9.29E-16
Errfi1	ENSMUSG00000028967	2.22237854	1.31E-15
Tmsb10	ENSMUSG00000079523	1.630816988	1.82E-15
Cdk1	ENSMUSG00000019942	3.425120377	2.33E-15
Sars	ENSMUSG00000068739	1.419923462	2.53E-15
Sik1	ENSMUSG00000024042	1.971056368	2.58E-15
Mif	ENSMUSG00000033307	1.838017834	4.11E-15
Cngb3	ENSMUSG00000056494	-3.679803989	5.51E-15
Myoc	ENSMUSG00000026697	-2.204163932	6.28E-15
9230112J17Rik	ENSMUSG00000111761	-2.448752528	1.44E-14
Sphk1	ENSMUSG00000061878	3.029524048	1.88E-14
Cyb5r1	ENSMUSG00000026456	1.368820525	1.88E-14
Gdf6	ENSMUSG00000051279	3.206003313	1.95E-14
Dhdh	ENSMUSG00000011382	-2.016650638	2.23E-14
Serpinb1c	ENSMUSG00000079049	5.232246153	3.11E-14
Anln	ENSMUSG00000036777	2.523117397	3.86E-14
Acot1	ENSMUSG00000072949	-2.556733626	4.11E-14
Rgcc	ENSMUSG00000022018	1.430428408	5.42E-14
Gm10925	ENSMUSG00000100862	-2.008978321	8.76E-14
Tubb2a	ENSMUSG00000058672	1.702381139	9.28E-14
Fah	ENSMUSG00000030630	-1.790681407	1.01E-13
Vcan	ENSMUSG00000021614	1.936405874	1.02E-13
Plxnb1	ENSMUSG00000053646	-2.635423682	1.05E-13
Xirp2	ENSMUSG00000027022	1.736187817	1.09E-13
Nnmt	ENSMUSG00000032271	1.679850093	1.11E-13
Bnip3	ENSMUSG00000078566	1.397733868	1.33E-13
Cyth3	ENSMUSG00000018001	1.530621653	1.39E-13
Ero1l	ENSMUSG00000021831	1.719582547	1.59E-13
Cabcoco1	ENSMUSG00000019945	-2.368332236	2.23E-13
Col8a2	ENSMUSG00000056174	4.225435161	2.28E-13
Aldob	ENSMUSG00000028307	-3.278950997	2.59E-13
Synpo2l	ENSMUSG00000039376	1.790956113	2.66E-13
11-Sep	ENSMUSG00000058013	1.45191982	2.74E-13
Egr3	ENSMUSG00000033730	3.461639189	3.06E-13

Bok	ENSMUSG00000026278	1.727150983	3.07E-13
Itgbl1	ENSMUSG00000032925	1.796165459	3.11E-13
Anxa2	ENSMUSG00000032231	1.464742549	3.22E-13
Mmp14	ENSMUSG00000000957	1.872883816	4.01E-13
Tbx5	ENSMUSG00000018263	-1.713117388	4.17E-13
Adhfe1	ENSMUSG00000025911	-1.598261089	4.63E-13
Lpar1	ENSMUSG00000038668	1.703619189	5.03E-13
Ms4a7	ENSMUSG00000024672	2.03625247	5.62E-13
Loxl1	ENSMUSG00000032334	1.382925211	8.10E-13
Gstm1	ENSMUSG00000058135	-1.327095894	8.79E-13
Armh4	ENSMUSG00000036242	-1.983720612	8.97E-13
Fyco1	ENSMUSG00000025241	-1.849593425	8.97E-13
Tuba1b	ENSMUSG00000023004	1.277990381	9.38E-13
Tmem45a	ENSMUSG00000022754	2.071894079	9.43E-13
Rbfox1	ENSMUSG00000008658	-2.305031339	9.51E-13
Cilp	ENSMUSG00000042254	4.39949352	1.12E-12
C3	ENSMUSG00000024164	-1.545387431	1.27E-12
Pbk	ENSMUSG00000022033	3.437547272	1.32E-12
S100a10	ENSMUSG00000041959	1.515290181	1.32E-12
Tubb5	ENSMUSG00000001525	1.253814273	1.48E-12
Rhoc	ENSMUSG00000002233	1.419805216	1.76E-12
Col12a1	ENSMUSG00000032332	3.589358828	1.77E-12
Dclk3	ENSMUSG00000032500	3.457943615	1.79E-12
Il15	ENSMUSG00000031712	-1.722432308	2.08E-12
Mxra7	ENSMUSG00000020814	1.439333583	3.41E-12
Abcc9	ENSMUSG00000030249	-1.894081523	3.73E-12
Gm26917	ENSMUSG00000097971	1.9717034	4.63E-12
Gm6416	ENSMUSG00000114792	-3.033731734	5.70E-12
Dusp7	ENSMUSG00000053716	-1.593002846	6.28E-12
Acad12	ENSMUSG00000042647	-1.494523969	6.28E-12
Tpm2	ENSMUSG00000028464	1.732507931	6.28E-12
Gm4544	ENSMUSG00000116056	-2.873690438	7.21E-12
Ppm1k	ENSMUSG00000037826	-1.898731281	7.23E-12
Nts	ENSMUSG00000019890	2.455192799	7.63E-12
Mcm5	ENSMUSG00000005410	1.91588827	8.25E-12
Lox	ENSMUSG00000024529	3.875834615	8.82E-12
Cmya5	ENSMUSG00000047419	-1.718256199	9.29E-12
Tubb2b	ENSMUSG00000045136	2.622385667	1.11E-11
Cldn5	ENSMUSG00000041378	1.628792959	1.33E-11
Gars	ENSMUSG00000029777	1.411127541	1.33E-11

Tpx2	ENSMUSG00000027469	2.640210751	1.41E-11
Anxa3	ENSMUSG00000029484	1.420059533	1.42E-11
Frzb	ENSMUSG00000027004	2.105172189	1.48E-11
Ano10	ENSMUSG00000037949	-1.784401593	1.48E-11
Egflam	ENSMUSG00000042961	-2.335382516	1.55E-11
Acox2	ENSMUSG00000021751	4.858947486	1.60E-11
Selenbp1	ENSMUSG00000068874	-1.168666421	1.65E-11
Asb14	ENSMUSG00000021898	-1.579243505	1.65E-11
Ccl9	ENSMUSG00000019122	1.402952026	1.66E-11
Gsn	ENSMUSG00000026879	-1.476182148	1.67E-11
Pde4a	ENSMUSG00000032177	-1.655132527	1.98E-11
Kcnj2	ENSMUSG00000041695	-2.212258578	2.05E-11
Serp1	ENSMUSG00000027808	1.241412622	2.28E-11
Rps12-ps4	ENSMUSG00000109509	1.621811425	2.28E-11
Nes	ENSMUSG00000004891	1.757435917	2.65E-11
Rpl3	ENSMUSG00000060036	1.522766486	2.87E-11
Rtn4	ENSMUSG00000020458	1.346564989	3.75E-11
Glipr2	ENSMUSG00000028480	1.631057611	3.81E-11
Eif4a1	ENSMUSG00000059796	1.04099584	3.81E-11
Ldha	ENSMUSG00000063229	1.540225253	3.81E-11
Capg	ENSMUSG00000056737	1.520161351	3.81E-11
Ankrd1	ENSMUSG00000024803	1.776662718	3.85E-11
Camk2a	ENSMUSG00000024617	-2.078692272	4.43E-11
Bckdhb	ENSMUSG00000032263	-1.341134688	5.67E-11
Rbm3	ENSMUSG00000031167	1.907718239	5.88E-11
Ms4a6d	ENSMUSG00000024679	1.717321555	6.02E-11
Bcl2l1	ENSMUSG00000007659	1.250873194	6.19E-11
Aox1	ENSMUSG00000063558	-1.722306748	6.48E-11
Gm28439	ENSMUSG00000100131	-2.021007853	6.80E-11
Bckdha	ENSMUSG00000060376	-1.360256382	7.30E-11
Fscn1	ENSMUSG00000029581	1.379792852	7.63E-11
Ldhd	ENSMUSG00000031958	-1.697276695	8.87E-11
Dynll1	ENSMUSG00000009013	1.497906438	8.90E-11
Dnajc28	ENSMUSG00000039763	-2.150473343	9.00E-11
Idh2	ENSMUSG00000030541	-1.031044299	9.59E-11
Arpc1b	ENSMUSG00000029622	1.312870759	9.71E-11
Coro1a	ENSMUSG00000030707	1.490835437	9.78E-11
Clec11a	ENSMUSG00000004473	2.389629889	9.90E-11
Anxa1	ENSMUSG00000024659	1.346395218	1.00E-10
Top2a	ENSMUSG00000020914	2.42896681	1.11E-10

Pcnt	ENSMUSG00000001151	-1.650316338	1.28E-10
Pmepa1	ENSMUSG00000038400	1.362409176	1.56E-10
Fbp2	ENSMUSG00000021456	-1.635664176	1.56E-10
E130119H09Rik	ENSMUSG00000114069	-2.892968403	1.72E-10
Lmna	ENSMUSG00000028063	1.103847599	1.79E-10
Atf4	ENSMUSG00000042406	1.320701471	1.80E-10
Hsp90aa1	ENSMUSG00000021270	1.50804298	2.17E-10
Vmn2r3	ENSMUSG00000091572	8.562155894	2.29E-10
Rpl3-ps1	ENSMUSG00000084349	1.439354076	2.43E-10
Lman1l	ENSMUSG00000056271	3.551987207	2.74E-10
Pgam1	ENSMUSG00000011752	1.131154962	2.81E-10
Slc25a42	ENSMUSG00000002346	-1.785975429	2.86E-10
Gja1	ENSMUSG00000050953	-1.141270737	2.90E-10
Csrp1	ENSMUSG00000026421	1.183193617	2.93E-10
Foxs1	ENSMUSG00000074676	2.391006048	3.10E-10
Aldh6a1	ENSMUSG00000021238	-1.534658196	3.11E-10
Cars	ENSMUSG00000010755	1.288183132	3.98E-10
Hectd2os	ENSMUSG00000087579	2.006538743	4.18E-10
Tceal9	ENSMUSG00000042712	1.24380372	4.24E-10
Tpm3	ENSMUSG00000027940	1.114507058	4.24E-10
Dglucy	ENSMUSG00000021185	-1.626080723	4.49E-10
Acy3	ENSMUSG00000024866	-1.678974192	4.66E-10
Rhot2	ENSMUSG00000025733	-1.374946825	4.74E-10
Lgals1	ENSMUSG00000068220	1.179819189	5.99E-10
Ms4a6b	ENSMUSG00000024677	1.330928388	6.24E-10
Adrb1	ENSMUSG00000035283	-1.848200754	6.41E-10
Mylk3	ENSMUSG00000031698	-1.206414445	6.46E-10
Stk17b	ENSMUSG00000026094	1.231727437	6.54E-10
Ptma	ENSMUSG00000026238	1.059842323	7.81E-10
Gstm7	ENSMUSG00000004035	-1.501157336	7.93E-10
Eno1	ENSMUSG00000063524	1.364874044	7.98E-10
Cd63	ENSMUSG00000025351	1.155694029	8.85E-10
Stbd1	ENSMUSG00000047963	1.330713696	9.14E-10
Ifrd1	ENSMUSG00000001627	1.443263483	9.26E-10
Panx1	ENSMUSG00000031934	2.328782818	9.37E-10
Pln	ENSMUSG00000038583	-1.458916395	9.52E-10
Pmm1	ENSMUSG00000022474	1.208562023	9.78E-10
Txlnb	ENSMUSG00000039891	-1.225724861	1.03E-09
Thbs4	ENSMUSG00000021702	3.720445709	1.04E-09
Ccdc141	ENSMUSG00000044033	-1.183646806	1.04E-09

Col26a1	ENSMUSG00000004415	4.072984991	1.06E-09
Abca4	ENSMUSG00000028125	-2.775181654	1.27E-09
Ccna2	ENSMUSG00000027715	2.715828155	1.30E-09
Alpl	ENSMUSG00000028766	1.204343501	1.32E-09
Ctss	ENSMUSG00000038642	1.48431674	1.33E-09
Svep1	ENSMUSG00000028369	1.608693007	1.34E-09
Odc1	ENSMUSG00000011179	1.114872325	1.36E-09
Suclg2	ENSMUSG00000061838	-1.160499725	1.43E-09
Etv4	ENSMUSG00000017724	3.292094387	1.47E-09
Lrrc15	ENSMUSG00000052316	-2.683873502	1.48E-09
Tbc1d10c	ENSMUSG00000040247	-1.980853139	1.55E-09
Zdhhc20	ENSMUSG00000021969	1.24985723	1.55E-09
H2-Ab1	ENSMUSG00000073421	1.296778439	1.55E-09
Ereg	ENSMUSG00000029377	4.507934759	1.64E-09
Mov10l1	ENSMUSG00000015365	-1.157687184	1.78E-09
Cytip	ENSMUSG00000026832	3.078030454	2.00E-09
Clec3b	ENSMUSG00000025784	-1.183196426	2.01E-09
Inppl1	ENSMUSG00000032737	-1.262356641	2.08E-09
Aldh4a1	ENSMUSG00000028737	-1.49628212	2.31E-09
Fgf16	ENSMUSG00000031230	-1.316309439	2.31E-09
mt-Cytb	ENSMUSG00000064370	-1.299085481	2.31E-09
Tacc3	ENSMUSG00000037313	2.3693115	2.35E-09
Slc25a34	ENSMUSG00000040740	-1.045875463	2.41E-09
Rbp7	ENSMUSG00000028996	1.446047092	2.44E-09
Uckl1os	ENSMUSG00000010492	-4.20075733	2.49E-09
Whrn	ENSMUSG00000039137	-2.192637354	2.87E-09
Mxd3	ENSMUSG00000021485	3.371228492	2.93E-09
Nrep	ENSMUSG00000042834	1.292874815	3.55E-09
Mrc2	ENSMUSG00000020695	1.272922686	3.55E-09
Gm24474	ENSMUSG00000114005	-2.151162038	3.59E-09
Hmgb2	ENSMUSG00000054717	1.689671639	3.62E-09
Cmbl	ENSMUSG00000022235	-1.570267925	3.64E-09
Picalm	ENSMUSG00000039361	1.111707272	3.92E-09
Kctd17	ENSMUSG00000033287	1.225876862	4.47E-09
Lsmem1	ENSMUSG00000071342	-1.471534295	4.49E-09
Hmmr	ENSMUSG00000020330	4.463823733	4.85E-09
Sh3bgrl	ENSMUSG00000031246	1.048945304	4.97E-09
Medag	ENSMUSG00000029659	1.355197052	5.05E-09
Knstrn	ENSMUSG00000027331	2.548632622	5.12E-09
As3mt	ENSMUSG00000003559	-1.899692704	5.17E-09

Mafk	ENSMUSG00000018143	1.210680889	5.20E-09
Kcnj11	ENSMUSG00000096146	-1.187051512	5.28E-09
Penk	ENSMUSG00000045573	-2.326313576	5.54E-09
Klf6	ENSMUSG00000000078	1.126536986	5.64E-09
Ckap2	ENSMUSG00000037725	3.021135264	6.10E-09
Rpl10-ps3	ENSMUSG00000058443	1.510131781	6.28E-09
Cpeb3	ENSMUSG00000039652	-1.423853504	6.46E-09
Aldh18a1	ENSMUSG00000025007	1.548098866	6.96E-09
Kcne4	ENSMUSG00000047330	2.278068027	7.09E-09
Ptx3	ENSMUSG00000027832	2.805453152	7.25E-09
Pabpc1	ENSMUSG00000022283	1.095645568	8.42E-09
Cdc42ep3	ENSMUSG00000036533	-1.225764343	8.43E-09
Slc38a2	ENSMUSG00000022462	1.08494162	8.75E-09
Atp2a2	ENSMUSG00000029467	-1.481329604	9.19E-09
Arf6	ENSMUSG00000044147	1.054991361	9.35E-09
Spry1	ENSMUSG00000037211	1.397059289	1.04E-08
Litaf	ENSMUSG00000022500	1.159661437	1.09E-08
Map3k20	ENSMUSG00000004085	-1.143859176	1.11E-08
Actb	ENSMUSG00000029580	1.013530866	1.18E-08
Gm20619	ENSMUSG00000093482	-2.227015677	1.19E-08
Klhdc1	ENSMUSG00000051890	-1.597766512	1.19E-08
P4ha1	ENSMUSG00000019916	1.046419732	1.24E-08
Gm34302	ENSMUSG00000103476	-2.849269596	1.26E-08
S100a16	ENSMUSG00000074457	1.085701783	1.32E-08
Nars	ENSMUSG00000024587	1.022651249	1.39E-08
Cfl1	ENSMUSG00000056201	1.152143909	1.55E-08
Pls3	ENSMUSG00000016382	1.086169461	1.67E-08
Nr1d1	ENSMUSG00000020889	-1.105072754	1.74E-08
Corin	ENSMUSG00000005220	-1.239134533	1.75E-08
Hadh	ENSMUSG00000027984	-1.079810795	1.75E-08
Ccnb1	ENSMUSG00000041431	3.599071559	1.77E-08
Ngef	ENSMUSG00000026259	3.208663189	1.78E-08
Racgap1	ENSMUSG00000023015	2.198902117	1.78E-08
Yars	ENSMUSG00000028811	1.141417972	1.87E-08
Scara5	ENSMUSG00000022032	-1.436955593	1.87E-08
Actr3	ENSMUSG00000026341	0.971425329	1.98E-08
Mcm6	ENSMUSG00000026355	1.450775708	1.99E-08
Clic1	ENSMUSG00000007041	1.114294066	1.99E-08
Mcam	ENSMUSG00000032135	1.370916367	2.03E-08
Cryba4	ENSMUSG00000066975	-1.808966818	2.17E-08

Cacng6	ENSMUSG00000078815	-4.315988295	2.26E-08
Osbp2	ENSMUSG00000020435	-1.886620724	2.32E-08
Pdk2	ENSMUSG00000038967	-1.069364128	2.34E-08
Prss23	ENSMUSG00000039405	1.607192494	2.81E-08
Ect2	ENSMUSG00000027699	2.635812302	2.81E-08
Col9a2	ENSMUSG00000028626	4.199147129	2.81E-08
Tmem143	ENSMUSG00000002781	-1.156884213	2.85E-08
Selenof	ENSMUSG00000037072	1.044381895	2.85E-08
Ppp1r14c	ENSMUSG00000040653	-1.109288297	2.89E-08
Ptpn3	ENSMUSG00000038764	-1.36366362	2.90E-08
2610524H06Rik	ENSMUSG00000092486	2.4704626	2.92E-08
Rnd3	ENSMUSG00000017144	1.368306832	3.00E-08
Anxa5	ENSMUSG00000027712	0.991420339	3.02E-08
Asb15	ENSMUSG00000029685	-1.749238112	3.08E-08
Rap1b	ENSMUSG00000052681	1.001536103	3.27E-08
Kdelr2	ENSMUSG00000079111	1.124684615	3.54E-08
Jpt1	ENSMUSG00000020737	1.152226579	3.64E-08
Atcayos	ENSMUSG00000085779	-1.428870514	3.66E-08
Gja3	ENSMUSG00000048582	-1.450538838	3.66E-08
Coq9	ENSMUSG00000031782	-1.007582523	3.72E-08
Tfpi	ENSMUSG00000027082	-1.031681412	3.82E-08
Ada	ENSMUSG00000017697	2.577398542	3.85E-08
mt-Atp6	ENSMUSG00000064357	-2.821679402	4.08E-08
BC028528	ENSMUSG00000038543	1.617634083	4.26E-08
Pgk1	ENSMUSG00000062070	1.174263813	4.44E-08
Gm10635	ENSMUSG00000111765	-3.675888905	4.45E-08
A430046D13Rik	ENSMUSG00000097353	-1.408239942	4.51E-08
Eif6	ENSMUSG00000027613	1.03563277	4.77E-08
Rai14	ENSMUSG00000022246	1.598287861	4.78E-08
Mdga1	ENSMUSG00000043557	-2.018188985	4.82E-08
Hopx	ENSMUSG00000059325	-1.302793327	4.91E-08
Tppp	ENSMUSG00000021573	-1.335925854	4.99E-08
Slc2a1	ENSMUSG00000028645	1.394838603	5.32E-08
Prc1	ENSMUSG00000038943	2.972217734	5.32E-08
Bcl2a1b	ENSMUSG00000089929	3.409056498	5.59E-08
Pm20d1	ENSMUSG00000042251	-2.822383445	5.61E-08
Rbp1	ENSMUSG00000046402	1.643889764	5.76E-08
Carhsp1	ENSMUSG00000008393	1.106128905	5.98E-08
4930438A08Rik	ENSMUSG00000069873	7.917579484	6.18E-08
D2hgdh	ENSMUSG00000073609	-1.379295888	6.22E-08

Enpp1	ENSMUSG00000037370	1.726484621	6.22E-08
Ift81	ENSMUSG00000029469	-1.585152995	6.25E-08
Mthfd1l	ENSMUSG00000040675	1.915872777	6.31E-08
Gm43672	ENSMUSG00000106019	-1.302893763	6.53E-08
Slc7a5	ENSMUSG00000040010	2.170275436	6.62E-08
Dsg2	ENSMUSG00000044393	-1.476810937	7.16E-08
Mical2	ENSMUSG00000038244	1.427386086	7.18E-08
Crhr2	ENSMUSG00000003476	-1.648720925	7.40E-08
Adamts8	ENSMUSG00000031994	3.146970039	7.59E-08
Nedd9	ENSMUSG00000021365	1.411472794	7.97E-08
Depp1	ENSMUSG00000048489	1.36330161	8.24E-08
Dbt	ENSMUSG00000000340	-1.39264822	8.52E-08
Efcab2	ENSMUSG00000026495	-1.212947965	8.70E-08
Acot9	ENSMUSG00000025287	1.079173621	9.32E-08
Prr12	ENSMUSG00000046574	-1.675536106	9.37E-08
Rpsa-ps10	ENSMUSG00000047676	1.013317852	9.57E-08
Echdc3	ENSMUSG00000039063	-1.427303502	9.57E-08
Tubb4b	ENSMUSG00000036752	0.905436241	9.58E-08
Dok1	ENSMUSG00000068335	2.006759223	9.70E-08
Lrrc3b	ENSMUSG00000045201	-1.396099229	9.80E-08
Gm10222	ENSMUSG00000067736	-2.479558908	9.89E-08
Cyb561	ENSMUSG00000019590	1.246596113	9.89E-08
Hnmt	ENSMUSG00000026986	-1.570188875	1.00E-07
Jmjd6	ENSMUSG00000056962	1.00991841	1.03E-07
Ddx39	ENSMUSG00000005481	1.088900805	1.03E-07
Kcnip2	ENSMUSG00000025221	-1.698980043	1.04E-07
Mki67	ENSMUSG00000031004	1.850086628	1.11E-07
Etl4	ENSMUSG00000036617	-1.414607854	1.12E-07
Ccnb2	ENSMUSG00000032218	3.045717899	1.12E-07
Vim	ENSMUSG00000026728	1.117022987	1.13E-07
H2-Eb1	ENSMUSG00000060586	1.252099092	1.16E-07
Srsf3	ENSMUSG00000071172	0.98874174	1.18E-07
Calm1	ENSMUSG00000001175	0.878217807	1.22E-07
Ifi211	ENSMUSG00000026536	1.36089142	1.23E-07
Ube2c	ENSMUSG00000001403	2.536036444	1.24E-07
2310039L15Rik	ENSMUSG00000100550	-2.201106114	1.24E-07
Tnfrsf11b	ENSMUSG00000063727	4.233880394	1.25E-07
Npr3	ENSMUSG00000022206	-1.056897283	1.25E-07
Cbx7	ENSMUSG00000053411	-1.27869521	1.28E-07
Prkaca	ENSMUSG00000005469	-0.893072595	1.29E-07

Slfn2	ENSMUSG00000072620	1.20626714	1.29E-07
Rap1gap2	ENSMUSG00000038807	-1.177816284	1.31E-07
Enpp2	ENSMUSG00000022425	-1.632158518	1.31E-07
Ckap4	ENSMUSG00000046841	1.105612636	1.34E-07
Gstk1	ENSMUSG00000029864	-1.636796726	1.38E-07
Nfil3	ENSMUSG00000056749	1.432258298	1.41E-07
Yipf5	ENSMUSG00000024487	1.120900942	1.42E-07
Upk3b	ENSMUSG00000042985	-1.225821045	1.46E-07
Asb5	ENSMUSG00000031519	-1.231939772	1.47E-07
Igfbp7	ENSMUSG00000036256	1.450098108	1.49E-07
Aldh1a3	ENSMUSG00000015134	2.259025568	1.56E-07
Cald1	ENSMUSG00000029761	1.05983205	1.56E-07
Gm10275	ENSMUSG00000069682	1.2406467	1.56E-07
Marcks	ENSMUSG00000069662	0.975172509	1.68E-07
Actg1	ENSMUSG00000062825	0.933311914	1.68E-07
Rpl35a-ps2	ENSMUSG00000081214	1.458857744	1.68E-07
Morf4l2	ENSMUSG00000031422	0.939707691	1.79E-07
Cgnl1	ENSMUSG00000032232	-0.952450327	1.95E-07
Clasp1	ENSMUSG00000064302	-1.218235895	1.95E-07
Frmpd3	ENSMUSG00000042425	-2.464777328	1.96E-07
S100a6	ENSMUSG00000001025	1.370682787	2.04E-07
Chac1	ENSMUSG00000027313	1.818388023	2.08E-07
Aldh1a2	ENSMUSG00000013584	1.408554456	2.11E-07
Ank2	ENSMUSG00000032826	-1.164278048	2.24E-07
Wnk2	ENSMUSG00000037989	-1.662404213	2.27E-07
Acss1	ENSMUSG00000027452	-0.94780131	2.32E-07
Maob	ENSMUSG00000040147	-1.494524853	2.40E-07
Myc	ENSMUSG00000022346	1.655143888	2.42E-07
Tpd52l1	ENSMUSG00000000296	-1.682348722	2.42E-07
Fbln2	ENSMUSG00000064080	1.255221016	2.42E-07
Otulin	ENSMUSG00000046034	1.076945673	2.50E-07
P2ry10	ENSMUSG00000050921	4.732145436	2.52E-07
Uros	ENSMUSG00000030979	-1.177252667	2.78E-07
Rpl10a-ps1	ENSMUSG00000084416	1.191344246	2.79E-07
Adcy6	ENSMUSG00000022994	-0.986761727	2.99E-07
Slc9a2	ENSMUSG00000026062	-2.014313559	3.02E-07
Kyat3	ENSMUSG00000040213	-1.461158055	3.04E-07
Prrc2b	ENSMUSG00000039262	-1.146163653	3.08E-07
Tmem35a	ENSMUSG00000033578	-2.08833436	3.14E-07
Agtr1a	ENSMUSG00000049115	-1.699078418	3.22E-07

Cenpm	ENSMUSG00000068101	2.719405376	3.24E-07
Arpc5	ENSMUSG00000008475	0.943985405	3.25E-07
Tubb3	ENSMUSG00000062380	2.0086526	3.33E-07
Krt222	ENSMUSG00000035849	-1.371035574	3.33E-07
Birc5	ENSMUSG00000017716	2.698983358	3.33E-07
Plin3	ENSMUSG00000024197	-0.976503999	3.41E-07
Tcea3	ENSMUSG00000001604	-1.067527318	3.45E-07
Rps12-ps3	ENSMUSG00000067038	0.918026342	3.53E-07
Clec4d	ENSMUSG00000030144	2.362230626	3.57E-07
Klrd1	ENSMUSG00000030165	4.637941588	3.58E-07
D3Ertd751e	ENSMUSG00000025766	-1.389297011	3.62E-07
Pkhd1l1	ENSMUSG00000038725	-1.836616059	3.65E-07
Nfkbia	ENSMUSG00000021025	0.933653559	3.67E-07
Cd52	ENSMUSG00000000682	1.63222227	3.72E-07
B230369F24Rik	ENSMUSG00000086350	-1.803618893	3.77E-07
Gm28661	ENSMUSG00000102070	-1.444872074	3.81E-07
Bcat1	ENSMUSG00000030268	1.96361542	3.81E-07
Tgfb3	ENSMUSG00000021253	1.201545313	3.81E-07
Mut	ENSMUSG00000023921	-1.232699487	3.81E-07
Nqo2	ENSMUSG00000046949	-1.111149875	3.89E-07
Pfkl	ENSMUSG00000020277	0.853857919	3.89E-07
S1pr1	ENSMUSG00000045092	1.155256917	3.92E-07
Loxl3	ENSMUSG00000000693	1.782966926	3.92E-07
Heatr5b	ENSMUSG00000039414	-1.326305651	4.05E-07
Dbf4	ENSMUSG00000002297	2.245377208	4.07E-07
mt-Nd4	ENSMUSG00000064363	-1.31561741	4.20E-07
Gm19410	ENSMUSG00000109372	2.353395442	4.22E-07
H2afz	ENSMUSG00000037894	1.05836761	4.27E-07
Nrip3	ENSMUSG00000034825	7.353270702	4.34E-07
Ackr4	ENSMUSG00000079355	1.336284833	4.40E-07
Depdc1a	ENSMUSG00000028175	3.621837645	4.40E-07
Ryr2	ENSMUSG00000021313	-1.216800211	4.46E-07
Gins2	ENSMUSG00000031821	1.939140236	4.57E-07
Pfkfb1	ENSMUSG00000025271	-2.33875793	4.68E-07
Hprt	ENSMUSG00000025630	0.853570528	4.73E-07
Irs2	ENSMUSG00000038894	1.641698169	4.76E-07
Gm13340	ENSMUSG00000083563	-1.662793366	4.76E-07
Eno1b	ENSMUSG00000059040	2.069814265	4.76E-07
Ccr2	ENSMUSG00000049103	1.594619683	4.76E-07
Sla	ENSMUSG00000022372	1.582972085	4.80E-07

Calcoco1	ENSMUSG00000023055	-1.020374293	4.84E-07
Ric8b	ENSMUSG00000035620	-1.267871909	4.86E-07
Sfrp2	ENSMUSG00000027996	1.629466118	4.92E-07
Fmo5	ENSMUSG00000028088	-1.249585722	4.92E-07
Nipsnap2	ENSMUSG00000029432	-0.908409251	5.00E-07
Scml4	ENSMUSG00000044770	2.079966317	5.06E-07
Etfbkmt	ENSMUSG00000039958	-1.433490273	5.20E-07
Eif1a	ENSMUSG00000057561	1.008395935	5.22E-07
Fam198b	ENSMUSG00000027955	1.004972003	5.24E-07
Hlf	ENSMUSG00000003949	-1.467583353	5.36E-07
Cthrc1	ENSMUSG00000054196	4.509550324	5.89E-07
Abhd18	ENSMUSG00000037818	-1.211143129	5.92E-07
Ttc38	ENSMUSG00000035944	-1.39290838	5.92E-07
Stom	ENSMUSG00000026880	-1.108731679	5.94E-07
Tango2	ENSMUSG00000013539	-1.156761062	5.95E-07
Pdcd10	ENSMUSG00000027835	0.986864941	6.16E-07
Dhrs7c	ENSMUSG00000033044	-1.397352932	6.22E-07
1600014C23Rik	ENSMUSG00000094690	3.78417042	6.33E-07
Kcnh2	ENSMUSG00000038319	-1.179884022	6.34E-07
Hspa1b	ENSMUSG00000090877	2.424431588	6.56E-07
Diaph3	ENSMUSG00000022021	2.688551453	6.65E-07
Fdft1	ENSMUSG00000021273	-1.156392912	6.69E-07
Mettl7a1	ENSMUSG00000054619	-1.141907495	6.72E-07
Rgs1	ENSMUSG00000026358	4.834812859	6.74E-07
Ctla2a	ENSMUSG00000044258	1.328038817	6.81E-07
Fhod3	ENSMUSG00000034295	-0.952770546	6.81E-07
Gm48607	ENSMUSG00000113735	-3.444222274	6.81E-07
Inpp5e	ENSMUSG00000026925	-1.431555533	6.81E-07
Lyrm9	ENSMUSG00000072640	-1.352065843	6.92E-07
Ldha-ps2	ENSMUSG00000083836	2.222587439	6.92E-07
Cdr2	ENSMUSG00000030878	1.353940296	7.25E-07
Vgll3	ENSMUSG00000091243	1.493175362	7.50E-07
Plekha4	ENSMUSG00000040428	1.58712065	7.51E-07
Ms4a4a	ENSMUSG00000101389	1.382556659	7.63E-07
Ppic	ENSMUSG00000024538	1.261349271	7.65E-07
Smim20	ENSMUSG00000061461	-1.028939027	7.73E-07
mt-Tl2	ENSMUSG00000064366	-3.681813462	7.97E-07
Tagln	ENSMUSG00000032085	3.654760516	7.98E-07
Poln	ENSMUSG00000045102	-2.595660052	8.01E-07
Emid1	ENSMUSG00000034164	2.16552562	8.04E-07

Gpx7	ENSMUSG00000028597	1.244044647	8.07E-07
Mfap2	ENSMUSG00000060572	1.602762683	8.20E-07
Nhp2	ENSMUSG00000001056	1.144406206	8.27E-07
Avil	ENSMUSG00000025432	3.521076359	8.44E-07
Ttll1	ENSMUSG00000022442	-1.653123847	8.65E-07
Klhl30	ENSMUSG00000026308	-1.2986904	8.77E-07
Lhfp	ENSMUSG00000048332	1.084873348	8.85E-07
Mt2	ENSMUSG00000031762	2.34454108	8.86E-07
Adamts2	ENSMUSG00000036545	1.241192914	8.93E-07
Tmem119	ENSMUSG00000054675	1.133402979	9.09E-07
Abcb8	ENSMUSG00000028973	-0.973710635	9.09E-07
Adcy5	ENSMUSG00000022840	-1.232697742	9.09E-07
Cacna1c	ENSMUSG00000051331	-1.117413652	9.13E-07
Irx1	ENSMUSG00000060969	-1.846254366	9.14E-07
Rps27a	ENSMUSG00000020460	1.289587733	9.34E-07
Armh3	ENSMUSG00000039901	-1.26768931	9.55E-07
2310040G24Rik	ENSMUSG00000101655	-1.526497941	9.65E-07
Kcnn2	ENSMUSG00000054477	-1.880933328	1.01E-06
Gchfr	ENSMUSG00000046814	-1.765790796	1.04E-06
Thy1	ENSMUSG00000032011	1.173011758	1.06E-06
Cenpk	ENSMUSG00000021714	3.589882965	1.07E-06
Rpsa	ENSMUSG00000032518	0.849168127	1.08E-06
Ckap2l	ENSMUSG00000048327	2.540091029	1.09E-06
Sall1	ENSMUSG00000031665	-2.215722309	1.11E-06
Gm28979	ENSMUSG00000101941	-2.812744898	1.11E-06
Mest	ENSMUSG00000051855	1.846238019	1.11E-06
Rpl19-ps11	ENSMUSG00000081094	1.266684863	1.11E-06
Slc41a1	ENSMUSG00000013275	-1.111438093	1.12E-06
Cavin3	ENSMUSG00000037060	1.163648813	1.12E-06
Aldh2	ENSMUSG00000029455	-0.903893704	1.14E-06
Tspan6	ENSMUSG00000067377	1.250966435	1.15E-06
Gypc	ENSMUSG00000090523	-1.074601061	1.17E-06
Gcdh	ENSMUSG00000003809	-1.18181064	1.18E-06
Pdlim2	ENSMUSG00000022090	1.351899297	1.20E-06
Isoc2a	ENSMUSG00000086784	-1.288442646	1.23E-06
Acadm	ENSMUSG00000062908	-1.071899692	1.24E-06
Etfdh	ENSMUSG00000027809	-0.959155245	1.25E-06
Mmp12	ENSMUSG00000049723	2.816258461	1.26E-06
Ran	ENSMUSG00000029430	0.807309196	1.26E-06
Pimreg	ENSMUSG00000020808	2.817618727	1.26E-06

Nppb	ENSMUSG00000029019	3.396223974	1.29E-06
Vwf	ENSMUSG00000001930	-1.03329801	1.29E-06
Ehhadh	ENSMUSG00000022853	-1.655172524	1.29E-06
Mpeg1	ENSMUSG00000046805	1.607237249	1.31E-06
Eef1a1	ENSMUSG00000037742	1.076272601	1.35E-06
Kbtbd3	ENSMUSG00000025893	-1.578508105	1.36E-06
BB218582	ENSMUSG00000085218	-2.128366264	1.39E-06
Gpr153	ENSMUSG00000042804	1.353800671	1.39E-06
Prrx2	ENSMUSG00000039476	2.916719161	1.41E-06
Angptl4	ENSMUSG00000002289	1.669918231	1.41E-06
Col5a1	ENSMUSG00000026837	1.049856466	1.45E-06
Endod1	ENSMUSG00000037419	1.159387638	1.45E-06
Arid5a	ENSMUSG00000037447	1.468441446	1.47E-06
Ostc	ENSMUSG00000041084	0.873435461	1.48E-06
Golt1b	ENSMUSG00000030245	1.102086299	1.49E-06
Tmem108	ENSMUSG00000042757	-1.435328394	1.51E-06
Cacnb1	ENSMUSG00000020882	1.908865821	1.57E-06
Col6a2	ENSMUSG00000020241	0.905451999	1.59E-06
Vit	ENSMUSG00000024076	-1.494954843	1.63E-06
1700017B05Rik	ENSMUSG00000032300	1.358026403	1.64E-06
6030408B16Rik	ENSMUSG00000075408	1.56702257	1.66E-06
Capza1	ENSMUSG00000070372	0.973969225	1.67E-06
Tmtc1	ENSMUSG00000030306	-0.907236757	1.67E-06
Slc16a3	ENSMUSG00000025161	1.563490171	1.72E-06
Tcaim	ENSMUSG00000046603	-1.396982059	1.72E-06
Lsp1	ENSMUSG00000018819	1.125766833	1.72E-06
Rpl13a	ENSMUSG00000074129	1.040763054	1.82E-06
Fxyd5	ENSMUSG00000009687	1.280068442	1.91E-06
Rps14	ENSMUSG00000024608	1.018122514	1.94E-06
Eif2s2	ENSMUSG00000074656	0.934743123	1.96E-06
Higd1b	ENSMUSG00000020928	1.314240108	1.99E-06
Nav2	ENSMUSG00000052512	-1.618533414	2.06E-06
Mturn	ENSMUSG00000038065	-1.059528786	2.13E-06
Msrb2	ENSMUSG00000023094	-1.283088691	2.14E-06
Fibin	ENSMUSG00000074971	1.444836961	2.15E-06
Kcnj5	ENSMUSG00000032034	-1.473086748	2.16E-06
Cnn3	ENSMUSG00000053931	0.953213013	2.16E-06
Acacb	ENSMUSG00000042010	-1.146300725	2.16E-06
Gramd1b	ENSMUSG00000040111	-1.261007214	2.16E-06
Efhd2	ENSMUSG00000040659	1.288512251	2.16E-06

Myl6	ENSMUSG00000090841	1.234969997	2.18E-06
Arrdc4	ENSMUSG00000042659	1.446775884	2.21E-06
Ppp4c	ENSMUSG00000030697	0.88336881	2.27E-06
Pcca	ENSMUSG00000041650	-1.005099429	2.29E-06
Gm7901	ENSMUSG00000101431	2.991966058	2.31E-06
Tmem158	ENSMUSG00000054871	1.461832649	2.32E-06
Ncf1	ENSMUSG00000015950	1.108205441	2.33E-06
Gm6793	ENSMUSG00000092086	1.105569248	2.34E-06
Ms4a6c	ENSMUSG00000079419	1.348001262	2.35E-06
Hsbp1l1	ENSMUSG00000078963	-2.351168081	2.37E-06
Slc1a3	ENSMUSG00000005360	1.709358949	2.47E-06
Gm43050	ENSMUSG00000106795	-2.176600341	2.50E-06
Ccdc69	ENSMUSG00000049588	-1.710677001	2.53E-06
Plac8	ENSMUSG00000029322	1.664372936	2.53E-06
Serpinh1	ENSMUSG00000070436	0.91056999	2.53E-06
Ipo13	ENSMUSG00000033365	-1.066094799	2.54E-06
Ms4a4c	ENSMUSG00000024675	2.254168523	2.56E-06
S100a11	ENSMUSG00000027907	1.195277531	2.58E-06
Angptl6	ENSMUSG00000038742	1.435140913	2.61E-06
Gpd1	ENSMUSG00000023019	-0.974235267	2.68E-06
Prdx4	ENSMUSG00000025289	0.92243383	2.69E-06
Rps6ka2	ENSMUSG00000023809	-1.31394641	2.73E-06
Praf2	ENSMUSG00000031149	1.440734502	2.73E-06
Iigp1	ENSMUSG00000054072	-1.072981009	2.75E-06
Ltbp2	ENSMUSG00000002020	4.140731884	2.79E-06
Lhfpl2	ENSMUSG00000045312	1.560997217	2.83E-06
Cnksr1	ENSMUSG00000028841	1.499789335	2.83E-06
Gtf2i	ENSMUSG00000060261	-0.851680444	2.87E-06
Nebl	ENSMUSG00000053702	-1.115171316	2.93E-06
Cenpi	ENSMUSG00000031262	3.219661164	2.94E-06
Gpx8	ENSMUSG00000021760	0.947470771	3.00E-06
Hdac11	ENSMUSG00000034245	-1.33163119	3.00E-06
Pkia	ENSMUSG00000027499	-1.080577589	3.07E-06
Prpf38a	ENSMUSG00000063800	0.948649548	3.11E-06
Pik3ip1	ENSMUSG00000034614	-1.087365344	3.13E-06
Gstm2	ENSMUSG00000040562	-0.964434295	3.13E-06
Efemp2	ENSMUSG00000024909	0.95613906	3.22E-06
Jdp2	ENSMUSG00000034271	1.197341858	3.30E-06
Klhl24	ENSMUSG00000062901	-0.889560375	3.31E-06
Mitf	ENSMUSG00000035158	-1.176928287	3.40E-06

Utp18	ENSMUSG00000054079	1.128382917	3.41E-06
Mad2l1	ENSMUSG00000029910	1.428997331	3.43E-06
Sap30	ENSMUSG00000031609	1.012894019	3.44E-06
Ehbp1	ENSMUSG00000042302	-0.942440301	3.62E-06
Skil	ENSMUSG00000027660	1.238072039	3.69E-06
Btg2	ENSMUSG00000020423	1.387814323	3.69E-06
Kank1	ENSMUSG00000032702	-0.922280913	3.69E-06
Eepd1	ENSMUSG00000036611	-1.2730314	3.69E-06
Lbh	ENSMUSG00000024063	-0.837310225	3.69E-06
Lsmem2	ENSMUSG00000103409	-1.518770328	3.71E-06
Il4ra	ENSMUSG00000030748	1.416697166	3.73E-06
Gtpbp4	ENSMUSG00000021149	0.97101698	3.77E-06
Tmem94	ENSMUSG00000020747	-1.209690789	3.80E-06
Nr3c2	ENSMUSG00000031618	-1.549845332	3.88E-06
Sfrp1	ENSMUSG00000031548	1.420525603	3.89E-06
Etfa	ENSMUSG00000032314	-0.87917802	3.95E-06
Tmsb4x	ENSMUSG00000049775	1.139017809	3.95E-06
Smim5	ENSMUSG00000048442	-1.396573182	3.98E-06
Csf1	ENSMUSG00000014599	-1.049583458	3.98E-06
Gm30873	ENSMUSG00000109341	2.83354443	4.00E-06
Bub1	ENSMUSG00000027379	2.956984581	4.01E-06
Pak6	ENSMUSG00000074923	-1.527606847	4.07E-06
Cep112	ENSMUSG00000020728	-1.462015874	4.08E-06
Ppp1r15a	ENSMUSG00000040435	1.179358054	4.13E-06
Unc5b	ENSMUSG00000020099	1.332599365	4.18E-06
Xpnpep2	ENSMUSG00000037005	-2.634116217	4.20E-06
Lrrn4	ENSMUSG00000043110	-1.749282966	4.23E-06
Rps13-ps2	ENSMUSG00000069972	1.150089587	4.38E-06
Gpm6b	ENSMUSG00000031342	0.899424449	4.43E-06
Gm48583	ENSMUSG00000114277	1.610156518	4.43E-06
Angpt1	ENSMUSG00000022309	-1.21211191	4.45E-06
Eva1b	ENSMUSG00000050212	1.177838775	4.46E-06
Pcsk6	ENSMUSG00000030513	-0.989261561	4.52E-06
Cs	ENSMUSG00000005683	-0.901349725	4.57E-06
Pitpnc1	ENSMUSG00000040430	-1.076536474	4.71E-06
Sox7	ENSMUSG00000063060	-1.440691502	4.80E-06
Pck2	ENSMUSG00000040618	1.020324049	4.83E-06
Rps18	ENSMUSG00000008668	0.956038813	4.85E-06
Scn4a	ENSMUSG00000001027	-2.579729725	4.87E-06
Tpi1	ENSMUSG00000023456	0.764152283	4.89E-06

Esd	ENSMUSG00000021996	0.950015559	4.91E-06
Cep128	ENSMUSG00000061533	-1.467425753	4.93E-06
Trem2	ENSMUSG00000023992	1.59614106	4.97E-06
Svil	ENSMUSG00000024236	-0.980200696	4.97E-06
D830032E09Rik	ENSMUSG00000100457	-1.73556853	4.97E-06
Gm37829	ENSMUSG00000104453	-1.332341099	5.07E-06
Gfra1	ENSMUSG00000025089	-1.632863435	5.19E-06
Tspan9	ENSMUSG00000030352	0.90529151	5.23E-06
Mrpl42	ENSMUSG00000062981	-0.803593259	5.23E-06
Amy1	ENSMUSG00000074264	-2.139902006	5.33E-06
Mettl1	ENSMUSG00000006732	1.214694556	5.63E-06
Tmod3	ENSMUSG00000058587	0.876173709	5.64E-06
4930481A15Rik	ENSMUSG00000086938	-1.610341823	5.69E-06
Wfikkn2	ENSMUSG00000044177	-1.918438656	5.75E-06
Prepl	ENSMUSG00000024127	-1.098835129	5.77E-06
Ralgapa2	ENSMUSG00000037110	-1.101631436	6.04E-06
Pxmp2	ENSMUSG00000029499	-1.062813203	6.04E-06
Rab31	ENSMUSG00000056515	1.332180692	6.07E-06
Sdf2l1	ENSMUSG00000022769	1.212363324	6.08E-06
Kif11	ENSMUSG00000012443	2.065763225	6.09E-06
Cdca3	ENSMUSG00000023505	2.379480007	6.11E-06
Gnl3	ENSMUSG00000042354	0.926123797	6.18E-06
Pbp2	ENSMUSG00000047104	7.072008991	6.18E-06
Stc1	ENSMUSG00000014813	1.73235579	6.19E-06
Sbk2	ENSMUSG00000030433	-2.150162998	6.27E-06
Qsox1	ENSMUSG00000033684	0.940451241	6.77E-06
Tnfsf18	ENSMUSG00000066755	3.989097012	6.81E-06
Psmd8	ENSMUSG00000030591	0.892404988	6.96E-06
Snx10	ENSMUSG00000038301	0.939024748	6.96E-06
Shcbp1	ENSMUSG00000022322	2.343715821	7.01E-06
Alox5	ENSMUSG00000025701	-1.106249609	7.01E-06
Vwa8	ENSMUSG00000058997	-0.973577962	7.01E-06
Ncapg2	ENSMUSG00000042029	1.836343999	7.06E-06
Slc25a29	ENSMUSG00000021265	-1.519509038	7.16E-06
Pla2g5	ENSMUSG00000041193	-1.26396327	7.16E-06
Fcgr4	ENSMUSG00000059089	1.989173394	7.17E-06
Aldh5a1	ENSMUSG00000035936	-1.298072821	7.17E-06
Srxn1	ENSMUSG00000032802	1.042810432	7.40E-06
Serpinf1	ENSMUSG00000000753	1.124034813	7.45E-06
Enah	ENSMUSG00000022995	0.847724523	7.51E-06

Urah	ENSMUSG00000025481	-4.732165114	7.74E-06
Spc25	ENSMUSG00000005233	2.264282555	7.80E-06
Arhgef40	ENSMUSG00000004562	0.930068744	7.89E-06
Fbxo32	ENSMUSG00000022358	-0.892567695	7.93E-06
Rpl17-ps5	ENSMUSG00000081855	1.145788966	7.98E-06
A2m	ENSMUSG00000030111	-2.360770548	8.08E-06
Polq	ENSMUSG00000034206	-2.067809166	8.08E-06
Prox1	ENSMUSG00000010175	-1.182078873	8.13E-06
Ttc9	ENSMUSG00000042734	1.56423955	8.30E-06
Ankrd45	ENSMUSG00000044835	-2.741415049	8.57E-06
Serpinb6a	ENSMUSG00000060147	0.816423095	8.82E-06
Muc16	ENSMUSG00000109564	-2.173683184	8.89E-06
Prr7	ENSMUSG00000034686	2.052441457	8.89E-06
Antxr2	ENSMUSG00000029338	-1.028056568	8.99E-06
AC161607.1	ENSMUSG00000116903	-2.054671129	8.99E-06
Tmem141	ENSMUSG00000026939	-1.091650086	8.99E-06
Tbc1d4	ENSMUSG00000033083	-1.117287141	9.00E-06
Acadsb	ENSMUSG00000030861	-0.880877917	9.24E-06
Mmab	ENSMUSG00000029575	-1.196723219	9.38E-06
Cdkn3	ENSMUSG00000037628	2.379199984	9.48E-06
Arf2	ENSMUSG00000062421	0.883892745	9.52E-06
Mmp23	ENSMUSG00000029061	1.076199741	9.60E-06
Art1	ENSMUSG00000030996	-1.02657637	9.61E-06
Arf4	ENSMUSG00000021877	0.787282653	9.66E-06
Ugp2	ENSMUSG00000001891	1.024933624	9.69E-06
Atf7ip	ENSMUSG00000030213	-0.99017615	9.77E-06
Cdc20	ENSMUSG00000006398	2.016573601	1.00E-05
Ctsz	ENSMUSG00000016256	0.980331627	1.01E-05
Dusp2	ENSMUSG00000027368	2.66872359	1.02E-05
Ncf2	ENSMUSG00000026480	1.151888836	1.03E-05
Ociad2	ENSMUSG00000029153	-1.547386029	1.03E-05
Art5	ENSMUSG00000070424	-1.581138935	1.03E-05
Upf3a	ENSMUSG00000038398	0.96758053	1.03E-05
Arpc4	ENSMUSG00000079426	0.805036541	1.04E-05
Mccc2	ENSMUSG00000021646	-1.028401939	1.06E-05
Map6	ENSMUSG00000055407	1.51042179	1.08E-05
Dbnl	ENSMUSG00000020476	0.849506324	1.11E-05
Kbtbd13	ENSMUSG00000054978	-3.558188052	1.11E-05
Srm	ENSMUSG00000006442	0.905583732	1.11E-05
Fign	ENSMUSG00000075324	-2.31549876	1.13E-05

Rack1	ENSMUSG00000020372	0.767815291	1.14E-05
Smco1	ENSMUSG00000046345	-1.587713255	1.15E-05
Rcn3	ENSMUSG00000019539	1.041754327	1.17E-05
Chrna2	ENSMUSG00000022041	-2.584022339	1.19E-05
Pkd2l2	ENSMUSG00000014503	-1.75548522	1.20E-05
Slamf9	ENSMUSG00000026548	1.113646205	1.20E-05
Zdhhc2	ENSMUSG00000039470	1.372048554	1.21E-05
Ccdc34	ENSMUSG00000027160	1.234342357	1.23E-05
4930453N24Rik	ENSMUSG00000059920	0.84525965	1.24E-05
Ddx50	ENSMUSG00000020076	0.862454248	1.27E-05
Tmbim1	ENSMUSG00000006301	1.19741516	1.28E-05
Tars	ENSMUSG00000022241	0.860727785	1.28E-05
Psmd13	ENSMUSG00000025487	0.786994828	1.30E-05
Gnb3	ENSMUSG00000023439	-1.818104318	1.30E-05
Gm17501	ENSMUSG00000097183	2.09063089	1.30E-05
Pdk4	ENSMUSG00000019577	0.925992244	1.30E-05
Tent5a	ENSMUSG00000032265	1.014447898	1.31E-05
Rgs4	ENSMUSG00000038530	1.150388605	1.32E-05
Hadhb	ENSMUSG00000059447	-0.863533791	1.33E-05
Gbe1	ENSMUSG00000022707	0.88892169	1.33E-05
Psme4	ENSMUSG00000040850	-0.881287277	1.36E-05
Fgf1	ENSMUSG00000036585	-0.84547093	1.36E-05
Ccl5	ENSMUSG00000035042	2.998315146	1.39E-05
Ccdc136	ENSMUSG00000029769	2.02915275	1.40E-05
Sugt1	ENSMUSG00000022024	0.792604883	1.41E-05
Rbm3-ps	ENSMUSG00000099875	2.00498998	1.41E-05
Arhgdib	ENSMUSG00000030220	0.866222892	1.41E-05
Eif4a-ps4	ENSMUSG00000101188	0.948410705	1.42E-05
Akap1	ENSMUSG00000018428	-1.011910312	1.43E-05
Mgst1	ENSMUSG00000008540	-0.823950954	1.46E-05
mt-Atp8	ENSMUSG00000064356	-2.49934843	1.48E-05
Chrm2	ENSMUSG00000045613	-1.228110233	1.49E-05
Mccc1	ENSMUSG00000027709	-1.094579455	1.51E-05
Kcnk2	ENSMUSG00000037624	1.354823517	1.52E-05
Det1	ENSMUSG00000030610	-1.080541949	1.53E-05
Wisp1	ENSMUSG00000005124	2.643785372	1.53E-05
Nsmf	ENSMUSG00000006476	1.145595604	1.55E-05
Angptl7	ENSMUSG00000028989	1.654402865	1.55E-05
Prkab1	ENSMUSG00000029513	-0.996691199	1.56E-05
Col16a1	ENSMUSG00000040690	1.310880065	1.59E-05

Cotl1	ENSMUSG00000031827	1.141160493	1.60E-05
H3f3b	ENSMUSG00000016559	0.834876333	1.62E-05
Npdc1	ENSMUSG00000015094	0.963705353	1.63E-05
Cidea	ENSMUSG00000024526	0.861174926	1.66E-05
Sobp	ENSMUSG00000038248	-1.213106123	1.67E-05
Cacna1s	ENSMUSG00000026407	-1.885799481	1.67E-05
Myh14	ENSMUSG00000030739	-1.097042571	1.71E-05
Lingo3	ENSMUSG00000051067	-1.74338017	1.74E-05
Nhsl1	ENSMUSG00000039835	-1.353955573	1.76E-05
Pcp4l1	ENSMUSG00000038370	-1.105278807	1.84E-05
Rnf149	ENSMUSG00000048234	1.448107457	1.89E-05
Rab15	ENSMUSG00000021062	2.345807011	1.90E-05
Gm13910	ENSMUSG00000063684	-0.855304946	1.95E-05
Epha4	ENSMUSG00000026235	-1.698949181	1.96E-05
Arhgef9	ENSMUSG00000025656	-1.143229357	1.99E-05
Snai3	ENSMUSG00000006587	-2.037015042	1.99E-05
Gm12167	ENSMUSG00000085853	-2.544107035	2.01E-05
Pdia4	ENSMUSG00000025823	0.966134463	2.01E-05
Ptgis	ENSMUSG00000017969	0.948962716	2.02E-05
Grb14	ENSMUSG00000026888	-1.093338547	2.06E-05
Zmym6	ENSMUSG00000042408	-1.58836033	2.07E-05
Il6st	ENSMUSG00000021756	-0.820999481	2.10E-05
S100a4	ENSMUSG00000001020	2.28746549	2.11E-05
Map3k6	ENSMUSG00000028862	1.086729604	2.13E-05
Dlg2	ENSMUSG00000052572	1.603224869	2.15E-05
Rab12	ENSMUSG00000023460	-0.725075259	2.18E-05
H2-Aa	ENSMUSG00000036594	1.045187439	2.18E-05
Camk2g	ENSMUSG00000021820	-0.995929728	2.20E-05
Ndufb4	ENSMUSG00000022820	-0.853806331	2.24E-05
Scx	ENSMUSG00000034161	1.215883451	2.25E-05
Crybb1	ENSMUSG00000029343	-2.27069208	2.29E-05
Actn1	ENSMUSG00000015143	1.071917258	2.29E-05
Nol12	ENSMUSG00000033099	0.960808572	2.30E-05
Dmpk	ENSMUSG00000030409	-0.863149993	2.31E-05
Ccr7	ENSMUSG00000037944	3.730833295	2.33E-05
Dio3	ENSMUSG00000075707	5.757495183	2.33E-05
Fsd2	ENSMUSG00000038663	-0.903666039	2.38E-05
Kif22	ENSMUSG00000030677	2.271624287	2.38E-05
Cks2	ENSMUSG00000062248	2.192445687	2.39E-05
Snx7	ENSMUSG00000028007	0.92097076	2.42E-05

Pgm1	ENSMUSG00000029171	1.160920678	2.42E-05
Esm1	ENSMUSG00000042379	3.597235783	2.42E-05
Ogdh	ENSMUSG00000020456	-0.910128034	2.45E-05
Hs3st5	ENSMUSG00000044499	-2.32700773	2.46E-05
Tmem100	ENSMUSG00000069763	1.114131856	2.48E-05
Rmnd1	ENSMUSG00000019763	-1.394623419	2.49E-05
1500009L16Rik	ENSMUSG00000087651	1.745244137	2.50E-05
Irx4	ENSMUSG00000021604	-1.13760894	2.54E-05
Atp6v0a4	ENSMUSG00000038600	2.707446108	2.55E-05
Usp39	ENSMUSG00000056305	0.92823482	2.56E-05
Bub1b	ENSMUSG00000040084	2.049703302	2.61E-05
Actn4	ENSMUSG00000054808	0.856694994	2.62E-05
Esr1	ENSMUSG00000019768	-7.269033491	2.73E-05
Paqr9	ENSMUSG00000064225	-1.000371855	2.75E-05
Sat1	ENSMUSG00000025283	0.941366578	2.78E-05
Cemip	ENSMUSG00000052353	6.866251091	2.81E-05
Ranbp1	ENSMUSG00000005732	1.016139942	2.83E-05
Tnni3k	ENSMUSG00000040086	-0.958877111	2.85E-05
Fastk	ENSMUSG00000028959	-0.850210517	2.86E-05
Il6	ENSMUSG00000025746	4.531344553	2.95E-05
A530016L24Rik	ENSMUSG00000043122	-1.081498026	2.99E-05
mt-Nd2	ENSMUSG00000064345	-1.142796124	2.99E-05
Abat	ENSMUSG00000057880	-1.373875704	3.01E-05
Mme	ENSMUSG00000027820	-1.5561673	3.01E-05
Htra3	ENSMUSG00000029096	-0.746493364	3.03E-05
Arhgap20	ENSMUSG00000053199	-1.399667673	3.06E-05
Armc2	ENSMUSG00000071324	-1.455121631	3.23E-05
Creb3	ENSMUSG00000028466	0.834075539	3.38E-05
Ldhb	ENSMUSG00000030246	-0.847516515	3.38E-05
Ap3s1	ENSMUSG00000024480	0.97582356	3.39E-05
Tspo	ENSMUSG00000041736	1.169018098	3.44E-05
Rtn2	ENSMUSG00000030401	-1.088627295	3.47E-05
Tnfsf10	ENSMUSG00000039304	-1.253019177	3.50E-05
Itga5	ENSMUSG00000000555	0.943488062	3.50E-05
Asb2	ENSMUSG00000021200	-0.990198446	3.52E-05
Cpt2	ENSMUSG00000028607	-1.020655591	3.54E-05
Irx3	ENSMUSG00000031734	-0.98892592	3.55E-05
Cct6a	ENSMUSG00000029447	0.728027756	3.59E-05
Cxcr6	ENSMUSG00000048521	2.967830317	3.60E-05
Spag5	ENSMUSG00000002055	3.017422173	3.61E-05

Adra1b	ENSMUSG00000050541	-1.302048765	3.61E-05
Ctf1	ENSMUSG00000042340	-1.7960565	3.63E-05
Mtch1	ENSMUSG00000024012	0.835350849	3.67E-05
Ufm1	ENSMUSG00000027746	0.85306856	3.70E-05
Lig1	ENSMUSG00000056394	1.207799015	3.71E-05
Adam11	ENSMUSG00000020926	-1.721931217	3.71E-05
Dhrs11	ENSMUSG00000034449	-0.819654272	3.75E-05
Set	ENSMUSG00000054766	0.8417092	3.81E-05
Slc20a2	ENSMUSG00000037656	-0.93306099	3.81E-05
E2f1	ENSMUSG00000027490	1.599715519	3.85E-05
Smarcd1	ENSMUSG00000023018	-1.057888152	3.87E-05
Rpl12	ENSMUSG00000038900	0.868899895	3.92E-05
Actr3b	ENSMUSG00000056367	-1.145843887	4.09E-05
Nox4	ENSMUSG00000030562	1.866410387	4.09E-05
Sema3f	ENSMUSG00000034684	0.991296099	4.09E-05
Chic2	ENSMUSG00000029229	0.919562298	4.09E-05
Igfbp3	ENSMUSG00000020427	-1.139152327	4.13E-05
Rbm20	ENSMUSG00000043639	-1.093998606	4.15E-05
Gem	ENSMUSG00000028214	1.480143375	4.15E-05
Eef1e1	ENSMUSG00000001707	0.869597969	4.16E-05
Fech	ENSMUSG00000024588	-0.733470059	4.18E-05
Yae1d1	ENSMUSG00000075054	-0.926999656	4.23E-05
Gpihbp1	ENSMUSG00000022579	0.979806221	4.26E-05
Pank1	ENSMUSG00000033610	-1.112156216	4.29E-05
Csrnp1	ENSMUSG00000032515	1.143864141	4.30E-05
Matn2	ENSMUSG00000022324	0.93757951	4.30E-05
Dap	ENSMUSG00000039168	1.060761224	4.45E-05
Lrp8	ENSMUSG00000028613	3.076927513	4.48E-05
Mtpn	ENSMUSG00000029840	0.7409373	4.50E-05
Cct5	ENSMUSG00000022234	0.789970885	4.54E-05
Pdia3	ENSMUSG00000027248	0.814069588	4.55E-05
Myo5b	ENSMUSG00000025885	-1.07591838	4.57E-05
Cmpk1	ENSMUSG00000028719	0.729331642	4.57E-05
Il34	ENSMUSG00000031750	1.736196194	4.60E-05
Cyb5r3	ENSMUSG00000018042	0.908778077	4.61E-05
Ky	ENSMUSG00000035606	-2.113931589	4.61E-05
Srpx2	ENSMUSG00000031253	1.217986039	4.62E-05
Tgfbr3	ENSMUSG00000029287	-0.872295846	4.71E-05
Gm13339	ENSMUSG00000082884	-2.612873165	4.74E-05
Nuf2	ENSMUSG00000026683	2.544415342	4.91E-05

Cnn2	ENSMUSG00000004665	0.774194332	4.92E-05
Gnb1	ENSMUSG00000029064	0.743748783	4.92E-05
Lyve1	ENSMUSG00000030787	0.923114282	4.94E-05
Fcrls	ENSMUSG00000015852	1.042470331	5.00E-05
Dpysl3	ENSMUSG00000024501	1.202522954	5.06E-05
Abca12	ENSMUSG00000050296	-2.468894466	5.11E-05
Rgma	ENSMUSG00000070509	-1.062393229	5.16E-05
Iqsec1	ENSMUSG00000034312	-1.010783788	5.16E-05
Lmnb1	ENSMUSG00000024590	1.459715437	5.18E-05
Ppp1r3d	ENSMUSG00000049999	-1.261594712	5.23E-05
Fbln5	ENSMUSG00000021186	1.028560124	5.23E-05
Ubr2	ENSMUSG00000023977	-0.891046088	5.27E-05
Eif3d	ENSMUSG00000016554	0.794490993	5.32E-05
Hax1	ENSMUSG00000027944	0.833813715	5.33E-05
Slmap	ENSMUSG00000021870	0.772173167	5.35E-05
Dcun1d2	ENSMUSG00000038506	-0.966239249	5.41E-05
Rpl10a	ENSMUSG00000037805	0.857995927	5.43E-05
Hbb-bt	ENSMUSG00000073940	1.197178887	5.44E-05
Anxa13	ENSMUSG00000055114	-2.786714981	5.45E-05
Palld	ENSMUSG00000058056	-0.799675206	5.46E-05
Tkt	ENSMUSG00000021957	1.004203743	5.46E-05
Has1	ENSMUSG00000003665	3.837181116	5.46E-05
Map1b	ENSMUSG00000052727	1.411116985	5.52E-05
Mpped2	ENSMUSG00000016386	-0.98067986	5.54E-05
Bax	ENSMUSG00000003873	1.089503608	5.59E-05
Pcdh19	ENSMUSG00000051323	1.210946192	5.62E-05
AA414768	ENSMUSG00000083307	1.654516139	5.62E-05
Txndc5	ENSMUSG00000038991	0.881548729	5.69E-05
Mmp15	ENSMUSG00000031790	-1.118266179	5.77E-05
Sdha	ENSMUSG00000021577	-0.746425645	5.80E-05
Adamts1	ENSMUSG00000022893	0.984573992	5.81E-05
Dio2	ENSMUSG00000007682	3.635128179	5.81E-05
Basp1	ENSMUSG00000045763	1.264249279	5.81E-05
Gm40604	ENSMUSG00000112800	-2.594640939	5.87E-05
Rpl6	ENSMUSG00000029614	0.731350539	5.98E-05
Cxcl16	ENSMUSG00000018920	1.128028466	6.00E-05
Rps16-ps2	ENSMUSG00000060419	0.879807144	6.04E-05
Pcolce	ENSMUSG00000029718	0.846899143	6.05E-05
Actr2	ENSMUSG00000020152	0.782201953	6.09E-05
Lsm14b	ENSMUSG00000039108	-0.855055459	6.11E-05

Nans	ENSMUSG00000028334	0.886230727	6.14E-05
Cdc42	ENSMUSG00000006699	0.658514014	6.16E-05
Rrp12	ENSMUSG00000035049	1.206615847	6.16E-05
Fbxo31	ENSMUSG00000052934	-0.894989711	6.19E-05
Tes	ENSMUSG00000029552	1.221804024	6.21E-05
Casp3	ENSMUSG00000031628	1.214705603	6.26E-05
Anp32b	ENSMUSG00000028333	0.753289197	6.26E-05
Mfn2	ENSMUSG00000029020	-0.888859823	6.29E-05
Rps3	ENSMUSG00000030744	0.744403445	6.33E-05
Lix1	ENSMUSG00000047786	-1.817311568	6.35E-05
Ubxn1	ENSMUSG00000071655	0.709328967	6.35E-05
Marcksl1	ENSMUSG00000047945	1.203000975	6.35E-05
Sh3rf2	ENSMUSG00000057719	-1.371037349	6.35E-05
Ssr1	ENSMUSG00000021427	0.784347935	6.48E-05
Sec61b	ENSMUSG00000053317	1.136801215	6.49E-05
1700123M08Rik	ENSMUSG00000085614	-1.5601323	6.53E-05
Dele1	ENSMUSG00000024442	-0.861700341	6.56E-05
Vegfa	ENSMUSG00000023951	0.775302173	6.58E-05
Clec4n	ENSMUSG00000023349	1.229335879	6.58E-05
Auh	ENSMUSG00000021460	-0.79811363	6.59E-05
Arrdc3	ENSMUSG00000074794	0.840312534	6.60E-05
Gm6472	ENSMUSG00000095597	0.826009687	6.69E-05
Sdc4	ENSMUSG00000017009	0.895973419	6.75E-05
mt-Co3	ENSMUSG00000064358	-2.192615357	6.79E-05
AI662270	ENSMUSG00000087107	1.694658366	6.86E-05
Foxo4	ENSMUSG00000042903	-0.830457396	7.06E-05
Nek2	ENSMUSG00000026622	2.787118381	7.08E-05
Mir22hg	ENSMUSG00000085148	1.28101779	7.10E-05
Dnal4	ENSMUSG00000022420	-0.923171237	7.11E-05
Crlf2	ENSMUSG00000033467	0.992785293	7.16E-05
Nostrin	ENSMUSG00000034738	1.339164421	7.16E-05
Tnfaip8l1	ENSMUSG00000044469	1.66480778	7.16E-05
Acox1	ENSMUSG00000020777	-0.766342238	7.18E-05
Pygm	ENSMUSG00000032648	-0.986421496	7.20E-05
Esrrg	ENSMUSG00000026610	-1.24361538	7.23E-05
Ak4	ENSMUSG00000028527	0.794324011	7.28E-05
Psmb10	ENSMUSG00000031897	0.866422125	7.30E-05
Slc36a2	ENSMUSG00000020264	-1.680849306	7.30E-05
Sdc1	ENSMUSG00000020592	1.145699644	7.48E-05
Atf5	ENSMUSG00000038539	0.780420286	7.50E-05

Slc3a2	ENSMUSG00000010095	0.824211271	7.51E-05
Bcas1	ENSMUSG00000013523	3.491613105	7.60E-05
Asb18	ENSMUSG00000067081	-1.289875759	7.63E-05
Leng8	ENSMUSG00000035545	-1.243145311	7.69E-05
Trim55	ENSMUSG00000060913	-0.764555649	7.74E-05
Fam131a	ENSMUSG00000050821	-1.138318769	7.74E-05
Frem1	ENSMUSG00000059049	2.502355246	7.83E-05
Kif4	ENSMUSG00000034311	2.342330785	8.04E-05
Slc27a1	ENSMUSG00000031808	-0.865631252	8.04E-05
Ppib	ENSMUSG00000032383	0.883266026	8.04E-05
AI506816	ENSMUSG00000105987	1.12017342	8.09E-05
Mrps34	ENSMUSG00000038880	0.723727808	8.10E-05
mt-Tm	ENSMUSG00000064344	-2.363755057	8.26E-05
H2-T22	ENSMUSG00000056116	0.814404995	8.29E-05
Hibadh	ENSMUSG00000029776	-0.802364755	8.51E-05
Npm1	ENSMUSG00000057113	0.765391834	8.52E-05
Edem1	ENSMUSG00000030104	0.898575858	8.54E-05
L2hgdh	ENSMUSG00000020988	-0.990232504	8.59E-05
Nubp1	ENSMUSG00000022503	1.06770218	8.67E-05
Fitm2	ENSMUSG00000048486	-0.918844762	8.67E-05
mt-Nd5	ENSMUSG00000064367	-1.145512901	8.69E-05
Col6a3	ENSMUSG00000048126	0.954494112	8.69E-05
Asb10	ENSMUSG00000038204	-0.949914111	8.87E-05
Adam15	ENSMUSG00000028041	0.826110171	8.94E-05
Glipr1	ENSMUSG00000056888	1.791520777	9.01E-05
Cxcr4	ENSMUSG00000045382	1.685998187	9.04E-05
Ftl1	ENSMUSG00000050708	0.778677718	9.11E-05
Cks1b	ENSMUSG00000028044	1.311450392	9.13E-05
Ninj1	ENSMUSG00000037966	0.699219979	9.24E-05
Ctsk	ENSMUSG00000028111	1.117524439	9.40E-05
Zfp30	ENSMUSG00000047473	-1.25754888	9.43E-05
Tent5b	ENSMUSG00000046694	2.22113418	9.56E-05
Ell2	ENSMUSG00000001542	0.994864886	9.60E-05
Slc22a3	ENSMUSG00000023828	-1.836470718	9.64E-05
Hsd17b7	ENSMUSG00000026675	-1.442163041	9.74E-05
Kcnv2	ENSMUSG00000047298	-2.312093364	9.74E-05
Aox3	ENSMUSG00000064294	-2.594162935	9.74E-05
Lrrc8a	ENSMUSG00000007476	0.953560263	9.86E-05
Ddah2	ENSMUSG00000007039	0.822942817	9.91E-05
Map7	ENSMUSG00000019996	-1.076724288	9.96E-05

Gm826	ENSMUSG00000074623	-1.854150746	9.99E-05
Ssu2	ENSMUSG00000034387	1.744520428	0.000100979
Pamr1	ENSMUSG00000027188	1.442207684	0.000101479
Nudt6	ENSMUSG00000050174	-1.305114648	0.000102303
Smim8	ENSMUSG00000028295	-0.77796519	0.0001024
Galm	ENSMUSG00000035473	-1.052715333	0.0001024
Dnajb4	ENSMUSG00000028035	0.799668691	0.000102672
Acad11	ENSMUSG00000090150	-1.084732814	0.000102971
AC105304.1	ENSMUSG00000117110	6.174955464	0.000103248
Dera	ENSMUSG00000030225	1.141257288	0.000105357
Cdv3	ENSMUSG00000032803	0.73861362	0.000106376
Cnst	ENSMUSG00000038949	-1.091648775	0.000108248
Tcf19	ENSMUSG00000050410	1.454493961	0.000108248
Cst6	ENSMUSG00000024846	1.537354611	0.000109393
Chd6	ENSMUSG00000057133	-1.129539479	0.000110687
Bcat2	ENSMUSG00000030826	-0.854118049	0.000111356
Per3	ENSMUSG00000028957	-0.997192104	0.000111797
Aldh1b1	ENSMUSG00000035561	-1.166657249	0.000112397
Nt5c3	ENSMUSG00000029780	-0.744316174	0.000112615
Sdcbp	ENSMUSG00000028249	0.68030449	0.000112781
Synpo2	ENSMUSG00000050315	-0.872030496	0.000112781
Upp1	ENSMUSG00000020407	1.348758602	0.000112869
Klhl21	ENSMUSG00000073700	-0.939146967	0.000113385
Lum	ENSMUSG00000036446	0.762133301	0.00011352
Gm14005	ENSMUSG00000074813	1.743756839	0.00011352
Gm48882	ENSMUSG00000112593	-1.544659664	0.000113753
Mapre1	ENSMUSG00000027479	0.727685037	0.000114064
Plekhh3	ENSMUSG00000035172	-0.988397449	0.000114064
Ywhaz	ENSMUSG00000022285	0.699675293	0.000114403
Slc10a6	ENSMUSG00000029321	1.367200475	0.000114763
Crispld1	ENSMUSG00000025776	2.078403359	0.000115859
Dclk1	ENSMUSG00000027797	1.27304377	0.000115859
1810014B01Rik	ENSMUSG00000097412	-1.331894755	0.000116234
Cd14	ENSMUSG00000051439	0.95973642	0.00011691
Fbxo21	ENSMUSG00000032898	-0.947688909	0.00011691
Ankrd23	ENSMUSG00000067653	1.187870943	0.000117977
AI464131	ENSMUSG00000046312	-1.477775898	0.000117977
Uxs1	ENSMUSG00000057363	1.004221926	0.000119455
Wnk4	ENSMUSG00000035112	-1.463317934	0.000119809
Nrg2	ENSMUSG00000060275	-2.630951901	0.000119844

Fam78a	ENSMUSG00000050592	-1.124255008	0.000120153
Tmem263	ENSMUSG00000060935	0.96480215	0.000120584
Slc25a38	ENSMUSG00000032519	-0.869125597	0.000122088
Ndc80	ENSMUSG00000024056	2.423154708	0.000122273
Sergef	ENSMUSG00000030839	1.245257306	0.000122273
Gm15972	ENSMUSG00000086287	-2.073687742	0.000122508
Rpl7	ENSMUSG00000043716	0.732676341	0.000123232
Hist2h2be	ENSMUSG00000068854	-1.354971961	0.000124167
Numa1	ENSMUSG00000066306	-0.797316904	0.000124746
Ccne2	ENSMUSG00000028212	1.429812172	0.00012482
R3hdm2	ENSMUSG00000025404	-0.840435036	0.00012482
Trim59	ENSMUSG00000034317	1.342907783	0.000126783
Tagln2	ENSMUSG00000026547	0.954159223	0.000126924
Ism1	ENSMUSG00000074766	1.672658909	0.000128065
F2r	ENSMUSG00000048376	0.915997412	0.000128451
Pcmtd2	ENSMUSG00000027589	-0.849265634	0.000128451
Itgav	ENSMUSG00000027087	1.030798434	0.000128451
Mybl1	ENSMUSG00000025912	2.380384328	0.000128692
Gng11	ENSMUSG00000032766	0.922982781	0.000128692
Slc25a13	ENSMUSG00000015112	-0.916356827	0.000129146
Edn1	ENSMUSG00000021367	1.497376085	0.00013157
Cebpg	ENSMUSG00000056216	0.91684615	0.000132187
Isg15	ENSMUSG00000035692	1.159210154	0.000133968
Fcgr3	ENSMUSG00000059498	1.041077436	0.000134132
Axin2	ENSMUSG00000000142	-2.07183498	0.000134132
Tcp11l2	ENSMUSG00000020034	-0.981214793	0.000134187
Pam	ENSMUSG00000026335	0.764379723	0.000135689
Yipf7	ENSMUSG00000029158	-0.997783466	0.000138131
Chaer1	ENSMUSG00000106783	-4.791012668	0.000139023
Grhl2	ENSMUSG00000022286	-3.948260681	0.000139023
Slc39a6	ENSMUSG00000024270	1.382031966	0.000140086
Fmnl3	ENSMUSG00000023008	0.899832997	0.000140086
Cul9	ENSMUSG00000040327	-0.97988309	0.00014023
Adamts7	ENSMUSG00000032363	-1.606884184	0.000140521
Nsa2	ENSMUSG00000060739	0.754882961	0.000140561
B4galnt1	ENSMUSG00000006731	2.236516517	0.000141619
Fgd4	ENSMUSG00000022788	-1.106356871	0.000142268
Wdr1	ENSMUSG00000005103	0.692222334	0.000143472
Rnf207	ENSMUSG00000058498	-1.151991286	0.000143472
Nip7	ENSMUSG00000031917	0.868193244	0.000145477

Ecscr	ENSMUSG00000073599	0.916766728	0.000146147
Slc25a16	ENSMUSG00000071253	-0.833230489	0.000148845
Ccl17	ENSMUSG00000031780	3.710638489	0.000149288
F830016B08Rik	ENSMUSG00000090942	-2.209342938	0.000150275
Bean1	ENSMUSG00000031872	2.018438196	0.000150275
Rps5	ENSMUSG00000012848	0.748254965	0.000151536
Gm10156	ENSMUSG00000066245	1.733016287	0.000151536
Strip2	ENSMUSG00000039629	-0.886836007	0.000151845
Gm6377	ENSMUSG00000048621	2.49656776	0.000152214
Rrm1	ENSMUSG00000030978	0.998165288	0.000153266
Mylk4	ENSMUSG00000044951	-1.252804454	0.000153322
Aldoa	ENSMUSG00000030695	0.79919769	0.000154398
Lama5	ENSMUSG00000015647	-1.094295776	0.000154488
Tmed3	ENSMUSG00000032353	0.827032169	0.000154702
Gm9780	ENSMUSG00000094800	1.700214932	0.000154702
Ifi30	ENSMUSG00000031838	1.114396586	0.000154702
Drg1	ENSMUSG00000020457	0.683105647	0.000154702
Gpr176	ENSMUSG00000040133	3.403658604	0.000154702
Mgat2	ENSMUSG00000043998	0.754830272	0.000157822
Exoc5	ENSMUSG00000061244	0.843070037	0.000159033
Ift122	ENSMUSG00000030323	1.122071062	0.000159532
Kif13a	ENSMUSG00000021375	-0.851981253	0.000159717
Sh2b1	ENSMUSG00000030733	-0.826373575	0.000160527
Nt5c	ENSMUSG00000020736	1.049244777	0.000161262
P4hb	ENSMUSG00000025130	0.87033207	0.000161262
Nfs1	ENSMUSG00000027618	-0.741319823	0.000161262
Oas1a	ENSMUSG00000052776	1.063757395	0.000162011
Arhgdia	ENSMUSG00000025132	0.726678269	0.000162242
Lmtk2	ENSMUSG00000038970	-1.21176516	0.000162882
Rpl24	ENSMUSG00000098274	1.153350314	0.000163063
Rnh1	ENSMUSG00000038650	0.742654696	0.000163258
Echs1	ENSMUSG00000025465	-0.771113442	0.000163779
Myl9	ENSMUSG00000067818	0.975365785	0.000164303
Wwtr1	ENSMUSG00000027803	0.725845511	0.000164779
H2afx	ENSMUSG00000049932	1.021756577	0.000166453
Gm47985	ENSMUSG00000114212	2.546883876	0.000166614
Gm19277	ENSMUSG00000115924	-1.974154043	0.000167209
Cntn5	ENSMUSG00000039488	-3.374525907	0.000169941
Lgr6	ENSMUSG00000042793	-1.310044826	0.00017165
Gzmm	ENSMUSG00000054206	-1.298100391	0.000173287

Gm27252	ENSMUSG00000098708	-2.101748683	0.000173287
Camta2	ENSMUSG00000040712	-0.797479434	0.000174688
2010111I01Rik	ENSMUSG00000021458	0.829764294	0.000175195
Esco2	ENSMUSG00000022034	2.862116432	0.000175644
Rras	ENSMUSG00000038387	0.836933427	0.000176266
Rpl17-ps3	ENSMUSG00000113948	0.711833545	0.000176266
Epb41l3	ENSMUSG00000024044	-1.546288962	0.000176878
Gm35177	ENSMUSG00000110922	-6.429040425	0.000177386
9-Sep	ENSMUSG00000059248	0.755976197	0.000177386
Pts	ENSMUSG00000032067	-0.834189968	0.000177386
Lmo4	ENSMUSG00000028266	0.734589636	0.000177386
Msn	ENSMUSG00000031207	0.799553541	0.000177386
Dtl	ENSMUSG00000037474	2.706604493	0.000180376
Shisa4	ENSMUSG00000041889	1.17966289	0.000180376
Neat1	ENSMUSG00000092274	-1.151454303	0.000181984
Aldh1a1	ENSMUSG00000053279	0.964640861	0.000185192
Atp6ap2	ENSMUSG00000031007	0.842076768	0.000186203
Gpr155	ENSMUSG00000041762	-1.32876739	0.000189622
Rcc2	ENSMUSG00000040945	0.876741319	0.000189798
Rras2	ENSMUSG00000055723	0.76401495	0.000189934
Ddc	ENSMUSG00000020182	-1.38250219	0.000191508
Mir99ahg	ENSMUSG00000090386	1.1102693	0.000192667
Zfp46	ENSMUSG00000051351	-1.027056956	0.000193753
Pccb	ENSMUSG00000032527	-0.698652862	0.000195815
H2-DMa	ENSMUSG00000037649	1.100925746	0.000196625
Rrm2b	ENSMUSG00000022292	-0.817929696	0.000196805
Cc2d2a	ENSMUSG00000039765	-1.122445661	0.000196984
Gm15543	ENSMUSG00000086863	-1.228508435	0.00019774
Phyh	ENSMUSG00000026664	-0.836723264	0.00019792
Oxsm	ENSMUSG00000021786	-0.924668801	0.000199967
Gm13680	ENSMUSG00000081400	1.044522345	0.000203116
Arpc2	ENSMUSG00000006304	0.672567384	0.000203481
Mphosph10	ENSMUSG00000030521	0.892693029	0.00020419
Pum3	ENSMUSG00000041360	0.773830198	0.000204605
Apbb1	ENSMUSG00000037032	-0.905188291	0.000204899
Cluh	ENSMUSG00000020741	-0.804196519	0.000204902
Prkar1a	ENSMUSG00000020612	0.621586336	0.000206102
Hmgn3	ENSMUSG00000066456	1.245901226	0.000206102
Fam117b	ENSMUSG00000041040	0.876595231	0.000206585
Gid4	ENSMUSG00000018415	-0.726585791	0.000209714

Aplp2	ENSMUSG00000031996	-0.726345216	0.000210175
Gm8430	ENSMUSG00000055093	1.074004178	0.000210175
Emilin1	ENSMUSG00000029163	0.799660093	0.000211184
3425401B19Rik	ENSMUSG00000071540	-1.148710484	0.000215128
Cd72	ENSMUSG00000028459	1.777472908	0.000215314
Rpl19	ENSMUSG00000017404	0.715816201	0.000215499
Fn3k	ENSMUSG00000025175	-0.977813005	0.000215516
Dpp4	ENSMUSG00000035000	-0.975539117	0.000217099
1700025G04Rik	ENSMUSG00000032666	0.788062319	0.000218142
Fam114a1	ENSMUSG00000029185	0.893863756	0.00021981
Piezo2	ENSMUSG00000041482	2.34241978	0.000219982
Tbrg1	ENSMUSG00000011114	0.807751257	0.000220928
Pea15a	ENSMUSG00000013698	0.712824257	0.000221491
Nbl1	ENSMUSG00000041120	0.827594761	0.000223625
Hectd4	ENSMUSG00000042744	-1.323369635	0.000223707
Pde2a	ENSMUSG00000110195	-2.460329707	0.000223853
Nfkb2	ENSMUSG00000025225	0.875363676	0.000224136
Tcea1	ENSMUSG00000033813	0.814236001	0.000224136
Mvp	ENSMUSG00000030681	0.811059052	0.000229252
Bcl2a1d	ENSMUSG00000099974	2.47385179	0.00023614
Ier5	ENSMUSG00000056708	1.178234042	0.00023614
Cep55	ENSMUSG00000024989	2.106963168	0.000236381
Ska1	ENSMUSG00000036223	2.939008556	0.000236986
Osbpl1a	ENSMUSG00000044252	-0.786046678	0.000238155
Runx3	ENSMUSG00000070691	2.527166412	0.000238313
Gm36377	ENSMUSG00000113909	6.333989748	0.000238673
Ch25h	ENSMUSG00000050370	2.261355686	0.000239183
Crat	ENSMUSG00000026853	-0.876036392	0.00023971
Smim19	ENSMUSG00000031534	-0.790869136	0.00024107
Cope	ENSMUSG00000055681	0.673991768	0.000242024
Bgn	ENSMUSG00000031375	0.877221011	0.000242024
Prnp	ENSMUSG00000079037	0.685726585	0.000242398
Arfgap3	ENSMUSG00000054277	1.08586968	0.000243569
Fam162a	ENSMUSG00000003955	0.874928041	0.000244213
Fam129b	ENSMUSG00000026796	0.720263432	0.000244797
Oplah	ENSMUSG00000022562	-0.835767828	0.000244797
Map3k4	ENSMUSG00000014426	-0.881797639	0.000244797
Twf1	ENSMUSG00000022451	0.830535821	0.00024495
Phkg1	ENSMUSG00000025537	-1.594751257	0.00024495
Gpr137c	ENSMUSG00000049092	-1.766008063	0.00024495

Tlr4	ENSMUSG00000039005	0.954075891	0.00024495
Fam107a	ENSMUSG00000021750	1.184025232	0.000247396
Cmss1	ENSMUSG00000022748	-1.115170298	0.000247796
Garem2	ENSMUSG00000044576	5.972407883	0.000249558
Setd1b	ENSMUSG00000038384	-1.296920375	0.000249558
Gm45670	ENSMUSG00000109635	-1.041440929	0.000258956
Atxn10	ENSMUSG00000016541	0.93295456	0.000258956
Mcee	ENSMUSG00000033429	-0.787805686	0.000258956
Hcn4	ENSMUSG00000032338	-2.108816201	0.000259326
Tnmd	ENSMUSG00000031250	5.92033147	0.000262712
Rit1	ENSMUSG00000028057	-0.749251877	0.000264111
Klrb1a	ENSMUSG00000030361	6.386504613	0.000264722
Zfp445	ENSMUSG00000047036	-0.832882631	0.000265248
E030044B06Rik	ENSMUSG00000097740	-6.358287039	0.000267475
Pkm	ENSMUSG00000032294	0.748617133	0.000267575
Gm14097	ENSMUSG00000084839	-2.534896431	0.000267575
Bola3	ENSMUSG00000045160	-0.958485386	0.000267575
Tbc1d16	ENSMUSG00000039976	-1.031882917	0.00026813
Myrf	ENSMUSG00000036098	-1.394853591	0.000269673
Nufip1	ENSMUSG00000022009	1.077829776	0.000270091
Nsun2	ENSMUSG00000021595	0.800139024	0.000271507
Lockd	ENSMUSG00000098318	3.106664873	0.000271963
Gm9892	ENSMUSG00000052825	0.939809863	0.000271963
Ice1	ENSMUSG00000034525	-0.925559404	0.000271963
Coq2	ENSMUSG00000029319	-0.755241183	0.000274267
Tmem150c	ENSMUSG00000050640	-1.782753709	0.000275712
Wdr75	ENSMUSG00000025995	0.845581168	0.000275712
Pcna	ENSMUSG00000027342	0.797275072	0.000278766
Efemp1	ENSMUSG00000020467	-1.130966568	0.000278766
Thsd4	ENSMUSG00000032289	-1.530076277	0.000282864
Mcm3	ENSMUSG00000041859	1.095857146	0.000286209
Dixdc1	ENSMUSG00000032064	-1.756193466	0.000286727
Hbb-bs	ENSMUSG00000052305	1.083686857	0.000288823
Gramd4	ENSMUSG00000035900	-0.89073047	0.000289878
Abhd17a	ENSMUSG00000003346	0.678777784	0.000290331
Castor1	ENSMUSG00000020424	1.109594661	0.000290331
4921504A21Rik	ENSMUSG00000097626	-2.010676943	0.000294264
Dus4l	ENSMUSG00000020648	-1.318177976	0.000294264
Dcaf4	ENSMUSG00000021222	-1.058004159	0.000296342
Ptges3	ENSMUSG00000071072	0.759355093	0.000296897

Ltbp1	ENSMUSG00000001870	-0.765683293	0.000297105
Dnajc2	ENSMUSG00000029014	0.741902366	0.000298202
Col15a1	ENSMUSG00000028339	0.816323656	0.000298804
Rps15a	ENSMUSG00000008683	0.684323226	0.000300563
Eef1b2	ENSMUSG00000025967	0.674231027	0.000301515
Gm11382	ENSMUSG00000074973	5.223035374	0.000304235
Ech1	ENSMUSG00000053898	-0.761446562	0.000305527
E330011O21Rik	ENSMUSG00000109841	-1.709267938	0.000305981
Lamb2	ENSMUSG00000052911	-0.736344169	0.000307686
Gm44386	ENSMUSG00000107689	-1.107953053	0.000308423
Pex11a	ENSMUSG00000030545	-0.956794126	0.000308683
Gca	ENSMUSG00000026893	-1.039771637	0.000311298
Blcap	ENSMUSG00000067787	-0.841421988	0.000311381
Spire1	ENSMUSG00000024533	1.243020878	0.000313017
Lynx1	ENSMUSG00000022594	-0.758795346	0.000313585
Adss	ENSMUSG00000015961	0.926058961	0.000313682
P2ry6	ENSMUSG00000048779	0.909072134	0.000314661
Agpat4	ENSMUSG00000023827	1.113208064	0.000315115
Tnni3	ENSMUSG00000035458	-0.748180094	0.000320933
Col14a1	ENSMUSG00000022371	1.039547653	0.000321484
Cd24a	ENSMUSG00000047139	1.337707972	0.000323449
Grk5	ENSMUSG00000003228	0.866141393	0.000323584
Brca1	ENSMUSG00000017146	2.716505542	0.000325365
Nadk2	ENSMUSG00000022253	-0.789781309	0.000326058
Dclre1b	ENSMUSG00000027845	1.252176753	0.000326058
Heyl	ENSMUSG00000032744	1.040418874	0.000326058
Tgfb2	ENSMUSG00000039239	1.161424085	0.000326064
Aida	ENSMUSG00000042901	0.764429269	0.000327698
Ccl6	ENSMUSG00000018927	0.976986274	0.000330605
Tacc2	ENSMUSG00000030852	-0.739548022	0.000331078
Ramp3	ENSMUSG00000041046	1.202683583	0.000333032
Ablim1	ENSMUSG00000025085	-0.733672669	0.000337157
Cdnf	ENSMUSG00000039496	-1.210373947	0.000337205
Arhgap11a	ENSMUSG00000041219	1.45909038	0.000337743
Rps26	ENSMUSG00000025362	0.81885312	0.000338098
Rdm1	ENSMUSG00000010362	-0.824530705	0.000344204
Scgb1c1	ENSMUSG00000038801	-1.258528038	0.000346587
Rab3a	ENSMUSG00000031840	-1.005731182	0.000348844
Rpl5	ENSMUSG00000058558	0.669531474	0.000354693
Mrgprh	ENSMUSG00000059408	-2.418006509	0.000357271

Ttn	ENSMUSG00000051747	-0.906550604	0.00036022
Tpd52	ENSMUSG00000027506	0.892520119	0.00036022
Mcrip1	ENSMUSG00000061111	0.7969228	0.00036167
Ass1	ENSMUSG00000076441	1.320969019	0.000361886
Spta1	ENSMUSG00000026532	-1.100969323	0.000362243
Trim47	ENSMUSG00000020773	0.830078266	0.000363609
Itih5	ENSMUSG00000025780	1.16991427	0.000365012
Ddah1	ENSMUSG00000028194	2.478770614	0.000367647
Xbp1	ENSMUSG00000020484	0.74727034	0.000367674
Adi1	ENSMUSG00000020629	-0.88030003	0.000370578
Dusp1	ENSMUSG00000024190	0.977680826	0.000370618
mt-Nd4l	ENSMUSG00000065947	-2.903296435	0.000371757
Lpcat2	ENSMUSG00000033192	1.57433145	0.000371757
D10Jhu81e	ENSMUSG00000053329	-0.881365161	0.000375308
Gnai3	ENSMUSG00000000001	0.703741621	0.000375308
Clu	ENSMUSG00000022037	-0.722626929	0.000383291
H2-DMb1	ENSMUSG00000079547	1.179390822	0.000387753
Melk	ENSMUSG00000035683	2.782945927	0.00038845
Vegfb	ENSMUSG00000024962	-0.598271996	0.000391488
Arel1	ENSMUSG00000042350	-1.111788349	0.000391488
Tef	ENSMUSG00000022389	-0.677214465	0.000391763
Suox	ENSMUSG00000049858	-1.104365874	0.000392681
Mpv17	ENSMUSG00000107283	-0.706085174	0.000397716
Stat5a	ENSMUSG00000004043	-0.961274781	0.00039827
Erp29	ENSMUSG00000029616	0.703495438	0.000403179
Ttk	ENSMUSG00000038379	4.03711512	0.000403931
Kif5b	ENSMUSG00000006740	0.630939969	0.000404279
Gm13456	ENSMUSG00000082536	1.120715491	0.000404279
Kctd11	ENSMUSG00000046731	1.150664126	0.000404279
Slc11a1	ENSMUSG00000026177	0.977812705	0.000410106
Nudt5	ENSMUSG00000025817	0.936796254	0.000410686
Frmd5	ENSMUSG00000027238	0.77851802	0.00041221
6430710C18Rik	ENSMUSG00000085427	-5.8571592	0.000415315
Acads	ENSMUSG00000029545	-0.773438081	0.000416672
Dusp3	ENSMUSG00000003518	0.755976017	0.000416962
Atp1a2	ENSMUSG00000007097	-1.104746788	0.000418605
Bak1	ENSMUSG00000057789	0.792979957	0.000418605
Ostn	ENSMUSG00000052276	6.188176022	0.000418748
Raf1	ENSMUSG00000000441	-0.899105756	0.000419355
Snta1	ENSMUSG00000027488	0.842397067	0.000422016

Hsd17b10	ENSMUSG00000025260	-0.636670174	0.000422016
Mipep	ENSMUSG00000021993	-0.67570873	0.000426332
Pcx	ENSMUSG00000024892	-0.869376811	0.000426332
Fitm1	ENSMUSG00000022215	-1.104305872	0.000426332
Gap43	ENSMUSG00000047261	2.319482887	0.000427504
Macf1	ENSMUSG00000028649	-0.725908603	0.000429035
Myzap	ENSMUSG00000041361	-0.622676484	0.000432837
Peg13	ENSMUSG00000106847	-0.859440608	0.000433531
Mdfic	ENSMUSG00000041390	0.824403004	0.000433531
Sort1	ENSMUSG00000068747	-1.079925075	0.000435347
Slc46a3	ENSMUSG00000029650	-1.072292167	0.00043557
Ckb	ENSMUSG00000001270	0.791920214	0.000440762
Acaa2	ENSMUSG00000036880	-0.824465558	0.000447429
Osgepl1	ENSMUSG00000026096	-0.896659618	0.000451259
Huwe1	ENSMUSG00000025261	-0.888761559	0.000452448
Gm34934	ENSMUSG00000115100	-1.202524097	0.000460464
Ccbe1	ENSMUSG00000046318	-1.123748305	0.000465985
Art3	ENSMUSG00000034842	-0.7872147	0.00046714
Ptrh1	ENSMUSG00000053746	1.406416489	0.000476592
Cmtm8	ENSMUSG00000041012	-1.100397376	0.000482335
Aes	ENSMUSG00000054452	-0.61463961	0.000482335
Etf1	ENSMUSG00000024360	0.667818358	0.000482595
Gabrr2	ENSMUSG00000023267	-2.951621502	0.000483208
Dgat2	ENSMUSG00000030747	-0.818834106	0.000484138
Nxn	ENSMUSG00000020844	0.858101503	0.000485147
Serbp1	ENSMUSG00000036371	0.654999561	0.00048576
Trabd2b	ENSMUSG00000070867	-0.880499513	0.000487583
Ddt	ENSMUSG00000001666	-1.008514023	0.000488372
Mydgf	ENSMUSG00000019579	0.780348467	0.000488466
Naa50	ENSMUSG00000022698	0.657240124	0.000491131
Btf3	ENSMUSG00000021660	0.614693979	0.000491234
Lifr	ENSMUSG00000054263	-0.741763581	0.000494147
Rars	ENSMUSG00000018848	0.798713787	0.000495539
Pfkm	ENSMUSG00000033065	-0.808105264	0.000499982
Mapk14	ENSMUSG00000053436	-0.741130409	0.000501136
Phc2	ENSMUSG00000028796	0.701376982	0.000501136
Mgarp	ENSMUSG00000037161	2.659923844	0.000502031
Gab1	ENSMUSG00000031714	-0.815944281	0.000509517
Dapk3	ENSMUSG00000034974	0.696495248	0.000509517
Cnmd	ENSMUSG00000022025	-1.834222436	0.000509517

Gm15500	ENSMUSG00000086583	0.774503557	0.000509743
Abhd17b	ENSMUSG00000047368	0.836506266	0.000517605
Gm12960	ENSMUSG00000100514	1.107472809	0.000517767
Nme4	ENSMUSG00000024177	-1.190744379	0.000518084
Hook1	ENSMUSG00000028572	-1.123102752	0.000518911
Nras	ENSMUSG00000027852	0.788997234	0.000519727
Adra1a	ENSMUSG00000045875	-1.590394032	0.000519727
Atp6v0d1	ENSMUSG00000013160	0.643207734	0.000519727
Cstb	ENSMUSG00000005054	0.956366427	0.000527598
Gm28437	ENSMUSG00000101111	-1.058482884	0.000531501
Hfe2	ENSMUSG00000038403	-0.848489327	0.000531909
Bche	ENSMUSG00000027792	-1.092460206	0.000533651
2210408F21Rik	ENSMUSG00000087380	-1.085923673	0.000533651
Saysd1	ENSMUSG00000045107	0.878283907	0.000535208
Cd74	ENSMUSG00000024610	0.758394223	0.000542435
Ptgr1	ENSMUSG00000028378	1.058955926	0.000545236
Igfbp4	ENSMUSG00000017493	0.665805477	0.000547057
Crlf1	ENSMUSG00000007888	4.287227391	0.000550081
Sec31b	ENSMUSG00000051984	-1.732824823	0.000551339
Galk1	ENSMUSG00000020766	0.797163637	0.000551995
St3gal5	ENSMUSG00000056091	-0.875718232	0.000551995
Tead2	ENSMUSG00000030796	0.986076371	0.000554189
Stra6	ENSMUSG00000032327	-2.365328192	0.000554189
Crebrf	ENSMUSG00000048249	-0.79360851	0.000554778
Ugdh	ENSMUSG00000029201	0.893733773	0.000567666
Tuba1c	ENSMUSG00000043091	0.808802919	0.000569446
Ubqln4	ENSMUSG00000008604	-0.684042087	0.000571979
Rab5c	ENSMUSG00000019173	0.656147352	0.000572297
Aurkb	ENSMUSG00000020897	2.442037608	0.000572603
Tnnt2	ENSMUSG00000026414	-0.554251711	0.000572603
Tgfbr1	ENSMUSG00000007613	0.9967037	0.000574634
Rps10-ps2	ENSMUSG00000099764	0.672899736	0.00057611
Gnb4	ENSMUSG00000027669	0.89187788	0.00057657
Plk4	ENSMUSG00000025758	1.295173932	0.000581556
Atp5g2	ENSMUSG00000062683	-0.663567022	0.00058293
Eml2	ENSMUSG00000040811	-0.862513644	0.000584664
Adam12	ENSMUSG00000054555	3.210275572	0.000592973
Cdca8	ENSMUSG00000028873	2.02943011	0.000594957
Slc20a1	ENSMUSG00000027397	1.047144995	0.000595324
Hnrnpa1	ENSMUSG00000046434	0.650957093	0.000596047

Amotl2	ENSMUSG00000032531	-0.76446308	0.000597296
Rasl10b	ENSMUSG00000020684	-0.85035819	0.000599551
Gm14327	ENSMUSG00000074521	-0.981971509	0.000599815
Osbpl8	ENSMUSG00000020189	-0.71054343	0.000602915
Thrb	ENSMUSG00000021779	-1.118562563	0.000603078
Mrto4	ENSMUSG00000028741	0.787800509	0.000604879
B230110C06Rik	ENSMUSG00000097547	-1.783285037	0.000605242
Gm5637	ENSMUSG00000046993	1.264639339	0.000605302
Wdtc1	ENSMUSG00000037622	-0.768496393	0.000606327
Dsp	ENSMUSG00000054889	-0.921668445	0.000608888
Gm9385	ENSMUSG00000080848	1.003945883	0.000611734
Adcy9	ENSMUSG00000005580	-1.823398199	0.000615003
Gm4739	ENSMUSG00000112808	2.0077201	0.000617053
Hhipl1	ENSMUSG00000021260	1.280281106	0.000617053
Ywhaq	ENSMUSG00000076432	0.720288413	0.000617053
Sspn	ENSMUSG00000030255	-0.717960814	0.000617053
Decr1	ENSMUSG00000028223	-0.655534191	0.000626734
Map3k5	ENSMUSG00000071369	-0.955279279	0.000626734
Ssr2	ENSMUSG00000041355	0.702823559	0.000633837
Rps8	ENSMUSG00000047675	0.681583328	0.000633837
Slc25a26	ENSMUSG00000045100	-1.053227268	0.000638076
Col6a1	ENSMUSG00000001119	0.684657499	0.000638076
Prmt1	ENSMUSG00000109324	0.628357106	0.000640448
Amigo2	ENSMUSG00000048218	-0.975352562	0.000642948
Nsun4	ENSMUSG00000028706	-0.789931314	0.000643747
Fads3	ENSMUSG00000024664	0.965751907	0.000644133
Ripor2	ENSMUSG00000036006	-1.116947217	0.000644916
Entpd4b	ENSMUSG00000022066	-4.518676003	0.000651654
Opa1	ENSMUSG00000038084	-0.702782249	0.000651654
Bcl2l11	ENSMUSG00000027381	-1.09786291	0.000652474
Gm3646	ENSMUSG00000091937	-1.276925653	0.000661146
Dip2b	ENSMUSG00000023026	-0.888907973	0.000661146
Kctd12	ENSMUSG00000098557	0.912545293	0.000662341
Tinagl1	ENSMUSG00000028776	0.842275014	0.000663836
Cited2	ENSMUSG00000039910	-0.7022771	0.000663871
Rcn1	ENSMUSG00000005973	0.69984554	0.000666352
Ccdc86	ENSMUSG00000024732	0.881598761	0.000666659
Slfn9	ENSMUSG00000069793	1.337780947	0.000667442
Hsd11b1	ENSMUSG00000016194	-1.077729786	0.000669001
Gm32255	ENSMUSG00000112120	6.24548188	0.000669128

Slc45a3	ENSMUSG00000026435	1.035992003	0.000678814
Srpk3	ENSMUSG00000002007	-0.837138437	0.000678814
Tmem151a	ENSMUSG00000061451	-1.324000751	0.000682244
Bzw1	ENSMUSG00000051223	0.631141501	0.000685674
Acer2	ENSMUSG00000038007	-0.760824926	0.000685681
Prodh	ENSMUSG00000003526	-0.935661706	0.000686768
Nop14	ENSMUSG00000036693	0.875273117	0.000689044
Ipo4	ENSMUSG00000002319	0.802337523	0.000689289
Ncl	ENSMUSG00000026234	0.689104513	0.000689289
Tmem37	ENSMUSG00000050777	0.951734898	0.000689289
Tmf1	ENSMUSG00000030059	0.856873578	0.000692952
Chpf	ENSMUSG00000032997	0.782531234	0.000699294
4933431K23Rik	ENSMUSG00000086451	-1.382440266	0.000700632
Akip1	ENSMUSG00000031023	0.86800718	0.000705593
Zfp36	ENSMUSG00000044786	0.923126588	0.000709504
Lrrc59	ENSMUSG00000020869	0.895454605	0.000709896
Pes1	ENSMUSG00000020430	0.71932661	0.000712484
Rbm7	ENSMUSG00000042396	0.691504614	0.000714693
Rpl4	ENSMUSG00000032399	0.691815175	0.000722951
Rad51ap1	ENSMUSG00000030346	1.806750333	0.000725171
Fbxl5	ENSMUSG00000039753	-0.64070929	0.00072573
Tspan4	ENSMUSG00000025511	0.656694903	0.000729642
Tpt1-ps3	ENSMUSG00000084319	0.645433901	0.000736126
Ptpn2	ENSMUSG00000024539	0.764000253	0.000737553
Mllt11	ENSMUSG00000053192	0.866314769	0.000737553
Wdr43	ENSMUSG00000041057	0.771960976	0.000739971
Pik3c2b	ENSMUSG00000026447	-0.906710768	0.000741655
Cd28	ENSMUSG00000026012	-2.047524239	0.000742016
Gzmk	ENSMUSG00000042385	6.197349732	0.000744554
Cdip1	ENSMUSG00000004071	-0.717953569	0.00074467
Mvb12a	ENSMUSG00000031813	0.714173574	0.000746169
Ddo	ENSMUSG00000063428	-0.824268739	0.000747054
Ywhah	ENSMUSG00000018965	0.653884827	0.000750028
Dcaf12l1	ENSMUSG00000045284	-1.535196883	0.000755307
CN725425	ENSMUSG00000078932	-3.314084355	0.000760701
Syne2	ENSMUSG00000063450	-0.913820486	0.000761118
2310061I04Rik	ENSMUSG00000050705	-0.833540195	0.000761629
Dennd4b	ENSMUSG00000042404	-0.889897397	0.000766287
Arhgef19	ENSMUSG00000028919	-0.817480742	0.000772443
Ccl11	ENSMUSG00000020676	-1.899440068	0.000777802

Rhob	ENSMUSG00000054364	0.646873373	0.000781387
Rpl17-ps10	ENSMUSG00000081895	0.787909324	0.000791775
Carns1	ENSMUSG00000075289	-1.146964882	0.000791775
Cebpb	ENSMUSG00000056501	0.962714726	0.000791775
Ackr2	ENSMUSG00000044534	1.310402143	0.000792677
Gm44220	ENSMUSG00000107655	-2.041903606	0.000792677
Igdcc4	ENSMUSG00000032816	-1.179853997	0.000792677
Dpyd	ENSMUSG00000033308	-1.133089618	0.000798035
Rpl26	ENSMUSG00000060938	0.678803248	0.000801061
Trp53bp1	ENSMUSG00000043909	-0.994669252	0.000814536
Cdc25c	ENSMUSG00000044201	4.274952589	0.000815251
Rbl2	ENSMUSG00000031666	-0.843519264	0.000817339
Epm2a	ENSMUSG00000055493	-0.795889566	0.000819271
Kntc1	ENSMUSG00000029414	3.210968984	0.000826757
Efnb3	ENSMUSG00000003934	-2.729343078	0.000836266
Gas2	ENSMUSG00000030498	1.135148087	0.000837839
Pdlim7	ENSMUSG00000021493	0.752715046	0.000838753
Cox6a2	ENSMUSG00000030785	-0.75369274	0.000839324
Gm9843	ENSMUSG00000050299	0.847307762	0.000846977
Serpini1	ENSMUSG00000027834	1.387789281	0.000846977
Serpina3i	ENSMUSG00000079014	2.658266307	0.000847157
Gm12751	ENSMUSG00000062554	0.867792739	0.000848317
Gstm6	ENSMUSG00000068762	-2.199371288	0.000849211
Omd	ENSMUSG00000048368	1.255417814	0.000850235
Calu	ENSMUSG00000029767	0.59837872	0.000850235
1700066B17Rik	ENSMUSG00000101634	-6.18576287	0.000853418
Gigyf1	ENSMUSG00000029714	-1.040557676	0.000856633
Il10rb	ENSMUSG00000022969	-0.682544978	0.000864283
Dpysl2	ENSMUSG00000022048	0.950085579	0.000865728
Tmem237	ENSMUSG00000038079	1.12886864	0.000867201
Adamts9	ENSMUSG00000030022	1.289158891	0.000870869
Cndp2	ENSMUSG00000024644	0.695174388	0.000890344
Bcor	ENSMUSG00000040363	-0.798193765	0.000893807
Cx3cl1	ENSMUSG00000031778	1.238693125	0.000895289
Cdt1	ENSMUSG00000006585	1.555965797	0.000904322
Slc26a6	ENSMUSG00000023259	-1.157151682	0.000907547
Ghr	ENSMUSG00000055737	-0.642134932	0.000907902
Irgm2	ENSMUSG00000069874	-0.919134265	0.000910118
Pym1	ENSMUSG00000064030	0.871385358	0.000914505
Abcc8	ENSMUSG00000040136	-0.831736586	0.000915672

Ctsa	ENSMUSG00000017760	0.719032954	0.000915672
Fkbp14	ENSMUSG00000038074	0.854974151	0.000915672
Scarf2	ENSMUSG00000012017	1.016674215	0.000915672
Ncapg	ENSMUSG00000015880	2.349484229	0.00091647
Gpm6a	ENSMUSG00000031517	-0.697805669	0.00091647
Fkbp4	ENSMUSG00000030357	-0.93571143	0.000917202
0610025J13Rik	ENSMUSG00000046683	-2.449838125	0.00091744
Uchl1	ENSMUSG00000029223	1.096434049	0.000917912
Scn4b	ENSMUSG00000046480	-2.846668085	0.000920106
Nudcd2	ENSMUSG00000020328	0.745934586	0.000920603
Mis18bp1	ENSMUSG00000047534	2.525461744	0.000925697
Bop1	ENSMUSG00000022557	0.723107927	0.000929255
Pxmp4	ENSMUSG00000000876	-0.812223208	0.000936181
Gm48483	ENSMUSG00000111318	-2.873006923	0.000940052
Rpl7a	ENSMUSG00000062647	0.699419828	0.000940396
Cd63-ps	ENSMUSG00000085939	0.949996381	0.00094379
Spdl1	ENSMUSG00000069910	2.966011046	0.00094379
Hba-a1	ENSMUSG00000069919	1.053543442	0.000944371
Gm28438	ENSMUSG00000101939	-3.496378458	0.000946628
F2rl1	ENSMUSG00000021678	2.11390853	0.000946628
Tmem63b	ENSMUSG00000036026	-0.903631976	0.000950841
Hdac9	ENSMUSG00000004698	-1.060397137	0.000952622
Tnip1	ENSMUSG00000020400	0.923222763	0.000959597
Thumpd3	ENSMUSG00000030264	0.764370644	0.000960547
Rab13	ENSMUSG00000027935	1.080009546	0.000974303
Arl6ip1	ENSMUSG00000030654	0.692582835	0.000977649
Ntan1	ENSMUSG00000022681	0.675644197	0.000977664
Tmem25	ENSMUSG00000002032	-1.160779661	0.000979384
Gm11899	ENSMUSG00000087399	-2.027075533	0.00098408
Oxld1	ENSMUSG00000039670	-0.815669839	0.000988572
Fbxw9	ENSMUSG00000008167	1.331607951	0.00099152
Smg6	ENSMUSG00000038290	-0.898398837	0.000991787
Ppp1r3a	ENSMUSG00000042717	-0.790368781	0.00100233
Nucb2	ENSMUSG00000030659	0.866442047	0.001016271
Comtd1	ENSMUSG00000021773	1.751589768	0.001017096
Npc2	ENSMUSG00000021242	0.768807087	0.001018708
BC023105	ENSMUSG00000063388	-2.133477658	0.001027514
Phlpp1	ENSMUSG00000044340	-0.980724431	0.00104124
Pde3a	ENSMUSG00000041741	-0.794291636	0.001041286
Pde4dip	ENSMUSG00000038170	-0.817716042	0.001043736

Thoc6	ENSMUSG00000041319	0.946241006	0.001048174
Gm9493	ENSMUSG00000044424	0.994306645	0.001056697
Oxr1	ENSMUSG00000022307	-0.637235895	0.001058663
Ppil1	ENSMUSG00000024007	-0.901676619	0.001069443
Neo1	ENSMUSG00000032340	-0.857796785	0.001073037
Slco5a1	ENSMUSG00000025938	-1.479539222	0.001075603
Mgl2	ENSMUSG00000040950	-1.203780273	0.00107625
Tlr7	ENSMUSG00000044583	1.029824526	0.001078569
Nudt12	ENSMUSG00000024228	-1.074539742	0.001081735
Pik3r1	ENSMUSG00000041417	-0.776336007	0.001084974
Clec18a	ENSMUSG00000033633	-3.781448994	0.001087689
Ppp2r3a	ENSMUSG00000043154	-0.785966955	0.001087689
Klhl23	ENSMUSG00000042155	-0.969431836	0.00109029
Gm17251	ENSMUSG00000090952	-1.887525106	0.001093923
2900076A07Rik	ENSMUSG00000097277	-1.221695828	0.001102112
Rpl8	ENSMUSG00000003970	0.567214572	0.001103792
Ikbip	ENSMUSG00000019975	0.743144072	0.001104728
Fam185a	ENSMUSG00000047221	-0.912697428	0.001105476
Trip13	ENSMUSG00000021569	1.949471405	0.001105631
Pptc7	ENSMUSG00000038582	-0.889156957	0.001105917
Sox12	ENSMUSG00000051817	-0.901826147	0.001107394
Zfyve26	ENSMUSG00000066440	-1.267781892	0.001107394
Gm13341	ENSMUSG00000083863	-1.368820284	0.001107394
Enc1	ENSMUSG00000041773	1.05531106	0.001108073
Cnot6	ENSMUSG00000020362	0.708771192	0.001110728
Col18a1	ENSMUSG00000001435	1.001179643	0.001111441
Cacnb3	ENSMUSG00000003352	1.323406446	0.001111441
Ripor1	ENSMUSG00000038604	-0.682606882	0.001111441
Ppp1r12b	ENSMUSG00000073557	-0.893323129	0.001120535
Firre	ENSMUSG00000085396	-1.247263647	0.001128658
Camk2b	ENSMUSG00000057897	-1.074842563	0.001138546
Gm5069	ENSMUSG00000055676	-2.019397523	0.0011483
Dvl3	ENSMUSG00000003233	-0.806182403	0.001158463
Sri	ENSMUSG00000003161	0.653209692	0.001159764
Atg101	ENSMUSG00000037204	0.871594836	0.001162084
Tpcn1	ENSMUSG00000032741	-0.697369218	0.001167394
Creg1	ENSMUSG00000040713	-0.629557251	0.001167394
Tmem201	ENSMUSG00000044700	-0.738451626	0.001168322
Kcnq1	ENSMUSG00000009545	-0.934815657	0.001180797
Oxct1	ENSMUSG00000022186	-0.761204381	0.001187423

Osbpl6	ENSMUSG00000042359	-1.190333221	0.001189264
4833412C05Rik	ENSMUSG00000097697	3.149782282	0.001200201
A4galt	ENSMUSG00000047878	0.870065617	0.001200201
Traip	ENSMUSG00000032586	2.069819255	0.001202952
Atp8a1	ENSMUSG00000037685	-0.85898948	0.001205405
Nkd2	ENSMUSG00000021567	1.581666942	0.001213253
Siah2	ENSMUSG00000036432	0.901389441	0.00121691
Psmd11	ENSMUSG00000017428	0.667212163	0.001221548
Vkorc1	ENSMUSG00000096145	1.400708593	0.001222673
Nek6	ENSMUSG00000026749	1.121597695	0.001223391
Jam2	ENSMUSG00000053062	-0.638110974	0.001223391
Ddx21	ENSMUSG00000020075	0.734724922	0.001224446
Smg5	ENSMUSG00000001415	-0.649999022	0.001227439
Vldlr	ENSMUSG00000024924	-0.870126327	0.001228658
Mapk1ip1	ENSMUSG00000041775	-0.947853745	0.001238286
Hecw2	ENSMUSG00000042807	1.073487906	0.001239528
Ucp2	ENSMUSG00000033685	0.637508237	0.001242462
Gm33016	ENSMUSG00000113278	-5.56993038	0.001242462
Mcf2l	ENSMUSG00000031442	0.910389852	0.00124709
Dapp1	ENSMUSG00000028159	-0.998877536	0.001254827
Eda2r	ENSMUSG00000034457	1.086493658	0.001257694
Klrb1b	ENSMUSG00000079298	3.848183139	0.001260408
Myh6	ENSMUSG00000040752	-0.991428815	0.001260408
Slamf7	ENSMUSG00000038179	2.061142916	0.00126259
Lpxn	ENSMUSG00000024696	1.29341031	0.00126785
Rab32	ENSMUSG00000019832	1.134954603	0.001267976
Gm9761	ENSMUSG00000034437	1.201179665	0.001268445
Nuak1	ENSMUSG00000020032	0.705586296	0.001273756
Dhrs4	ENSMUSG00000022210	-0.710035325	0.001282516
Rai2	ENSMUSG00000043518	-0.72169745	0.001284474
Fgf13	ENSMUSG00000031137	-0.872284874	0.001306632
Pdpn	ENSMUSG00000028583	0.8958609	0.001319459
Rtn4ip1	ENSMUSG00000019864	-0.932475252	0.001329999
Siae	ENSMUSG00000001942	-0.946185844	0.001333879
Nudt18	ENSMUSG00000045211	0.753825634	0.001334036
Ifi204	ENSMUSG00000073489	0.753299552	0.001336706
Pald1	ENSMUSG00000020092	0.788980758	0.001339844
Gm14681	ENSMUSG00000081603	1.125973591	0.001343563
Cd3g	ENSMUSG00000002033	2.430685011	0.001347423
Atp5s	ENSMUSG00000054894	-0.776781135	0.001348366

Foxo6	ENSMUSG00000052135	-1.718622905	0.001349709
Cenpe	ENSMUSG00000045328	2.278966186	0.001351414
Gm3379	ENSMUSG00000113625	1.677066167	0.001352159
Epha7	ENSMUSG00000028289	-1.220177843	0.001358274
Eid1	ENSMUSG00000091337	0.700225615	0.001359256
Cct8	ENSMUSG00000025613	0.589547156	0.001367727
Atp6v1a	ENSMUSG00000052459	0.640752663	0.001367727
Synpo	ENSMUSG00000043079	0.719446982	0.001378454
Gstt1	ENSMUSG00000001663	-0.977554379	0.001384638
Mdh2	ENSMUSG00000019179	-0.633776273	0.001384638
Znrd1as	ENSMUSG00000036214	-0.832644772	0.001387155
Utp3	ENSMUSG00000070697	0.651352862	0.001394224
Abce1	ENSMUSG00000058355	0.693966902	0.001396718
Wnk1	ENSMUSG00000045962	-0.755941286	0.001401193
Ctps	ENSMUSG00000028633	0.639340774	0.00141368
Tax1bp3	ENSMUSG00000040158	0.718751774	0.001430447
Ndufab1	ENSMUSG00000030869	-0.68681487	0.001430447
Cdkl5	ENSMUSG00000031292	-1.685740279	0.001430447
Nsmaf	ENSMUSG00000028245	-0.635825855	0.001437597
Hcls1	ENSMUSG00000022831	0.801395708	0.001437597
Akap7	ENSMUSG00000039166	-0.763734043	0.001444405
Cenpw	ENSMUSG00000075266	1.816404216	0.00144837
Gm17383	ENSMUSG00000058625	2.172061557	0.001449941
Tmem183a	ENSMUSG00000042305	0.69981926	0.00145113
Gm11847	ENSMUSG00000060989	1.125416906	0.001456687
Uap1l1	ENSMUSG00000026956	0.886868491	0.001460122
Ankrd2	ENSMUSG00000025172	2.734152511	0.00146108
3300005D01Rik	ENSMUSG00000096965	5.921812483	0.001465075
Nppa	ENSMUSG00000041616	2.685827571	0.001465763
Sh3bgrl3	ENSMUSG00000028843	0.874064595	0.001475111
Tfb2m	ENSMUSG00000026492	-0.652855557	0.001475751
Nudt11	ENSMUSG00000073295	-1.486163512	0.001478892
Ube2b	ENSMUSG00000020390	-0.663363267	0.001479157
AC165271.1	ENSMUSG00000116641	2.103961047	0.00148352
Havcr2	ENSMUSG00000020399	1.97438735	0.001491174
Igf2bp2	ENSMUSG00000033581	1.255926133	0.001492354
Myoz1	ENSMUSG00000068697	2.795929269	0.001492354
Tsta3	ENSMUSG00000022570	0.72026626	0.0014965
Ndufv3	ENSMUSG00000024038	-0.782511846	0.0014965
Klhl8	ENSMUSG00000029312	-0.954041413	0.001506329

Gba	ENSMUSG00000028048	0.794290443	0.001516283
Sun3	ENSMUSG00000040985	-2.864814886	0.001519311
Foxm1	ENSMUSG00000001517	2.150986709	0.001529235
Ifngr1	ENSMUSG00000020009	0.734488752	0.001530861
Tcta	ENSMUSG00000039461	-0.820671687	0.001535604
Atp6v0e2	ENSMUSG00000039347	-0.9733121	0.001539027
B4galt2	ENSMUSG00000028541	0.994917979	0.001539133
Adamts16	ENSMUSG00000049538	6.055875072	0.001539774
Slc35e4	ENSMUSG00000048807	0.901806985	0.001540315
Tex30	ENSMUSG00000026049	1.167808354	0.00154039
Acat1	ENSMUSG00000032047	-0.677326114	0.0015443
Zfp365	ENSMUSG00000037855	1.584955017	0.001551872
Trmt6	ENSMUSG00000037376	0.935336865	0.001552133
Gprasp1	ENSMUSG00000043384	-0.858623319	0.001554754
Pdpr	ENSMUSG00000033624	-1.274185936	0.001561897
Pdxp	ENSMUSG00000116165	1.849264484	0.001561897
Gm31520	ENSMUSG00000107653	-1.891341564	0.001561897
Sipa1l2	ENSMUSG00000001995	-0.948627657	0.001562991
Nusap1	ENSMUSG00000027306	1.614347779	0.001582334
Knl1	ENSMUSG00000027326	3.124722863	0.001585599
Pak1	ENSMUSG00000030774	1.204320313	0.001590206
Uba2	ENSMUSG00000052997	0.636796923	0.001590206
Ppm1l	ENSMUSG00000027784	-1.077649173	0.001599707
Gm11967	ENSMUSG00000084819	1.535157668	0.001618657
Tlcd1	ENSMUSG00000019437	-1.169641054	0.001619959
Arfgef1	ENSMUSG00000067851	-0.695291564	0.001625654
Mgp	ENSMUSG00000030218	1.034728632	0.001628324
Acp5	ENSMUSG00000001348	1.810121608	0.00162948
Plekhn1	ENSMUSG00000078485	-1.018945077	0.001642432
9330158H04Rik	ENSMUSG00000073154	-0.759436512	0.001656498
Mastl	ENSMUSG00000026779	2.032004525	0.001663524
Atp5j	ENSMUSG00000022890	-0.719287295	0.001666413
Fmr1	ENSMUSG00000000838	0.855439836	0.001667939
Mcm10	ENSMUSG00000026669	3.420356964	0.001671845
Parp1	ENSMUSG00000026496	-0.68004862	0.001671845
Scrn3	ENSMUSG00000008226	-0.722463338	0.00167482
Ubap2	ENSMUSG00000028433	-0.783677311	0.00167482
Prcp	ENSMUSG00000061119	0.74532441	0.001680598
Pafah1b2	ENSMUSG00000003131	0.604584402	0.001689551
Cacnb2	ENSMUSG00000057914	-0.83996649	0.001694212

Gaa	ENSMUSG00000025579	-0.6405244	0.001697347
Adssl1	ENSMUSG00000011148	0.573238703	0.001700592
Spata33	ENSMUSG00000048478	-0.989362907	0.001701607
Gpr171	ENSMUSG00000050075	3.307363818	0.001701607
Svip	ENSMUSG00000074093	-0.860635281	0.001701607
Abi3bp	ENSMUSG00000035258	0.965253188	0.001708349
Adamtsl4	ENSMUSG00000015850	-0.79839088	0.001726183
Tsc22d3	ENSMUSG00000031431	0.900012206	0.001726183
9030612E09Rik	ENSMUSG00000045008	-1.731017191	0.001732862
Sardh	ENSMUSG00000009614	0.989183207	0.001733325
Pafah1b3	ENSMUSG00000005447	1.279080237	0.0017348
Tmem50b	ENSMUSG00000022964	-0.659934191	0.001747797
Atp6v0e	ENSMUSG00000015575	0.636771906	0.001751759
Hrct1	ENSMUSG00000071001	1.027299365	0.001756657
Sapcd2	ENSMUSG00000026955	3.214331414	0.001760439
Polg	ENSMUSG00000039176	-0.757307612	0.001766254
Nod1	ENSMUSG00000038058	-0.823018246	0.001771919
Vars2	ENSMUSG00000038838	-0.923331262	0.001773842
Pdha1	ENSMUSG00000031299	-0.667204344	0.001776268
Sp3os	ENSMUSG00000063714	-1.104813807	0.00180353
Papss1	ENSMUSG00000028032	0.776782363	0.001804969
Gnpat	ENSMUSG00000031985	-0.691658954	0.001809063
Mettl11b	ENSMUSG00000040113	-2.886940064	0.00181091
Sybu	ENSMUSG00000022340	-1.600039084	0.00181091
Arhgef17	ENSMUSG00000032875	-0.851675489	0.001813841
Cntrl	ENSMUSG00000057110	-1.08146091	0.001815145
Olfml3	ENSMUSG00000027848	0.657459845	0.001815145
Tmem8b	ENSMUSG00000078716	-0.912493406	0.001818525
Tlr13	ENSMUSG00000033777	1.14773615	0.001827605
Irx5	ENSMUSG00000031737	-0.950672407	0.001842892
Gm8991	ENSMUSG00000059179	1.233374827	0.001847634
Kif2c	ENSMUSG00000028678	2.393451981	0.001852469
Gm14150	ENSMUSG00000082809	1.812394273	0.001852469
Chaf1b	ENSMUSG00000022945	1.771321327	0.001852472
Slc25a5	ENSMUSG00000016319	0.56497081	0.001853005
Zwilch	ENSMUSG00000032400	2.065849002	0.001856036
Cyp2s1	ENSMUSG00000040703	-1.289927757	0.001861189
Ly86	ENSMUSG00000021423	1.055793438	0.001863752
Trmt5	ENSMUSG00000034442	-0.825044489	0.001863752
Zfp629	ENSMUSG00000045639	-0.911068747	0.001869049

Zyx	ENSMUSG00000029860	0.718979003	0.001870915
Tradd	ENSMUSG00000031887	0.997567222	0.001883133
Eci1	ENSMUSG00000024132	-0.722105699	0.001883377
Rrad	ENSMUSG00000031880	-0.637706297	0.001889633
Smc2	ENSMUSG00000028312	1.282853187	0.001894302
Slc25a3	ENSMUSG00000061904	-0.602015936	0.001915712
Gnl1	ENSMUSG00000024429	0.62225594	0.001923785
Emp3	ENSMUSG00000040212	0.830059461	0.00192562
Gpt2	ENSMUSG00000031700	-0.723568967	0.001929695
Thap6	ENSMUSG00000102644	-1.327896738	0.001945911
Pgam1-ps2	ENSMUSG00000082016	2.183906958	0.001951456
Sema6b	ENSMUSG00000001227	0.861811489	0.00196925
Zkscan8	ENSMUSG00000063894	-1.116799601	0.001983357
Rps12	ENSMUSG00000061983	1.026744527	0.001985338
Sirt3	ENSMUSG00000025486	-0.647224748	0.001985338
Xdh	ENSMUSG00000024066	-0.651778291	0.001985839
Shb	ENSMUSG00000044813	0.729168312	0.001990018
Rragd	ENSMUSG00000028278	-0.747731276	0.001993467
Acsl1	ENSMUSG00000018796	-0.836126132	0.001998062
Rps7	ENSMUSG00000061477	0.652799475	0.002005614
Rpl34	ENSMUSG00000062006	0.727772661	0.002005614
Gpam	ENSMUSG00000024978	-0.929170103	0.002017704
Ap1s2	ENSMUSG00000031367	0.634048276	0.002020357
Eprn	ENSMUSG00000113346	-1.794791492	0.002025207
Spata6	ENSMUSG00000034401	0.879653147	0.002026227
Cox7a1	ENSMUSG00000074218	-0.939287595	0.002029939
Gm9844	ENSMUSG00000091955	1.538156339	0.002049078
Cd34	ENSMUSG00000016494	0.587703498	0.002049303
Gm15501	ENSMUSG00000087412	0.726881626	0.002051413
Prim1	ENSMUSG00000025395	1.348668381	0.002064841
Scmh1	ENSMUSG00000000085	-0.697036385	0.002079048
Ap1s1	ENSMUSG00000004849	0.615824164	0.002079369
Dynll2	ENSMUSG00000020483	-0.686987124	0.002079369
Ccdc9b	ENSMUSG00000045838	-0.96770377	0.002086304
Gm17546	ENSMUSG00000078648	-1.990180372	0.002086877
Erh	ENSMUSG00000021131	0.708913817	0.002097252
Trmt2b	ENSMUSG00000067369	-0.653642941	0.002115732
Gm47283	ENSMUSG00000096768	-6.076730265	0.002116652
Zer1	ENSMUSG00000039686	-0.836470188	0.002116652
Lhfpl1	ENSMUSG00000041700	5.861889102	0.002116652

Lrp2	ENSMUSG00000027070	-2.303695027	0.002119482
Cltb	ENSMUSG00000047547	0.598308564	0.002122778
Slc25a20	ENSMUSG00000032602	-0.674705771	0.002123753
Fgl2	ENSMUSG00000039899	0.577212297	0.002124224
Dut	ENSMUSG00000027203	-0.718758779	0.002137674
Tgif1	ENSMUSG00000047407	1.195069755	0.002137837
Rapgef4	ENSMUSG00000049044	-1.008768675	0.00215378
Nipal3	ENSMUSG00000028803	-0.831006592	0.002183161
Tmem196	ENSMUSG00000048004	-2.372735657	0.002183161
Kif1a	ENSMUSG00000014602	2.541711178	0.002183161
Sbk3	ENSMUSG00000085272	-1.625271441	0.002185307
Gm2000	ENSMUSG00000078193	0.79257874	0.002200016
Abca6	ENSMUSG00000044749	-0.967211846	0.002200016
Lgi1	ENSMUSG00000067242	-2.516412026	0.002200016
Ephx2	ENSMUSG00000022040	-0.653913501	0.002210673
Taf6	ENSMUSG00000036980	-0.895535212	0.002218709
Nampt	ENSMUSG00000020572	-0.680564408	0.002221752
Rps20	ENSMUSG00000028234	0.659399487	0.002222301
Sp4	ENSMUSG00000025323	-1.089970863	0.002222301
Cyp26b1	ENSMUSG00000063415	-1.669909769	0.002222301
Mfge8	ENSMUSG00000030605	0.576512973	0.002233703
Gmfb	ENSMUSG00000062014	0.634404322	0.002242998
Lrba	ENSMUSG00000028080	-0.880303571	0.002249425
Cbfa2t3	ENSMUSG00000006362	-0.913145609	0.002250058
Saal1	ENSMUSG00000006763	0.902262184	0.002262025
Farp2	ENSMUSG00000034066	-1.152976549	0.002266619
Cd36	ENSMUSG00000002944	-0.671464692	0.002287718
Myl2	ENSMUSG00000013936	-0.525073877	0.002287718
Hspa1l	ENSMUSG00000007033	1.700556509	0.002290605
Ptgs2	ENSMUSG00000032487	1.116537278	0.002298677
Mccc1os	ENSMUSG00000086392	-2.140296998	0.00230257
Uba5	ENSMUSG00000032557	0.692458709	0.002305853
Nlrc3	ENSMUSG00000049871	1.925572097	0.00232624
Ntn1	ENSMUSG00000020902	-0.735611668	0.00233256
Pgd	ENSMUSG00000028961	0.672818199	0.002332651
Cand2	ENSMUSG00000030319	-0.786603841	0.002336308
Slc12a4	ENSMUSG00000017765	0.813313825	0.002337852
Dand5	ENSMUSG00000053226	-0.842267129	0.002344365
Fignl1	ENSMUSG00000035455	1.844538974	0.00235806
Sh3gl1	ENSMUSG00000003200	0.769437879	0.002358435

Myo1c	ENSMUSG00000017774	0.69878051	0.002367987
Tmem200b	ENSMUSG00000070720	1.229679436	0.002367987
Cygb	ENSMUSG00000020810	0.641698319	0.002374286
Tmem70	ENSMUSG00000025940	-0.677459018	0.002388054
Mkks	ENSMUSG00000027274	-0.942979742	0.002389891
C8g	ENSMUSG00000015083	-1.80860401	0.002392186
Mrps10	ENSMUSG00000034729	0.596376422	0.002398463
Satb1	ENSMUSG00000023927	-0.898144977	0.002398747
Npepl1	ENSMUSG00000039263	-0.564678427	0.0024024
Nuak2	ENSMUSG00000009772	1.257464784	0.002403572
Pde1b	ENSMUSG00000022489	1.890352048	0.002405587
Cideb	ENSMUSG00000022219	-2.893598251	0.002408282
Lgals9	ENSMUSG00000001123	0.739898546	0.002408282
Mbd6	ENSMUSG00000025409	-0.990137717	0.002408282
Id1	ENSMUSG00000042745	0.802327504	0.002412418
Uhrf1	ENSMUSG00000001228	1.374034061	0.002414734
Jpt2	ENSMUSG00000024165	0.902411988	0.002418844
Foxred1	ENSMUSG00000039048	-0.648683981	0.002421987
Rfc5	ENSMUSG00000029363	0.90224709	0.002424242
Nbr1	ENSMUSG00000017119	-0.594668409	0.002433278
Rnf19a	ENSMUSG00000022280	0.77601696	0.002442996
Slc25a28	ENSMUSG00000040414	-0.705826928	0.002445754
Vps29	ENSMUSG00000029462	0.661036779	0.002449701
Chmp3	ENSMUSG00000053119	0.584646999	0.002456269
Cercam	ENSMUSG00000039787	1.150387066	0.002462725
Tnrc6b	ENSMUSG00000047888	-1.017389341	0.002462725
Ndufb4c	ENSMUSG00000083380	-1.061760721	0.002476895
7-Sep	ENSMUSG00000001833	0.518673879	0.002480469
Zfyve21	ENSMUSG00000021286	-0.644087808	0.002488562
Bhlhe41	ENSMUSG00000030256	0.657559755	0.002492766
Dennd4c	ENSMUSG00000038024	-0.740837038	0.002493285
Gja4	ENSMUSG00000050234	0.961294147	0.002494558
Pnpla2	ENSMUSG00000025509	-0.543952085	0.002501241
Meis2	ENSMUSG00000027210	-0.831665411	0.002505688
Ctu2	ENSMUSG00000049482	0.868083514	0.002505688
Mgrn1	ENSMUSG00000022517	-0.716366804	0.002519504
Kif28	ENSMUSG00000087236	-2.661932769	0.002536564
Gtf2f2	ENSMUSG00000067995	0.699662951	0.002538433
Nap1l1	ENSMUSG00000058799	0.580426366	0.002560147
Angptl3	ENSMUSG00000028553	-1.817242854	0.002561912

Hint2	ENSMUSG00000028470	-0.614609458	0.002570915
Macrod1	ENSMUSG00000036278	-0.618559554	0.002571579
Pxdc1	ENSMUSG00000021411	0.877414167	0.002578338
Cd300a	ENSMUSG00000034652	1.38018485	0.002586112
Dsel	ENSMUSG00000038702	0.983090523	0.002586112
Acsl6	ENSMUSG00000020333	-1.030658687	0.002597315
Gpsm2	ENSMUSG00000027883	1.339995394	0.002607488
Gimap4	ENSMUSG00000054435	0.862515533	0.002612785
N4bp2l2	ENSMUSG00000029655	0.671136586	0.002614243
Cd44	ENSMUSG00000005087	0.942239387	0.002614243
B4galt4	ENSMUSG00000022793	0.878448874	0.002614407
Bhlhb9	ENSMUSG00000072964	-0.789856577	0.002617111
Mreg	ENSMUSG00000039395	-0.879418851	0.002626441
Mkrn2os	ENSMUSG00000068011	-2.51711784	0.002626441
Ccl2	ENSMUSG00000035385	1.027428989	0.002626441
St3gal3	ENSMUSG00000028538	-0.723494547	0.002626754
AU040320	ENSMUSG00000028830	-0.704948246	0.002647908
Atp5b	ENSMUSG00000025393	-0.525832091	0.002647908
Kyat1	ENSMUSG00000039648	-0.78581802	0.00266313
Dcaf8	ENSMUSG00000026554	-0.636859459	0.002665026
Tcf4	ENSMUSG00000053477	0.647135673	0.002665758
Slc25a25	ENSMUSG00000026819	1.048423005	0.002670104
Gpr22	ENSMUSG00000044067	-1.992601061	0.002670104
Rangap1	ENSMUSG00000022391	0.700664754	0.002670104
Gm3235	ENSMUSG00000090778	-2.528914274	0.002670593
AV026068	ENSMUSG00000100510	-0.880990653	0.002685907
Relb	ENSMUSG00000002983	0.780278795	0.002697788
Plod2	ENSMUSG00000032374	0.799365892	0.002704598
Fem1b	ENSMUSG00000032244	0.902717319	0.002717621
Gm9800	ENSMUSG00000045799	1.10698583	0.002738865
Myh9	ENSMUSG00000022443	0.64300181	0.002745936
Ndufs2	ENSMUSG00000013593	-0.558339312	0.002748805
Spns2	ENSMUSG00000040447	0.827011682	0.002750106
C1ra	ENSMUSG00000055172	-0.73286358	0.00275581
Ndufa10	ENSMUSG00000026260	-0.547086388	0.002785636
Snn	ENSMUSG00000037972	-0.789255016	0.002785636
Irf8	ENSMUSG00000041515	0.971954712	0.002785636
Rita1	ENSMUSG00000029600	-0.924822459	0.002785772
Pmp22	ENSMUSG00000018217	0.61420547	0.002807008
Tns2	ENSMUSG00000037003	-0.555283952	0.002823849

Prps2	ENSMUSG00000025742	-0.804910181	0.002845146
Rpn1	ENSMUSG00000030062	0.643621309	0.002846216
B3galt2	ENSMUSG00000033849	-1.549839842	0.002846216
Sptb	ENSMUSG00000021061	-1.043557601	0.002846216
Snx20	ENSMUSG00000031662	1.128108867	0.002850234
Klf13	ENSMUSG00000052040	0.74002551	0.00285071
Map2k1	ENSMUSG00000004936	0.594160495	0.002856468
Cd3d	ENSMUSG00000032094	2.970581548	0.002856468
Aurka	ENSMUSG00000027496	1.861641348	0.002856468
Spcs3	ENSMUSG00000054408	0.636510183	0.002862807
Kcnn1	ENSMUSG00000002908	-0.915713682	0.002862807
Ak3	ENSMUSG00000024782	-0.607231348	0.002874197
Trpm4	ENSMUSG00000038260	-0.852970292	0.002881921
Irf7	ENSMUSG00000025498	0.76468808	0.002885573
Hspb1	ENSMUSG00000004951	0.765765914	0.002885573
Pgap2	ENSMUSG00000030990	-0.664998873	0.002926396
Tarsl2	ENSMUSG00000030515	-0.810902558	0.002927517
Ppil6	ENSMUSG00000078451	-2.827536882	0.002928909
Zfp316	ENSMUSG00000046658	-1.147489144	0.002934434
Ppif	ENSMUSG00000021868	-0.71609031	0.002951806
Gm6560	ENSMUSG00000104913	0.955682602	0.00295204
Ubl7	ENSMUSG00000055720	-0.616403438	0.002954105
Dmwd	ENSMUSG00000030410	-0.615859582	0.002954105
2310022B05Rik	ENSMUSG00000031983	0.727743133	0.002954105
Zadh2	ENSMUSG00000049090	-0.605164328	0.002954105
Zbtb18	ENSMUSG00000063659	-0.800064865	0.002963407
Ccdc85c	ENSMUSG00000084883	-0.90597151	0.002967888
Serpina3f	ENSMUSG00000066363	2.506237283	0.002973014
Pla2g4a	ENSMUSG00000056220	0.747848929	0.002973014
Akr1a1	ENSMUSG00000028692	0.586240373	0.002973014
Jaml	ENSMUSG00000048534	2.775926587	0.002983353
Drosha	ENSMUSG00000022191	-0.854115576	0.003017927
Chd3	ENSMUSG00000018474	-0.902283471	0.003019825
Stum	ENSMUSG00000053963	-4.093057376	0.003026888
Atp8b2	ENSMUSG00000060671	0.795267492	0.003042604
Asb4	ENSMUSG00000042607	-1.437159488	0.003061553
Ankhd1	ENSMUSG00000024483	-0.799756169	0.003061553
2310057M21Rik	ENSMUSG00000040177	0.80053733	0.003071231
Zswim9	ENSMUSG00000070814	-1.019703929	0.003076087
Hadha	ENSMUSG00000025745	-0.674427217	0.003076097

Gm13657	ENSMUSG00000086813	-4.699951353	0.003076097
Ptger4	ENSMUSG00000039942	1.040402077	0.00308784
Tc2n	ENSMUSG00000021187	2.120064215	0.003092813
Trp53inp1	ENSMUSG00000028211	0.800318199	0.00310038
Pygb	ENSMUSG00000033059	-0.604198526	0.00310038
Wipf1	ENSMUSG00000075284	0.665643175	0.003112066
Kif20a	ENSMUSG00000003779	1.77008959	0.003113464
Fam213a	ENSMUSG00000021792	-0.6596064	0.003118956
Taf1d	ENSMUSG00000031939	0.880237321	0.003119632
Cox6b2	ENSMUSG00000051811	-1.925903835	0.003120276
Foxn2	ENSMUSG00000034998	0.848629699	0.003120276
Nrbp2	ENSMUSG00000075590	-0.898941657	0.003122644
Gck	ENSMUSG00000041798	1.047031838	0.003122644
Eif4h	ENSMUSG00000040731	0.539041285	0.00313531
Irx2	ENSMUSG00000001504	-1.507028682	0.00313531
Slf1	ENSMUSG00000021597	-0.759199155	0.003146814
Smc6	ENSMUSG00000020608	0.593397489	0.003150015
Eef2k	ENSMUSG00000035064	-0.792999891	0.003150015
Fbl	ENSMUSG00000046865	0.678442432	0.003151759
Cbr3	ENSMUSG00000022947	0.934842325	0.003153321
Gpr39	ENSMUSG00000026343	2.498199568	0.003153321
Grm1	ENSMUSG00000019828	-1.063839059	0.003155197
Hdac5	ENSMUSG00000008855	-0.668596774	0.003160994
Ccr1	ENSMUSG00000025804	0.966966684	0.003160994
Cyb5rl	ENSMUSG00000028621	-0.965694391	0.003166226
Nmd3	ENSMUSG00000027787	0.652533919	0.003173732
9530026P05Rik	ENSMUSG00000097462	-1.368439382	0.003173732
Hspa4	ENSMUSG00000020361	0.543192207	0.003173732
Ndufs6b	ENSMUSG00000083820	-1.610544359	0.003202331
Calm2	ENSMUSG00000036438	0.655288228	0.003207134
Ror1	ENSMUSG00000035305	1.131757796	0.0032191
Slc7a2	ENSMUSG00000031596	-1.252161865	0.003220434
Ncor1	ENSMUSG00000018501	-0.713497532	0.003220971
Tmem205	ENSMUSG00000040883	-0.552049953	0.003241935
Ngfr	ENSMUSG00000000120	3.720519955	0.00325022
Syde2	ENSMUSG00000036863	-0.843080146	0.00325022
Gpi1	ENSMUSG00000036427	0.770648755	0.003259101
Cacna2d1	ENSMUSG00000040118	-0.666566445	0.00326894
Mlycd	ENSMUSG00000074064	-0.643552846	0.003270742
Gdi2	ENSMUSG00000021218	0.675763977	0.003270742

Srpx	ENSMUSG00000090084	0.753897572	0.003283687
Ybx2	ENSMUSG00000018554	-1.059356119	0.003283687
Rab7b	ENSMUSG00000052688	0.829085344	0.003309785
Cdkn2aipnl	ENSMUSG00000020392	0.69297469	0.003312459
Rala	ENSMUSG00000008859	0.595025508	0.003322442
Tmed5	ENSMUSG00000063406	0.704414842	0.003384498
C1s1	ENSMUSG00000038521	-0.641052424	0.00339156
Ppp1r26	ENSMUSG00000035829	-2.216008644	0.00339156
Zfp395	ENSMUSG00000034522	-0.962718982	0.003401664
Elk3	ENSMUSG00000008398	0.70104324	0.003411333
Nipsnap3b	ENSMUSG00000015247	0.894078343	0.003411333
Ktn1	ENSMUSG00000021843	-0.670762089	0.003411333
Alg5	ENSMUSG00000036632	0.718121418	0.003414717
Notch1	ENSMUSG00000026923	-0.869547385	0.003415204
Kif23	ENSMUSG00000032254	1.716724215	0.003415204
Polr1a	ENSMUSG00000049553	-0.770515347	0.003418353
Slc37a1	ENSMUSG00000024036	-1.321606755	0.003426712
Bin3	ENSMUSG00000022089	0.891417257	0.003431868
Ogfod3	ENSMUSG00000025169	-0.849881545	0.003436405
Cd300lf	ENSMUSG00000047798	5.238593085	0.0034452
Limd2	ENSMUSG00000040699	0.795047175	0.00344854
Mfsd4b3	ENSMUSG00000071335	-1.164694055	0.003457317
Cdca4	ENSMUSG00000047832	0.889615643	0.003463864
Gm28651	ENSMUSG00000101086	-1.472064326	0.003465574
Gjb5	ENSMUSG00000042357	2.551709388	0.003465574
6430571L13Rik	ENSMUSG00000037977	-1.445538197	0.00347465
Hsdl2	ENSMUSG00000028383	-0.728112615	0.00347465
6-Mar	ENSMUSG00000039100	-0.650260328	0.003486939
Lnpep	ENSMUSG00000023845	-0.637981276	0.00349133
Gm15427	ENSMUSG00000081051	0.620913952	0.00349133
Rce1	ENSMUSG00000024889	0.846963911	0.003511022
Sema7a	ENSMUSG00000038264	0.920810597	0.003511075
Lrrc4b	ENSMUSG00000047085	-1.216200591	0.003511075
Nr0b2	ENSMUSG00000037583	-0.974945464	0.003511075
Gm13111	ENSMUSG00000085069	-1.856714245	0.003531107
AC167229.1	ENSMUSG00000112831	-2.081079547	0.003531501
Fcgr2b	ENSMUSG00000026656	0.745441136	0.003537927
Clic5	ENSMUSG00000023959	0.740864518	0.003544277
Klhl6	ENSMUSG00000043008	0.822718946	0.003544277
Tmem220	ENSMUSG00000050270	-1.403248016	0.003546034

Fam214a	ENSMUSG00000034858	-0.878819193	0.003546856
Tmem167	ENSMUSG00000012422	0.771866111	0.00357189
Zfp945	ENSMUSG00000059142	-0.735412068	0.003577074
Cbr1	ENSMUSG00000051483	-0.89774786	0.003590009
Nr1d2	ENSMUSG00000021775	-0.573750124	0.003594079
Zpr1	ENSMUSG00000032078	0.667528355	0.003595347
5-Sep	ENSMUSG00000072214	1.526831656	0.003597708
Hr	ENSMUSG00000022096	0.778346828	0.003598475
Cbx6	ENSMUSG00000089715	0.642377859	0.003598811
Gm31013	ENSMUSG00000111867	-0.867539086	0.003601992
Emilin2	ENSMUSG00000024053	-0.838873204	0.003621284
Auts2	ENSMUSG00000029673	-1.005016367	0.003634031
Cebpa	ENSMUSG00000034957	0.826652877	0.003634031
Cars2	ENSMUSG00000056228	-0.765333517	0.003639244
4430402I18Rik	ENSMUSG00000064202	-0.755394458	0.003657679
Rnf4	ENSMUSG00000029110	0.60350587	0.003657679
Rnf150	ENSMUSG00000047747	-0.78762828	0.003667245
Echdc2	ENSMUSG00000028601	-0.795687974	0.003686224
Gm3534	ENSMUSG00000098192	3.278611619	0.003704774
Ubr3	ENSMUSG00000044308	-0.634234937	0.003709677
Itgb5	ENSMUSG00000022817	0.766845562	0.003730181
Gatb	ENSMUSG00000028085	-0.639822266	0.00374
Fndc1	ENSMUSG00000071984	0.882765996	0.00374
Ncoa7	ENSMUSG00000039697	0.913762547	0.00376037
Gpr157	ENSMUSG00000047875	-0.835013273	0.003767616
0610009L18Rik	ENSMUSG00000043644	-0.951569832	0.003771605
Ttc32	ENSMUSG00000066637	-0.923477456	0.00378643
Pfn1	ENSMUSG00000018293	0.559675849	0.003810488
Car9	ENSMUSG00000028463	2.402779962	0.003815206
Ltbr	ENSMUSG00000030339	0.634162161	0.003820593
Ppp1r3c	ENSMUSG00000067279	0.806111308	0.003828167
Rian	ENSMUSG00000097451	0.859832426	0.003830755
Bod1l	ENSMUSG00000061755	-0.797311853	0.003838457
Gm11992	ENSMUSG00000040978	-3.022827174	0.003841043
Ifi47	ENSMUSG00000078920	0.905860668	0.003845771
Rpsa-ps2	ENSMUSG00000045055	0.796720417	0.003849678
Sptlc2	ENSMUSG00000021036	0.661308341	0.003852946
Cxadr	ENSMUSG00000022865	-0.785676425	0.003894598
Ercc1	ENSMUSG00000003549	0.642654027	0.003907027
Far1	ENSMUSG00000030759	0.620752417	0.003908936

Gm5854	ENSMUSG00000115584	0.630072345	0.003910278
Gm42047	ENSMUSG00000110631	1.930821456	0.003923544
Ptgfrn	ENSMUSG00000027864	0.65860032	0.003932084
Fam84b	ENSMUSG00000072568	-1.125051365	0.003936782
1700003E16Rik	ENSMUSG00000030030	-2.234347202	0.003953473
Maea	ENSMUSG00000079562	0.546903102	0.003953473
Dip2c	ENSMUSG00000048264	-0.774753827	0.003953473
Gmppb	ENSMUSG00000070284	0.866347638	0.003955773
Mal	ENSMUSG00000027375	-0.642041567	0.003960421
Dppa3	ENSMUSG00000046323	5.274626978	0.00397786
Ezh2	ENSMUSG00000029687	0.932326989	0.003981122
Cd209a	ENSMUSG00000031494	2.058888874	0.004004735
Ak1	ENSMUSG00000026817	-0.582917708	0.004017315
Itgb1	ENSMUSG00000025809	0.488362424	0.004025814
Cd300c2	ENSMUSG00000044811	1.319702101	0.004027853
Trap1	ENSMUSG00000005981	-0.593385139	0.004036192
Tnip3	ENSMUSG00000044162	-1.535581677	0.004059445
Pde1a	ENSMUSG00000059173	0.801606434	0.004063145
Sod2	ENSMUSG00000006818	-0.665886336	0.004063954
Khk	ENSMUSG00000029162	-0.698405657	0.004070071
Cap1	ENSMUSG00000028656	0.67723658	0.004070442
Snapin	ENSMUSG00000001018	-0.542448439	0.004084487
Rrm2	ENSMUSG00000020649	1.468394248	0.00409024
Zbtb20	ENSMUSG00000022708	-0.75396152	0.004095784
Mlkl	ENSMUSG00000012519	1.06522768	0.004097495
1-Mar	ENSMUSG00000036469	1.153767599	0.004106013
Cttnbp2nl	ENSMUSG00000062127	0.834168199	0.004106013
Gorasp1	ENSMUSG00000032513	-0.720167882	0.004123399
Ahdc1	ENSMUSG00000037692	-0.858811276	0.004126778
A930024E05Rik	ENSMUSG00000056735	-5.698305304	0.004141511
Dcbld2	ENSMUSG00000035107	0.846280388	0.004150682
Slc5a6	ENSMUSG00000006641	-1.107628919	0.004217673
Rxra	ENSMUSG00000015846	-0.650320552	0.004223829
Rap1a	ENSMUSG00000068798	0.520844146	0.004224772
Prpf8	ENSMUSG00000020850	-0.637126797	0.004237121
Hnrnpa3	ENSMUSG00000059005	0.62356381	0.004244831
Cct3	ENSMUSG00000001416	0.579549275	0.004249443
Mmachc	ENSMUSG00000028690	-0.689461362	0.004273267
Rhobtb2	ENSMUSG00000022075	-0.873931533	0.004288472
Cnot1	ENSMUSG00000036550	-0.739226081	0.004297913

Isoc1	ENSMUSG00000024601	-0.731745574	0.00432728
Kmt2d	ENSMUSG00000048154	-0.910250029	0.004350276
Calm3	ENSMUSG00000019370	0.545273691	0.004368399
Cenpv	ENSMUSG00000018509	-0.834867958	0.004383368
Ssu72	ENSMUSG00000029038	0.571796039	0.004385991
Pnp	ENSMUSG00000115338	0.8672078	0.004394112
Zfp799	ENSMUSG00000095253	-1.062540156	0.004417087
Ctnnbip1	ENSMUSG00000028988	0.730158233	0.004417286
Sord	ENSMUSG00000027227	-0.756934778	0.004422655
Hras	ENSMUSG00000025499	0.536983157	0.004473873
Nelfe	ENSMUSG00000024369	0.670096672	0.004488581
Ndrg2	ENSMUSG00000004558	-0.533208625	0.00450352
AA467197	ENSMUSG00000033213	2.709333127	0.004518369
Eif3c	ENSMUSG00000030738	0.741262942	0.00452462
Psrc1	ENSMUSG00000068744	1.770772634	0.004552533
Aacs	ENSMUSG00000029482	1.110687711	0.004565371
Lfng	ENSMUSG00000029570	0.753391212	0.004565592
Limch1	ENSMUSG00000037736	-0.631537003	0.004590165
Tmem255b	ENSMUSG00000038457	1.60750951	0.004600202
Ankrd13a	ENSMUSG00000041870	0.625998517	0.004626872
Rpl15	ENSMUSG00000012405	0.544855195	0.004629369
Gm10131	ENSMUSG00000063412	0.80649505	0.004629369
Ryk	ENSMUSG00000032547	0.56992596	0.004641149
Ppp1r3b	ENSMUSG00000046794	-0.935935453	0.004646372
Scn10a	ENSMUSG00000034533	-2.257338184	0.004646372
Zrsr1	ENSMUSG00000044068	-0.781938403	0.004650256
Xpnpep1	ENSMUSG00000025027	0.560214148	0.004651023
Kifc2	ENSMUSG00000004187	-1.557657202	0.004651559
Colgalt1	ENSMUSG00000034807	0.585283728	0.004666913
Skida1	ENSMUSG00000054074	-1.407962674	0.00466899
Rgs2	ENSMUSG00000026360	-0.666607138	0.004681646
Padi4	ENSMUSG00000025330	1.797353374	0.004686204
Dsc2	ENSMUSG00000024331	-0.768582285	0.004702353
Apobec1	ENSMUSG00000040613	0.883474547	0.004711014
Eif5a2	ENSMUSG00000050192	1.10857567	0.004711014
Apobec2	ENSMUSG00000040694	-0.563906794	0.004726189
Aif1	ENSMUSG00000024397	0.944408676	0.004734904
Cdc14b	ENSMUSG00000033102	-1.06571136	0.004734956
Abhd15	ENSMUSG00000000686	2.592718868	0.004742822
Eprs	ENSMUSG00000026615	0.77552771	0.004758736

Ptpn22	ENSMUSG00000027843	1.823334814	0.004772866
Olfr1033	ENSMUSG00000045392	0.803016119	0.00480492
P4ha3	ENSMUSG00000051048	2.583338727	0.00481093
Homer3	ENSMUSG00000003573	0.971735643	0.004817044
Mak16	ENSMUSG00000031578	0.82504581	0.004821677
Tmem29	ENSMUSG00000041353	1.131785692	0.004828964
Ssrp1	ENSMUSG00000027067	0.558798858	0.004828964
Disp1	ENSMUSG00000030768	-1.017601401	0.004831123
Abi1	ENSMUSG00000058835	0.622314328	0.004853936
Clcn1	ENSMUSG00000029862	-2.786956115	0.004860037
Tipin	ENSMUSG00000032397	0.755951105	0.004875321
Gm10288	ENSMUSG00000070343	0.910852401	0.004881469
Znhit2	ENSMUSG00000075227	0.661630789	0.004894527
Csnk1g3	ENSMUSG00000073563	0.695832115	0.004916684
Hmcn1	ENSMUSG00000066842	-0.837633916	0.004916684
Dph5	ENSMUSG00000033554	0.861314305	0.004931142
Ablim2	ENSMUSG00000029095	-0.905519313	0.004952149
Pitpnm3	ENSMUSG00000040543	-1.994775613	0.004963089
Tra2b	ENSMUSG00000022858	0.530220878	0.004965518
Wdr6	ENSMUSG00000066357	-0.876320324	0.004965518
Anxa4	ENSMUSG00000029994	0.665256133	0.004965518
Kcnd2	ENSMUSG00000060882	-1.286265894	0.004976138
2610035D17Rik	ENSMUSG00000087259	-1.001252504	0.004991573
Pcmtd1	ENSMUSG00000051285	-0.58043536	0.004992425
Ftl1-ps1	ENSMUSG00000062382	0.883672352	0.005008934
Tmem200a	ENSMUSG00000049420	4.617378486	0.005018731
Il7r	ENSMUSG00000003882	2.415570084	0.005038911
Rhou	ENSMUSG00000039960	0.784410957	0.005040307
Dlgap5	ENSMUSG00000037544	2.004841275	0.005051678
Fryl	ENSMUSG00000070733	-0.835964992	0.005057773
Bcl9	ENSMUSG00000038256	-1.131339155	0.005057773
C9orf72	ENSMUSG00000028300	-0.682278331	0.005057773
Slc22a4	ENSMUSG00000020334	1.320768109	0.005066059
Esyt3	ENSMUSG00000037681	-4.006171285	0.005068225
Kmt2b	ENSMUSG00000006307	-0.941202836	0.005078747
Mybl2	ENSMUSG00000017861	2.592778384	0.005101587
Klf15	ENSMUSG00000030087	-0.823413157	0.005109729
Il15ra	ENSMUSG00000023206	-0.900103667	0.005123705
Renbp	ENSMUSG00000031387	0.918364761	0.005123705
Lysmd4	ENSMUSG00000043831	-0.772372832	0.005179254

Syt3	ENSMUSG00000030731	-1.520093842	0.005201459
Tmem245	ENSMUSG00000055296	-0.66036351	0.005221585
Ubxn10	ENSMUSG00000043621	-1.176465842	0.00526583
Mtus2	ENSMUSG00000029651	-0.605054134	0.00529478
Shank3	ENSMUSG00000022623	-0.734170234	0.005326592
Smarcc2	ENSMUSG00000025369	-0.573877841	0.005335009
Mblac2	ENSMUSG00000051098	-1.195645493	0.005335009
Llph	ENSMUSG00000020224	0.737677671	0.005365723
Tro	ENSMUSG00000025272	2.005697975	0.005370355
Myef2	ENSMUSG00000027201	1.082738665	0.005370355
BE692007	ENSMUSG00000099757	1.258795554	0.005370355
Helz	ENSMUSG00000020721	-0.977658033	0.005400998
Pvalb	ENSMUSG00000005716	-1.88549207	0.005408057
Hmgn1	ENSMUSG00000040681	0.592264829	0.005410004
Gm45145	ENSMUSG00000109076	-5.663341332	0.005424578
Idh3b	ENSMUSG00000027406	-0.540038214	0.005445884
Hck	ENSMUSG00000003283	1.023973591	0.005451755
Zmym3	ENSMUSG00000031310	-0.881878461	0.005461618
Hells	ENSMUSG00000025001	1.82211572	0.005473298
Psmb1	ENSMUSG00000014769	0.486497526	0.005473298
Hnrnpab	ENSMUSG00000020358	0.543143585	0.005484754
Mif4gd	ENSMUSG00000020743	-0.639853353	0.00548887
Cdca7	ENSMUSG00000055612	1.699616262	0.005496889
Katna1	ENSMUSG00000019794	0.717670256	0.005516006
Tmem136	ENSMUSG00000048503	-0.884634476	0.005516006
Zfand1	ENSMUSG00000039795	-0.663088645	0.005520633
Plat	ENSMUSG00000031538	0.688011386	0.005520633
Slc35b1	ENSMUSG00000020873	0.602521026	0.005520633
Papd4	ENSMUSG00000042167	0.686878174	0.005520633
Slc38a3	ENSMUSG00000010064	-1.025086223	0.005520633
Psmc4	ENSMUSG00000030603	0.514310147	0.005553084
Ndufs6	ENSMUSG00000021606	-0.719557916	0.005553084
Rpl13	ENSMUSG00000000740	0.538123403	0.005553189
Gimap6	ENSMUSG00000047867	0.630457958	0.005553189
Aard	ENSMUSG00000068522	2.199867342	0.005586469
Bsg	ENSMUSG00000023175	0.572189914	0.005594518
Exosc5	ENSMUSG00000061286	0.649714111	0.005616232
Slc12a6	ENSMUSG00000027130	-0.80138767	0.005636047
Cacna1h	ENSMUSG00000024112	-2.65763994	0.005636047
Sec22b	ENSMUSG00000027879	0.581196394	0.005636047

Arpc3	ENSMUSG00000029465	0.575670572	0.005648406
Phactr2	ENSMUSG00000062866	-0.656603811	0.005660056
Meis1	ENSMUSG00000020160	-0.851497375	0.005660056
Cgref1	ENSMUSG00000029161	1.634468388	0.005691304
Oxa1l	ENSMUSG00000000959	-0.537743038	0.00570561
Rplp1	ENSMUSG00000007892	0.684312126	0.005742955
Gm26809	ENSMUSG00000097815	-0.81417895	0.005750241
Slc1a4	ENSMUSG00000020142	1.296822536	0.005750241
Ano1	ENSMUSG00000031075	-1.190579505	0.005754938
Flt3	ENSMUSG00000042817	3.060035376	0.005756678
Ptch1	ENSMUSG00000021466	-1.09422523	0.005759496
Tmem65	ENSMUSG00000062373	-0.698085951	0.005774318
Fkbp7	ENSMUSG00000002732	0.695996503	0.005774318
Qsox2	ENSMUSG00000036327	-0.974425441	0.005801269
C7	ENSMUSG00000079105	-1.018918054	0.005813932
Grip2	ENSMUSG00000030098	-1.366580254	0.005815973
Ywhab	ENSMUSG00000018326	0.505584145	0.005816425
Acsm5	ENSMUSG00000030972	-1.981329852	0.005825517
Gm32369	ENSMUSG00000112950	-3.398773238	0.005828046
Plpp1	ENSMUSG00000021759	0.606056616	0.005836198
Slc35e1	ENSMUSG00000019731	-0.669077498	0.005878457
Necap2	ENSMUSG00000028923	0.740245583	0.005891842
Adprhl1	ENSMUSG00000031448	-0.511074898	0.005893698
Prob1	ENSMUSG00000073600	-0.764624026	0.005893698
Psma3	ENSMUSG00000060073	0.515901614	0.005894275
Synj2	ENSMUSG00000023805	-0.771850899	0.005894736
Ramp1	ENSMUSG00000034353	-0.767957777	0.005894736
Gsta4	ENSMUSG00000032348	-0.67718351	0.005894736
Gipc1	ENSMUSG00000019433	0.570925974	0.005894736
Gpc1	ENSMUSG00000034220	-0.727045324	0.005894736
Rexo2	ENSMUSG00000032026	0.636351903	0.005899155
Fxyd6	ENSMUSG00000066705	0.797263878	0.005899155
Hist1h2be	ENSMUSG00000047246	-1.152908143	0.005901199
Paip2b	ENSMUSG00000045896	-0.605873816	0.005912936
Golga7	ENSMUSG00000015341	0.543121386	0.005921855
Zfp467	ENSMUSG00000068551	-0.982078525	0.005934775
Pgrmc1	ENSMUSG00000006373	0.582048958	0.005973289
Fam104a	ENSMUSG00000041629	0.644135035	0.005982103
Gm5526	ENSMUSG00000084817	1.623343023	0.005992546
Zfp106	ENSMUSG00000027288	-0.647685706	0.006007081

2310015D24Rik	ENSMUSG00000099411	-2.220607919	0.006052784
Aars	ENSMUSG00000031960	0.609630295	0.006072351
Foxo6os	ENSMUSG00000084929	-1.434490057	0.006074138
Ckm	ENSMUSG00000030399	-0.823862872	0.006075888
Arhgap27	ENSMUSG00000034255	0.903453391	0.006075888
Nudt7	ENSMUSG00000031767	-0.655387482	0.006099966
Cd53	ENSMUSG00000040747	0.782108628	0.006099966
Sema6c	ENSMUSG00000038777	-1.673585225	0.006099966
Wdr4	ENSMUSG00000024037	0.927380228	0.00610504
St3gal4	ENSMUSG00000032038	-0.56698539	0.006129731
Arfip2	ENSMUSG00000030881	0.701092401	0.006129731
Flna	ENSMUSG00000031328	0.548525569	0.006136498
Jph1	ENSMUSG00000042686	-0.746782505	0.00614671
Gm26862	ENSMUSG00000097644	-2.19914292	0.006154011
Gpt	ENSMUSG00000022546	-0.894723091	0.006200662
Cavin1	ENSMUSG00000004044	0.488503193	0.006208205
Cct2	ENSMUSG00000034024	0.544136198	0.006209588
Mfsd7a	ENSMUSG00000029490	-1.621448282	0.006264342
Gmfg	ENSMUSG00000060791	0.872239905	0.006270154
Adamts5	ENSMUSG00000022894	0.627706703	0.006309619
Rnf5	ENSMUSG00000015478	-0.983463983	0.006322553
Rps6-ps4	ENSMUSG00000081406	0.619004478	0.00637856
Mtmr2	ENSMUSG00000031918	0.615721594	0.006381594
Atpaf1	ENSMUSG00000028710	-0.749669943	0.006417932
Dio3os	ENSMUSG00000113581	2.512153443	0.006418474
Fam107b	ENSMUSG00000026655	0.995642468	0.006431355
Pi15	ENSMUSG00000067780	1.175034211	0.006435735
Pmpca	ENSMUSG00000026926	-0.556190448	0.006435735
Gm5559	ENSMUSG00000096617	0.812007893	0.006443829
Hsd17b12	ENSMUSG00000027195	0.597786073	0.006443829
Gzma	ENSMUSG00000023132	1.840431949	0.006460834
Clta	ENSMUSG00000028478	0.525121667	0.006473058
Npr2	ENSMUSG00000028469	-0.682872421	0.006473058
Fmnl2	ENSMUSG00000036053	0.788521997	0.006493274
Rxrg	ENSMUSG00000015843	-0.756278303	0.006507637
Pgm3	ENSMUSG00000056131	0.957860277	0.006531903
Rab27b	ENSMUSG00000024511	1.31863311	0.006573663
Rph3al	ENSMUSG00000020847	-1.056492531	0.006573828
Phf10	ENSMUSG00000023883	0.606489854	0.006598137
Kif20b	ENSMUSG00000024795	1.811233097	0.006598137

Ndufs1	ENSMUSG00000025968	-0.668109775	0.006625564
Gm6204	ENSMUSG00000105879	1.704554358	0.006625608
Gm26808	ENSMUSG00000097489	-3.065264357	0.006648908
Mgam	ENSMUSG00000068587	2.986691937	0.006648908
mt-Te	ENSMUSG00000064369	-2.398230333	0.006696684
Hist1h1c	ENSMUSG00000036181	0.49967768	0.006696684
Gm31600	ENSMUSG00000114800	-5.573975945	0.006749664
Cox15	ENSMUSG00000040018	-0.619662555	0.006751939
Pole3	ENSMUSG00000028394	0.56708642	0.0067648
Mtg2	ENSMUSG00000039069	-0.680671835	0.00676902
Junb	ENSMUSG00000052837	1.032488317	0.006769861
Rpl36a	ENSMUSG00000079435	0.782695457	0.006774353
Atg10	ENSMUSG00000021619	-0.65583478	0.006789282
Rps4x	ENSMUSG00000031320	0.543040332	0.006813331
Timm29	ENSMUSG00000048429	-0.587042656	0.006817853
Chaf1a	ENSMUSG00000002835	1.122465838	0.006817853
Gal3st2c	ENSMUSG00000073608	-2.012844455	0.006824558
Sox9	ENSMUSG00000000567	1.200639093	0.006832275
Trappc10	ENSMUSG00000000374	-0.639773365	0.006832275
Intu	ENSMUSG00000060798	-0.79989471	0.006838962
Daam1	ENSMUSG00000034574	-0.656154608	0.006841881
Kdm1b	ENSMUSG00000038080	-0.781709727	0.006877415
Etfrf1	ENSMUSG00000040370	-0.617464686	0.006877415
C1qb	ENSMUSG00000036905	0.70167719	0.006893697
Cavin4	ENSMUSG00000028348	0.671008769	0.006905496
Fam50a	ENSMUSG00000001962	0.597833347	0.006914282
Pkig	ENSMUSG00000035268	-0.5016203	0.006914282
Ptger3	ENSMUSG00000040016	3.2687732	0.006914282
Numbl	ENSMUSG00000063160	0.770447884	0.006921605
Ttll11	ENSMUSG00000026885	1.206696851	0.0069265
Unc45b	ENSMUSG00000018845	-0.828673619	0.0069265
Tpm3-rs7	ENSMUSG00000058126	0.892881088	0.006931887
Cyp4f18	ENSMUSG00000003484	1.656554182	0.006942844
Gm15640	ENSMUSG00000085781	5.056504377	0.006945209
Unc13b	ENSMUSG00000028456	-0.731253786	0.006945209
Klhl29	ENSMUSG00000020627	1.89145935	0.006965086
Vat1	ENSMUSG00000034993	0.770867853	0.006966144
Slc39a11	ENSMUSG00000041654	1.031449845	0.006966144
Asl	ENSMUSG00000025533	0.786817821	0.006971823
Amigo1	ENSMUSG00000050947	-0.803830785	0.006974583

Mgmt	ENSMUSG00000054612	-0.774909067	0.006974583
Rad51	ENSMUSG00000027323	1.622359639	0.006974583
Zfp568	ENSMUSG00000074221	0.853037443	0.006974583
Mef2a	ENSMUSG00000030557	-0.570347709	0.006974583
Gm13054	ENSMUSG00000086806	4.509951514	0.006977148
Immp1l	ENSMUSG00000042670	-0.541057983	0.00698552
Myo1b	ENSMUSG00000018417	0.829917613	0.006990612
Gm2962	ENSMUSG00000089838	-1.077158597	0.006990978
Haus3	ENSMUSG00000079555	0.93384849	0.006998369
Klf2	ENSMUSG00000055148	0.788373091	0.007045139
Gulp1	ENSMUSG00000056870	0.758271836	0.007097252
Pnpla7	ENSMUSG00000036833	-0.816152567	0.007098028
Gpr27	ENSMUSG00000072875	-1.001570624	0.007098028
Pink1	ENSMUSG00000028756	-0.52490653	0.007098028
Oip5	ENSMUSG00000072980	2.242919416	0.007117397
Jmjd8	ENSMUSG00000025736	-0.989863495	0.00711886
2200002D01Rik	ENSMUSG00000030587	1.46384017	0.007124612
1810044D09Rik	ENSMUSG00000100680	-1.323626878	0.007153799
Ogdhl	ENSMUSG00000021913	-1.675840034	0.007173038
Bcl9l	ENSMUSG00000063382	-0.823329589	0.007173038
Pde9a	ENSMUSG00000041119	1.008510929	0.007178322
Ptgr2	ENSMUSG00000072946	-0.555220549	0.007194196
Cry2	ENSMUSG00000068742	-0.60367143	0.007194247
Rab8a	ENSMUSG00000003037	0.547500173	0.007194247
Lrrc58	ENSMUSG00000034158	0.563875203	0.007220707
Nop58	ENSMUSG00000026020	0.690822779	0.007220707
Sirpb1c	ENSMUSG00000074677	2.547008864	0.007246235
Mtss1	ENSMUSG00000022353	-0.693449087	0.007256291
Fam120c	ENSMUSG00000025262	-1.242070625	0.007269487
Stx2	ENSMUSG00000029428	0.667616684	0.007300212
Ip6k3	ENSMUSG00000024210	1.064995841	0.00731545
Tprn	ENSMUSG00000048707	1.096188028	0.007322322
Adcy7	ENSMUSG00000031659	0.942306654	0.007328676
Tpp1	ENSMUSG00000030894	-0.620645365	0.007332666
Rcsd1	ENSMUSG00000040723	-0.543070911	0.007365783
Coro1b	ENSMUSG00000024835	0.560847432	0.007365865
Eid2b	ENSMUSG00000070705	-0.749314601	0.007405117
Nob1	ENSMUSG00000003848	0.628156499	0.007424949
Osbp	ENSMUSG00000024687	-0.567817203	0.007437955
Psap	ENSMUSG00000004207	-0.562754645	0.007485898

Adm2	ENSMUSG00000054136	3.674332713	0.007490254
Eno3	ENSMUSG00000060600	-0.753069191	0.007493951
Sbno2	ENSMUSG00000035673	0.793679815	0.00752484
Nectin3	ENSMUSG00000022656	0.826855932	0.007565321
Kctd5	ENSMUSG00000016946	0.712700938	0.007591967
Med12l	ENSMUSG00000056476	-1.643750911	0.007594156
Dnd1	ENSMUSG00000044595	-1.969332561	0.007618195
Magix	ENSMUSG00000031147	-1.096894057	0.007618195
Rgs10	ENSMUSG00000030844	0.770112536	0.007651015
Tbc1d10a	ENSMUSG00000034412	0.803472985	0.007670481
Pcyt2	ENSMUSG00000025137	-0.613250005	0.007676181
Tyro3	ENSMUSG00000027298	1.155630066	0.007682229
Rorc	ENSMUSG00000028150	-0.621776909	0.00770757
Sec62	ENSMUSG00000027706	0.6099118	0.00770757
Prr33	ENSMUSG00000043795	-1.431096077	0.00770757
Napsa	ENSMUSG00000002204	2.300920043	0.007715737
Galnt15	ENSMUSG00000021903	-0.597904	0.007715737
Chst4	ENSMUSG00000035930	-1.258466466	0.007716037
Stard10	ENSMUSG00000030688	-0.664529384	0.007716037
Mis18a	ENSMUSG00000022978	1.037666079	0.007750496
Fem1a	ENSMUSG00000043683	-0.667555157	0.007782264
Fbln1	ENSMUSG00000006369	-0.726965032	0.007807377
Comt	ENSMUSG00000000326	0.592258057	0.007829954
Gss	ENSMUSG00000027610	0.719031192	0.00783362
Mid1ip1	ENSMUSG00000008035	-1.631688835	0.00784239
Itsn1	ENSMUSG00000022957	-0.561348339	0.007845479
Hlcs	ENSMUSG00000040820	-0.979200647	0.007858749
Fbxo16	ENSMUSG00000034532	-3.128797365	0.007880314
Nmnat3	ENSMUSG00000032456	-0.680937592	0.007880314
Uso1	ENSMUSG00000029407	0.620070772	0.007881238
Smc4	ENSMUSG00000034349	0.60868852	0.00790911
Hfe	ENSMUSG00000006611	-0.548736197	0.007921432
Coq8b	ENSMUSG00000003762	0.900183849	0.007925972
Pole4	ENSMUSG00000030042	0.586361933	0.007925972
Rpl39	ENSMUSG00000079641	0.847394879	0.007935236
Kdm6b	ENSMUSG00000018476	-0.847106096	0.007943398
Casp12	ENSMUSG00000025887	0.695840337	0.007964554
Plscr5	ENSMUSG00000095654	-2.060379411	0.007974473
Sae1	ENSMUSG00000052833	0.557794673	0.007980153
Lrp3	ENSMUSG00000001802	-0.888254971	0.007991693

Gm5737	ENSMUSG00000109392	-4.397609163	0.008020452
Mlf1	ENSMUSG00000048416	-0.751969723	0.008026473
Dnttip2	ENSMUSG00000039756	0.535484146	0.008038574
Gm13375	ENSMUSG00000075514	-0.676109935	0.008046954
Lgals4	ENSMUSG00000053964	-1.510505434	0.008109252
Fam210a	ENSMUSG00000038121	-0.599978989	0.008126414
Rhd	ENSMUSG00000028825	-1.058202744	0.00813311
Egf	ENSMUSG00000028017	-1.767300999	0.008151352
Lxn	ENSMUSG00000047557	0.945288217	0.008173865
Casc1	ENSMUSG00000043541	-1.225598151	0.00821081
Stil	ENSMUSG00000028718	3.132507319	0.008212463
Gm49492	ENSMUSG00000116180	-1.787102868	0.008220275
Susd3	ENSMUSG00000021384	1.191916118	0.008220622
Copb1	ENSMUSG00000030754	0.537994042	0.008232922
Cyb5r2	ENSMUSG00000048065	-1.425382972	0.008252817
Tmem182	ENSMUSG00000079588	-0.590264636	0.008257782
Cdc42se1	ENSMUSG00000046722	0.612728375	0.008278635
Haus7	ENSMUSG00000031371	0.928029544	0.008278635
2610507I01Rik	ENSMUSG00000085882	-1.180864093	0.008284091
Hsd11b2	ENSMUSG00000031891	4.99318151	0.008293118
Fads6	ENSMUSG00000044788	-1.420266117	0.008303683
Ncf4	ENSMUSG00000071715	1.191140578	0.008303683
Rpl11	ENSMUSG00000059291	0.620254404	0.008304136
Rad18	ENSMUSG00000030254	1.434610416	0.008304136
Tti1	ENSMUSG00000027650	-0.80317612	0.008316248
Tbc1d15	ENSMUSG00000020130	0.618383416	0.008325909
Cdc42ep4	ENSMUSG00000041598	0.623742542	0.008344402
Dysf	ENSMUSG00000033788	0.570908635	0.008358044
Plcxd2	ENSMUSG00000087141	3.980480796	0.008358044
Plp2	ENSMUSG00000031146	0.623606102	0.008370043
Gpatch1	ENSMUSG00000063808	-0.809843174	0.008396537
Tnnt1	ENSMUSG00000064179	-0.836070698	0.008476686
Tcap	ENSMUSG00000007877	-0.84968708	0.008476686
Ska3	ENSMUSG00000021965	2.496386066	0.008508765
Pi16	ENSMUSG00000024011	0.635317467	0.008508765
Trip11	ENSMUSG00000021188	-0.732418914	0.008508765
Zfp853	ENSMUSG00000093910	4.499180701	0.008508765
Hacd1	ENSMUSG00000063275	0.709243143	0.008517863
Polrmt	ENSMUSG00000020329	-0.793966543	0.008538087
Fau	ENSMUSG00000038274	0.680705583	0.008542557

Ccdc186	ENSMUSG00000035173	-0.715698622	0.008557453
Gch1	ENSMUSG00000037580	0.764716676	0.008557478
Rasd1	ENSMUSG00000049892	1.082805818	0.008602241
Ankrd50	ENSMUSG00000044864	-0.726281866	0.008602241
Cfap36	ENSMUSG00000020462	0.650640427	0.008602241
Scpep1	ENSMUSG00000000278	0.631044442	0.008615239
Adgrl4	ENSMUSG00000039167	0.666800373	0.0086207
Rgs6	ENSMUSG00000021219	-0.70589965	0.008623499
Ezr	ENSMUSG00000052397	-0.619445651	0.008623499
Rps3a1	ENSMUSG00000028081	0.49793456	0.008635833
Mmaa	ENSMUSG00000037022	-0.79524714	0.008672171
Epb41l2	ENSMUSG00000019978	0.55061916	0.008675349
Cdh4	ENSMUSG00000000305	-2.074223957	0.008675349
Iqgap3	ENSMUSG00000028068	1.661132626	0.008688016
Reep3	ENSMUSG00000019873	0.556082207	0.008704622
Gpsm1	ENSMUSG00000026930	-0.578296858	0.008704622
Prlr	ENSMUSG00000005268	-1.931264226	0.008708811
Hmgcs2	ENSMUSG00000027875	-0.903001809	0.008718107
Ppp1r37	ENSMUSG00000051403	-0.543574055	0.008720453
Fkbp10	ENSMUSG00000001555	0.713755685	0.008750056
Rpl7a-ps5	ENSMUSG00000071052	0.765425687	0.008750599
Rpl35a-ps3	ENSMUSG00000067575	1.372546671	0.00876124
Bcar1	ENSMUSG00000031955	0.724624253	0.008804775
Col6a6	ENSMUSG00000043719	-1.129539993	0.008804775
Ash1l	ENSMUSG00000028053	-0.639521284	0.008812579
Map1lc3b	ENSMUSG00000031812	-0.477891511	0.008867501
Nme1	ENSMUSG00000037601	0.779072245	0.008867501
Upf2	ENSMUSG00000043241	-0.685065662	0.008867501
Fgf14	ENSMUSG00000025551	-1.293658407	0.00887678
Spsb4	ENSMUSG00000046997	1.001012892	0.008885544
Fastkd1	ENSMUSG00000027086	-0.944568563	0.008902399
Sgcg	ENSMUSG00000035296	-0.609292307	0.008906134
Hdgfl3	ENSMUSG00000025104	0.718825398	0.008952071
Eya2	ENSMUSG00000017897	1.130741574	0.008959559
Patj	ENSMUSG00000061859	-0.798833003	0.008959559
Dtx3	ENSMUSG00000040415	-0.695674165	0.008999033
Abcb10	ENSMUSG00000031974	-0.6607203	0.009005326
Tsr3	ENSMUSG00000015126	0.761616804	0.009031746
Phkb	ENSMUSG00000036879	-0.666272849	0.009072781
Gm12319	ENSMUSG00000087523	-1.507576194	0.009073115

Snai1	ENSMUSG00000042821	1.084657589	0.009080675
Dapk2	ENSMUSG00000032380	0.968323123	0.009081549
Aprt	ENSMUSG00000006589	0.72026038	0.009081549
Creb3l2	ENSMUSG00000038648	0.684064209	0.00908389
Fam149b	ENSMUSG00000039599	-0.575184073	0.009084984
Helt	ENSMUSG00000047171	-2.163242154	0.009087124
Fam171a2	ENSMUSG00000034685	0.846246818	0.009102899
Anapc13	ENSMUSG00000035048	-0.794861871	0.009134495
Pde1c	ENSMUSG00000004347	-0.74469305	0.009134495
Rps6	ENSMUSG00000028495	0.545376481	0.009134495
Cyba	ENSMUSG00000006519	0.641681927	0.009135128
Arntl	ENSMUSG00000055116	1.378036396	0.009150136
Traf1	ENSMUSG00000026875	1.347455571	0.009160035
Gsdma	ENSMUSG00000017204	4.299761926	0.009169978
Stmn2	ENSMUSG00000027500	0.949316413	0.009221174
2610507B11Rik	ENSMUSG00000010277	-0.56803898	0.009238047
Lpgat1	ENSMUSG00000026623	-0.577322441	0.009279194
Crybg3	ENSMUSG00000022723	-0.838134053	0.009279194
Creld2	ENSMUSG00000023272	0.765664121	0.009286871
Gdf3	ENSMUSG00000030117	3.418499935	0.009329424
Cfdp1	ENSMUSG00000031954	0.534049786	0.009329424
Herc1	ENSMUSG00000038664	-0.76643262	0.009345772
Arrb2	ENSMUSG00000060216	0.694587195	0.009357668
Hcn2	ENSMUSG00000020331	-0.660345421	0.009368003
Elmod3	ENSMUSG00000056698	-0.860717543	0.009377064
Tmem97	ENSMUSG00000037278	0.859635869	0.009377064
Rpl36a-ps2	ENSMUSG00000105388	0.831553752	0.009377064
Rpl6l	ENSMUSG00000091086	0.647396791	0.009377064
Gapdh	ENSMUSG00000057666	3.071091605	0.009393116
Cttn	ENSMUSG00000031078	0.607938459	0.009397508
Notch4	ENSMUSG00000015468	0.711168677	0.009410009
Zfp72	ENSMUSG00000069184	-1.255348835	0.009428662
Ssr4	ENSMUSG00000002014	0.603378951	0.00945193
Mnat1	ENSMUSG00000021103	0.623039554	0.009466068
Vars	ENSMUSG00000007029	0.654715186	0.009467672
Ptpn12	ENSMUSG00000028771	0.587670791	0.00947005
Apbb2	ENSMUSG00000029207	-0.60023892	0.009480219
Gm5561	ENSMUSG00000096474	1.314334073	0.009480219
Socs2	ENSMUSG00000020027	0.551631762	0.009493536
Rnf114	ENSMUSG00000006418	-0.53877324	0.009525274

Ptpn4	ENSMUSG00000026384	-0.804830115	0.009528061
Mast2	ENSMUSG00000003810	-0.5643655	0.009534818
Gm29237	ENSMUSG00000101872	-2.588333051	0.009534818
Tufm	ENSMUSG00000073838	-0.558892253	0.009536719
2700099C18Rik	ENSMUSG00000098090	2.368774376	0.00954619
Reep5	ENSMUSG00000005873	-0.569263077	0.009549779
Med11	ENSMUSG00000018923	0.75424349	0.009566049
Ccdc85b	ENSMUSG00000095098	0.645734035	0.009584662
Amacr	ENSMUSG00000022244	-0.775565774	0.009597114
Ppfibp2	ENSMUSG00000036528	-0.80613166	0.009634554
Ptk2	ENSMUSG00000022607	0.584125983	0.009671233
Zfp60	ENSMUSG00000037640	-0.922995974	0.009676799
Layn	ENSMUSG00000060594	0.861724958	0.009684041
Crip2	ENSMUSG00000006356	-0.509319055	0.009714029
Smarca1	ENSMUSG00000031099	1.349475669	0.009727626
Kmt2a	ENSMUSG00000002028	-0.789918291	0.009753938
Snx8	ENSMUSG00000029560	0.618682829	0.009766518
Dkk3	ENSMUSG00000030772	0.954257063	0.009774196
Lpar4	ENSMUSG00000049929	1.198728282	0.009824801
Tmem120a	ENSMUSG00000039886	0.579044845	0.009836268
Cyb5d1	ENSMUSG00000044795	-0.892590884	0.00990431
Gfm1	ENSMUSG00000027774	-0.601757234	0.009906885
Crk	ENSMUSG00000017776	0.499000296	0.009912078
Gm20686	ENSMUSG00000093697	-3.939289842	0.00991658
Dnajc27	ENSMUSG00000020657	-0.786899186	0.009922345
Sgo1	ENSMUSG00000023940	2.672451289	0.009940092
2900097C17Rik	ENSMUSG00000102869	-0.534828278	0.009940092
Clec12a	ENSMUSG00000053063	0.842297312	0.009940092
Prpf19	ENSMUSG00000024735	-0.550661197	0.009940092
Stx8	ENSMUSG00000020903	0.52425139	0.009969147
Hs1bp3	ENSMUSG00000020605	-0.82646845	0.009969147
Anxa11	ENSMUSG00000021866	-0.683688217	0.010028071
Patz1	ENSMUSG00000020453	-0.778268055	0.010036558
Gm37691	ENSMUSG00000104348	-1.548124721	0.010049973
Fam83d	ENSMUSG00000027654	2.053687468	0.010061126
Gm9531	ENSMUSG00000079225	0.867460756	0.010094923
Frmd8	ENSMUSG00000024816	0.725280483	0.010094923
Ube2d1	ENSMUSG00000019927	-0.710000879	0.01011148
Lyn	ENSMUSG00000042228	0.659275545	0.010120011
Itpr3	ENSMUSG00000042644	-0.873718759	0.010122545

Slc15a3	ENSMUSG00000024737	1.227931365	0.010122545
Tnfrsf1a	ENSMUSG00000030341	0.530148711	0.010167807
Slc41a3	ENSMUSG00000030089	-0.841083342	0.010252825
Ctxn3	ENSMUSG00000069372	3.183677987	0.01025811
Flnc	ENSMUSG00000068699	0.805276401	0.010376386
mt-Tq	ENSMUSG00000064343	-2.665554295	0.010376386
Shmt1	ENSMUSG00000020534	-0.961626012	0.010417883
Gm6863	ENSMUSG00000043483	0.542368134	0.010442952
Mas1	ENSMUSG00000068037	-5.48081932	0.010540626
Nasp	ENSMUSG00000028693	0.646501548	0.010545758
Twnk	ENSMUSG00000025209	-0.780025691	0.010554881
Gm8121	ENSMUSG00000051116	-0.674346356	0.010554881
Nmt1	ENSMUSG00000020936	0.590173246	0.010561467
Actr10	ENSMUSG00000021076	0.506025669	0.010571694
Eif4ebp2	ENSMUSG00000020091	-0.569173328	0.010585245
Psma6	ENSMUSG00000021024	0.453718657	0.010600171
Foxj2	ENSMUSG00000003154	-0.796344693	0.010630874
Gm17281	ENSMUSG00000097317	-0.957411804	0.010645179
Zfp882	ENSMUSG00000089857	-1.929183541	0.010659712
Parp14	ENSMUSG00000034422	-0.823658221	0.010707218
Gng8	ENSMUSG00000063594	1.6902114	0.010728898
Gm15867	ENSMUSG00000089812	2.29453087	0.010728989
Jup	ENSMUSG00000001552	-0.504654568	0.010770133
Nop9	ENSMUSG00000019297	0.621530744	0.010808174
Rab6b	ENSMUSG00000032549	-0.770090323	0.010813862
Gstz1	ENSMUSG00000021033	-0.598636697	0.01084322
Ppme1	ENSMUSG00000030718	0.590450913	0.010852693
Lonp1	ENSMUSG00000041168	0.51127531	0.010882648
Gprc5c	ENSMUSG00000051043	-1.194071977	0.010902978
Slc4a3	ENSMUSG00000006576	-0.701147472	0.010930687
Kat6a	ENSMUSG00000031540	-0.740374334	0.010930687
Klf4	ENSMUSG00000003032	0.618144203	0.010938591
Lck	ENSMUSG00000000409	1.925779723	0.010965329
2010204K13Rik	ENSMUSG00000063018	2.093498601	0.010988309
Stard3	ENSMUSG00000018167	-0.737176621	0.011007676
Tmprss6	ENSMUSG00000016942	3.182307242	0.011016627
Col5a3	ENSMUSG00000004098	1.072786357	0.011032852
Gm30970	ENSMUSG00000115637	-1.537532722	0.011043809
Aco2	ENSMUSG00000022477	0.714291863	0.011043809
Il1rn	ENSMUSG00000026981	1.774393424	0.011043809

Rps23	ENSMUSG00000049517	0.607864705	0.011044429
S100a13	ENSMUSG00000042312	0.7287771	0.011051487
Ggh	ENSMUSG00000073987	0.596497851	0.011070018
Ndufa4	ENSMUSG00000029632	-0.521492113	0.011077447
Dnaja4	ENSMUSG00000032285	0.626902102	0.0111002
Hemk1	ENSMUSG00000032579	-0.739317733	0.011104444
Rragb	ENSMUSG00000041658	-1.311674191	0.011132751
Hrc	ENSMUSG00000038239	-1.421097408	0.011165052
Rpl31-ps8	ENSMUSG00000067870	0.649223853	0.011177048
Snhg5	ENSMUSG00000097195	0.981175357	0.011177048
2810414N06Rik	ENSMUSG00000100594	-1.303053423	0.011200824
Phf2	ENSMUSG00000038025	-0.605343721	0.011212399
Pdlim4	ENSMUSG00000020388	-0.743564211	0.011212399
Gm36535	ENSMUSG00000105376	-2.002581668	0.01124555
Akr1b7	ENSMUSG00000052131	4.854947638	0.01124555
Cited4	ENSMUSG00000070803	-0.915637934	0.011253223
Bdnf	ENSMUSG00000048482	0.813513307	0.011257432
Dpy19l1	ENSMUSG00000043067	0.677940352	0.01125871
Zfp612	ENSMUSG00000044676	-1.346529533	0.011271777
Grhpr	ENSMUSG00000035637	0.525760067	0.011271777
Gm4535	ENSMUSG00000091083	-1.069334578	0.011311
Kiz	ENSMUSG00000074749	-0.619409365	0.011321045
Sirt7	ENSMUSG00000025138	0.738851478	0.011329355
Tedc1	ENSMUSG00000037466	1.93204317	0.011332337
Lrch2	ENSMUSG00000031290	1.113429749	0.011340407
Rgn	ENSMUSG00000023070	-3.601907001	0.011377783
Msx1	ENSMUSG00000048450	0.958450254	0.011377783
Mgat5b	ENSMUSG00000043857	2.849170197	0.01140042
Npl	ENSMUSG00000042684	1.150731047	0.011411164
Dok5	ENSMUSG00000027560	2.059625266	0.011411164
Slc30a4	ENSMUSG00000005802	0.690799458	0.011431654
Mdk	ENSMUSG00000027239	1.081966679	0.011434555
Bnc1	ENSMUSG00000025105	-1.7428373	0.011466622
Rps16	ENSMUSG00000037563	0.647374196	0.011466663
Inpp5d	ENSMUSG00000026288	0.695887706	0.011472127
Agtpbp1	ENSMUSG00000021557	-0.677820457	0.011564325
Bcl10	ENSMUSG00000028191	0.587200494	0.011583854
Fcgr1	ENSMUSG00000015947	0.829592547	0.011603815
Tmem9b	ENSMUSG00000031021	0.531068824	0.011636728
Gm12519	ENSMUSG00000085643	-1.782458867	0.011685549

Marf1	ENSMUSG00000060657	-0.593291904	0.011715283
Mto1	ENSMUSG00000032342	-0.709057292	0.011782776
Angpt4	ENSMUSG00000027460	2.299864425	0.011793741
Fkbp1a	ENSMUSG00000032966	0.534103143	0.011846852
Plet1os	ENSMUSG00000101304	-1.07512694	0.011864864
Clca3a2	ENSMUSG00000028262	-5.449180216	0.011903743
Pard6g	ENSMUSG00000056214	0.837234565	0.01200697
Drd2	ENSMUSG00000032259	-0.99989717	0.01200697
Tusc3	ENSMUSG00000039530	0.524736787	0.01200697
Vegfd	ENSMUSG00000031380	0.890331757	0.012009954
Kcnd3	ENSMUSG00000040896	-1.165239379	0.012012324
Nt5dc3	ENSMUSG00000054027	-0.626447113	0.01209899
Gm2830	ENSMUSG00000086567	1.272561757	0.012101074
Acp1	ENSMUSG00000044573	0.566124165	0.012122202
Calr3	ENSMUSG00000019732	-0.776211081	0.012122202
Neil3	ENSMUSG00000039396	2.627330438	0.012122202
Zkscan7	ENSMUSG00000063488	-1.589192604	0.012193674
Fam180a	ENSMUSG00000047420	1.484631294	0.012245036
Ext1	ENSMUSG00000061731	0.681617208	0.012370598
Pafah2	ENSMUSG00000037366	-0.80622513	0.012386112
Crabp2	ENSMUSG00000004885	5.465431513	0.012392815
Lonrf1	ENSMUSG00000039633	-0.866953487	0.012437562
Coa5	ENSMUSG00000026112	-0.509047155	0.012442016
Gm31812	ENSMUSG00000109599	-3.525425546	0.012442741
Gm6136	ENSMUSG00000084106	0.655878132	0.012465006
Tmx4	ENSMUSG00000034723	-0.541527455	0.012491903
AW549877	ENSMUSG00000041935	-0.609059169	0.012505723
Tslrn1	ENSMUSG00000085757	5.32565765	0.012571012
Nus1	ENSMUSG00000023068	0.509586631	0.012573524
D5Ertd579e	ENSMUSG00000029190	-0.56833944	0.012597543
Cdon	ENSMUSG00000038119	-0.950066069	0.012701463
AU020206	ENSMUSG00000097415	-0.924163151	0.012701463
Fzd1	ENSMUSG00000044674	0.824452665	0.012701654
Iscu	ENSMUSG00000025825	-0.683793633	0.01276278
Epm2aip1	ENSMUSG00000046785	-0.699960344	0.012817778
Egln2	ENSMUSG00000058709	0.5384327	0.012843871
Gtf2h1	ENSMUSG00000006599	0.613680154	0.012898992
mt-Nd6	ENSMUSG00000064368	-0.77197703	0.01295663
Rfc2	ENSMUSG00000023104	0.586194619	0.012963808
Prrc2a	ENSMUSG00000024393	-0.529857808	0.012991531

Ssb	ENSMUSG00000068882	0.498270702	0.013040856
Lclat1	ENSMUSG00000054469	-0.692110274	0.013040856
Perm1	ENSMUSG00000078486	-0.720772775	0.013040856
Poldip2	ENSMUSG00000001100	-0.475743305	0.013049986
Pdzd4	ENSMUSG00000002006	1.044226812	0.013049986
Mpdz	ENSMUSG00000028402	-0.55152323	0.013052078
Shld1	ENSMUSG00000044991	-0.855437367	0.013104694
Bnip3l	ENSMUSG00000022051	0.589919438	0.013143488
Nop56	ENSMUSG00000027405	0.600483537	0.013151695
Sqle	ENSMUSG00000022351	1.402944052	0.013177806
Rhod	ENSMUSG00000041845	0.79599584	0.013186175
Tns1	ENSMUSG00000055322	-0.681358486	0.013186175
Cybrd1	ENSMUSG00000027015	-1.449294935	0.013204478
Slc26a3	ENSMUSG00000001225	-1.400327891	0.013211133
Mavs	ENSMUSG00000037523	-0.525543094	0.013214717
Cd84	ENSMUSG00000038147	0.910190927	0.013246971
Dcun1d3	ENSMUSG00000048787	0.731630703	0.013274392
Abcg2	ENSMUSG00000029802	0.599957386	0.01328644
Pld3	ENSMUSG00000003363	0.570246769	0.013300273
Tmem179	ENSMUSG00000054013	-2.110492839	0.013321642
AC121151.1	ENSMUSG00000116757	1.020907512	0.013323432
Impdh2	ENSMUSG00000062867	0.534387302	0.013348264
P4htm	ENSMUSG00000006675	-0.744827741	0.013368888
Xirp1	ENSMUSG00000079243	0.763370136	0.013368888
Stat5b	ENSMUSG00000020919	-0.56692601	0.013466352
Cd40	ENSMUSG00000017652	0.956758307	0.013466352
Ccnd1	ENSMUSG00000070348	0.654621634	0.013485792
Ehd2	ENSMUSG00000074364	0.50642434	0.013502987
Ufsp2	ENSMUSG00000031634	0.672040193	0.013517345
Ap2m1	ENSMUSG00000022841	0.447510191	0.013517571
Abr	ENSMUSG00000017631	-0.664418679	0.01357182
Cd274	ENSMUSG00000016496	-1.129978491	0.013581736
Gm3788	ENSMUSG00000094392	0.988382833	0.013613992
Rassf10	ENSMUSG00000098132	-1.171134035	0.013630107
Gsto1	ENSMUSG00000025068	0.476367267	0.013779849
Selenom	ENSMUSG00000075702	0.669865295	0.013785431
Gm10736	ENSMUSG00000096842	-1.726579147	0.013785431
E230013L22Rik	ENSMUSG00000096957	1.043416944	0.013785674
Naa40	ENSMUSG00000024764	-0.764044753	0.013791287
Arhgap15	ENSMUSG00000049744	0.991496301	0.013791287

1110008L16Rik	ENSMUSG00000021023	-0.846875554	0.013796844
Ephx3	ENSMUSG00000037577	2.191191124	0.01379794
Gm19461	ENSMUSG00000101693	-1.174558576	0.01379794
Gng12	ENSMUSG00000036402	0.562711947	0.013802787
Gm7694	ENSMUSG00000102752	-1.004194422	0.013818029
Frs3	ENSMUSG00000023266	-1.098476516	0.013818029
Smim26	ENSMUSG00000074754	-0.619326246	0.013832465
Vamp2	ENSMUSG00000020894	-0.520954137	0.013864117
9030619P08Rik	ENSMUSG00000053168	-5.338897351	0.013867641
Tm6sf1	ENSMUSG00000038623	0.870835676	0.013876511
Adgrl2	ENSMUSG00000028184	-0.75702797	0.013878586
Kras	ENSMUSG00000030265	0.516596523	0.013883792
Cep83os	ENSMUSG00000097164	-0.969707793	0.013883792
Gm5905	ENSMUSG00000110275	1.318215167	0.013883792
Fbxl15	ENSMUSG00000025226	0.772974765	0.013916139
Gm33543	ENSMUSG00000110353	-1.592004081	0.013942298
Tmem82	ENSMUSG00000043085	-1.132210157	0.013965266
Dlg4	ENSMUSG00000020886	0.886120677	0.014044578
Gpr65	ENSMUSG00000021886	1.093398682	0.014044578
Nifk	ENSMUSG00000026377	0.591523647	0.014068635
Taf4	ENSMUSG00000039117	-0.764865577	0.01407453
Mrps21	ENSMUSG00000054312	-0.663096357	0.014085962
Ano4	ENSMUSG00000035189	-1.186561995	0.014148984
Six4	ENSMUSG00000034460	-1.537256963	0.01417449
Vps13d	ENSMUSG00000020220	-0.748983052	0.01417449
Rpl9-ps6	ENSMUSG00000062456	0.696996082	0.014190401
Tmem266	ENSMUSG00000032313	-1.195959284	0.014211673
Vps13c	ENSMUSG00000035284	-0.917417258	0.014244741
Dcaf13	ENSMUSG00000022300	0.576209713	0.014251567
Atp8b1	ENSMUSG00000039529	0.830643407	0.014277635
Psmb6	ENSMUSG00000018286	0.439619192	0.014296599
Ldb3	ENSMUSG00000021798	-0.568792464	0.014399689
Slc16a8	ENSMUSG00000032988	-2.157957347	0.014399689
Eif3i	ENSMUSG00000028798	0.467703319	0.014399689
Ppp2r2d	ENSMUSG00000041769	0.526156092	0.014399689
Mnt	ENSMUSG00000000282	-0.788556945	0.014400875
Des	ENSMUSG00000026208	0.722681772	0.014412296
Rps24	ENSMUSG00000025290	0.535793693	0.014430461
Rab11fip2	ENSMUSG00000040022	-1.075169923	0.014431713
Dusp4	ENSMUSG00000031530	1.777629123	0.01445407

Ypel3	ENSMUSG00000042675	-0.654458986	0.014459537
Trim16	ENSMUSG00000047821	0.9126025	0.014491864
Gjc2	ENSMUSG00000043448	1.195131223	0.014541383
Itm2c	ENSMUSG00000026223	0.510197044	0.014541383
Sp140	ENSMUSG00000070031	0.90722411	0.014546701
Eif2s1	ENSMUSG00000021116	0.51935272	0.014546701
Txndc9	ENSMUSG00000058407	0.462247533	0.014562885
Cog5	ENSMUSG00000035933	-0.677943078	0.014579233
Ehd4	ENSMUSG00000027293	0.609600582	0.014590295
Zbtb12	ENSMUSG00000049823	-1.042647597	0.014590295
Akr1b3	ENSMUSG00000001642	-0.54016347	0.0146273
Ank3	ENSMUSG00000069601	-0.695642659	0.014664474
Ift74	ENSMUSG00000028576	0.767949113	0.014679706
Olfml2b	ENSMUSG00000038463	0.692753171	0.014682968
Gm28723	ENSMUSG00000101028	2.45001027	0.014751723
Bik	ENSMUSG00000016758	-0.866141269	0.014751723
Ric8a	ENSMUSG00000025485	0.533945239	0.014755176
Gm44275	ENSMUSG00000107768	5.373598716	0.014786726
Gm9794	ENSMUSG00000107176	0.589111779	0.014809065
G2e3	ENSMUSG00000035293	1.020967984	0.014816326
Mapkapk3	ENSMUSG00000032577	-0.577767232	0.014853899
Erc1	ENSMUSG00000030172	-0.88001757	0.014879201
Cds2	ENSMUSG00000058793	-0.510118938	0.014882102
Dnajc12	ENSMUSG00000036764	-0.725821316	0.014911808
Tha1	ENSMUSG00000017713	-0.959901908	0.014926464
Il1rl2	ENSMUSG00000070942	1.058754011	0.014926464
Mfap3l	ENSMUSG00000031647	-1.015688276	0.014959787
Atp5a1	ENSMUSG00000025428	-0.489230624	0.014988632
2310016G11Rik	ENSMUSG00000070574	-1.8172025	0.014992325
Rab11fip3	ENSMUSG00000037098	-0.728328789	0.015047239
Klf12	ENSMUSG00000072294	-1.202355651	0.015112035
Sh3pxd2b	ENSMUSG00000040711	0.912645912	0.015130824
Tmem176b	ENSMUSG00000029810	0.547108323	0.015153793
Agl	ENSMUSG00000033400	-0.635656449	0.015180422
Asap3	ENSMUSG00000036995	-0.816558577	0.015201049
Maged2	ENSMUSG00000025268	0.570878889	0.015234495
Snx4	ENSMUSG00000022808	0.492949018	0.015234495
Gfra4	ENSMUSG00000027316	-1.114706825	0.015251233
Col28a1	ENSMUSG00000068794	2.105620449	0.015274029
Ybey	ENSMUSG00000033126	-1.117096697	0.015282099

Fam217b	ENSMUSG00000070476	-0.838143511	0.015302054
Mlip	ENSMUSG00000032355	-0.489957216	0.01533827
Asb16	ENSMUSG00000034768	-1.835778406	0.015344888
Rad54l2	ENSMUSG00000040661	-0.927120438	0.015352989
Mmrn1	ENSMUSG00000054641	0.839321471	0.015361881
Crebl2	ENSMUSG00000032652	-0.684041701	0.01548668
2310009A05Rik	ENSMUSG00000098332	-0.903529191	0.015492181
Ace2	ENSMUSG00000015405	1.580279892	0.015516001
Cox6a1	ENSMUSG00000041697	0.553064145	0.015521989
Mmp2	ENSMUSG00000031740	0.535077914	0.015615136
1810059H22Rik	ENSMUSG00000108207	-2.069123743	0.015615136
Nbeal2	ENSMUSG00000056724	-0.985621971	0.015616079
Col11a1	ENSMUSG00000027966	4.707872349	0.015668151
Gm15772	ENSMUSG00000062353	0.557906091	0.015669459
Gm9118	ENSMUSG00000112825	1.112313546	0.015696564
D430042O09Rik	ENSMUSG00000032743	-0.890037704	0.015707226
Myl12a	ENSMUSG00000024048	0.604737514	0.015715875
Sun1	ENSMUSG00000036817	-0.567679354	0.01573474
mt-Co2	ENSMUSG00000064354	-1.495290365	0.015785082
Phyhip	ENSMUSG00000003469	-0.806339135	0.015787882
Myo18b	ENSMUSG00000072720	-0.734302274	0.015796617
Gm31663	ENSMUSG00000108994	-1.11040021	0.01587821
Gtf2b	ENSMUSG00000028271	0.596839884	0.015909049
Gm7117	ENSMUSG00000063442	-1.469039714	0.015967836
Pgk1-rs7	ENSMUSG00000066632	1.399125384	0.015999645
Gm14403	ENSMUSG00000094786	-0.649392209	0.01606938
Col13a1	ENSMUSG00000058806	-1.384965582	0.016156032
Ube2i	ENSMUSG00000015120	0.475127948	0.016168276
Thap4	ENSMUSG00000026279	-0.588999208	0.016174838
Dcaf11	ENSMUSG00000022214	-0.553350525	0.016209288
Cystm1	ENSMUSG00000046727	0.550644219	0.016231255
Zfp710	ENSMUSG00000048897	-0.644292009	0.016252598
Tex264	ENSMUSG00000040813	0.523866653	0.01625948
Cox17	ENSMUSG00000046516	-1.779456331	0.016262625
Shox2	ENSMUSG00000027833	1.366471611	0.016262625
Sqstm1	ENSMUSG00000015837	0.53383403	0.01631448
Gm30459	ENSMUSG00000108468	-2.395979273	0.016378489
Cela1	ENSMUSG00000023031	1.624391827	0.016378489
Snhg18	ENSMUSG00000096956	0.899681071	0.016447101
Sh2d1b1	ENSMUSG00000102418	2.089914068	0.016511975

Gm14117	ENSMUSG00000042938	-0.633173791	0.016511975
Lin52	ENSMUSG00000085793	-0.92054589	0.016645284
Ptbp3	ENSMUSG00000028382	0.598098753	0.016675511
Slc27a3	ENSMUSG00000027932	1.611957483	0.016677552
Sema4d	ENSMUSG00000021451	-0.64453875	0.016677552
Id4	ENSMUSG00000021379	1.788386644	0.016725445
Tnfaip8l2	ENSMUSG00000013707	0.810042195	0.016742331
Tmod4	ENSMUSG00000005628	-1.788618075	0.016790174
Plod1	ENSMUSG00000019055	0.56384585	0.016860362
Gab2	ENSMUSG00000004508	0.925338393	0.016936517
Dbp	ENSMUSG00000059824	-0.840342614	0.017002878
Rpl18a	ENSMUSG00000045128	0.49867444	0.017046975
Zfp346	ENSMUSG00000021481	-0.67081009	0.017046975
Leprot	ENSMUSG00000035212	0.549803733	0.017046975
Htatip2	ENSMUSG00000039745	-0.712829226	0.017047963
Fgfr3	ENSMUSG00000054252	0.844713596	0.017075182
Psmd14	ENSMUSG00000026914	0.490441358	0.017077361
Lpl	ENSMUSG00000015568	-0.717118233	0.017084878
Sbk1	ENSMUSG00000042978	-0.833603708	0.017085752
Gpatch4	ENSMUSG00000028069	0.714356703	0.017116907
Snx6	ENSMUSG00000005656	0.479911092	0.017172171
Mex3c	ENSMUSG00000037253	0.612565618	0.017194359
E2f6	ENSMUSG00000057469	-0.589556644	0.017209204
Maoa	ENSMUSG00000025037	0.712475933	0.017213098
Pi4k2b	ENSMUSG00000029186	1.026097173	0.017228537
B4galt3	ENSMUSG00000052423	-0.962031151	0.017228537
Sorcs2	ENSMUSG00000029093	-1.216179505	0.017231846
Arap1	ENSMUSG00000032812	0.499827545	0.01723886
Psma7	ENSMUSG00000027566	0.438545605	0.017276984
Nanos1	ENSMUSG00000072437	1.85641845	0.017426212
Casq2	ENSMUSG00000027861	-0.559251046	0.017444681
Cd3eap	ENSMUSG00000047649	0.608137346	0.01746734
Hspa5	ENSMUSG00000026864	0.552712588	0.017480701
Etv3	ENSMUSG00000003382	-0.592624098	0.017532585
Bmp2k	ENSMUSG00000034663	0.723683643	0.017585856
Zfp609	ENSMUSG00000040524	-0.849542992	0.017608322
Gm5532	ENSMUSG00000073535	-0.854756466	0.017638334
Ptpn1	ENSMUSG00000027540	0.700240997	0.017639496
Rpl32	ENSMUSG00000057841	0.526271667	0.017647874
Il11ra1	ENSMUSG00000073889	0.476998213	0.017666213

Blk	ENSMUSG00000014453	1.778247188	0.017670745
Prokr1	ENSMUSG00000049409	-4.738149633	0.017678439
Usp24	ENSMUSG00000028514	-0.572663738	0.017697764
Ivns1abp	ENSMUSG00000023150	-0.595394894	0.017697764
Il3ra	ENSMUSG00000068758	0.830367269	0.017856799
Pycr2	ENSMUSG00000026520	0.640630992	0.017856835
Cd109	ENSMUSG00000046186	1.539221134	0.017859413
Cecr2	ENSMUSG00000071226	-1.358510281	0.017862311
Rpf2	ENSMUSG00000038510	0.585739347	0.017924914
1700108F19Rik	ENSMUSG00000101009	-5.268918805	0.017931611
Cxcl10	ENSMUSG00000034855	1.721490783	0.017931611
Samd14	ENSMUSG00000047181	0.801800693	0.017939055
Dcaf1	ENSMUSG00000040325	-0.678965294	0.017970999
Ccnjl	ENSMUSG00000044707	-2.688047146	0.01800627
Fam135a	ENSMUSG00000026153	-0.648957339	0.018068699
Rbms1	ENSMUSG00000026970	0.528116774	0.018102429
Sv2a	ENSMUSG00000038486	-1.54138189	0.018127016
B330016D10Rik	ENSMUSG00000048406	-0.900875483	0.018170973
Chchd7	ENSMUSG00000042198	-0.601708535	0.018204187
Gpd1l	ENSMUSG00000050627	-0.539439466	0.018205128
Sumf1	ENSMUSG00000030101	0.599528364	0.018209434
Clmp	ENSMUSG00000032024	0.94002503	0.018216376
Kcnj15	ENSMUSG00000062609	1.867648635	0.018223794
Rab23	ENSMUSG00000004768	0.774615572	0.018266888
Pdss2	ENSMUSG00000038240	-0.702725805	0.018298173
Gm34276	ENSMUSG00000113977	-2.088649031	0.018329907
Gnmt	ENSMUSG00000002769	-2.013718754	0.018329907
Tnfrsf19	ENSMUSG00000060548	-1.435303488	0.018330416
Cox19	ENSMUSG00000045438	0.663442009	0.018334684
G0s2	ENSMUSG00000009633	-0.832982269	0.018348758
Cox5a	ENSMUSG00000000088	-0.529861212	0.018401927
Psme1	ENSMUSG00000022216	-0.580476419	0.018413753
Hist1h4h	ENSMUSG00000060981	-0.690850545	0.018551829
1810030O07Rik	ENSMUSG00000044148	-0.578918459	0.018556334
Ogt	ENSMUSG00000034160	-0.654257973	0.018556334
Rspo1	ENSMUSG00000028871	-1.008546221	0.018584601
Tnfrsf18	ENSMUSG00000041954	2.904723999	0.018598648
Casp4	ENSMUSG00000033538	0.775824606	0.018631779
Rpl35a	ENSMUSG00000060636	0.646441442	0.018631779
Ctnna3	ENSMUSG00000060843	-0.690375005	0.018632636

Fus	ENSMUSG00000030795	-0.479880956	0.018640932
Stim2	ENSMUSG00000039156	0.621565688	0.018640932
Uqcrfs1	ENSMUSG00000038462	-0.527589055	0.018716002
Rtf2	ENSMUSG00000027502	0.495506337	0.018719319
Mycbpap	ENSMUSG00000039110	-4.176760037	0.018754133
Epsti1	ENSMUSG00000022014	0.780341954	0.018792856
Cdca5	ENSMUSG00000024791	2.030801842	0.018825942
Pla2g4e	ENSMUSG00000050211	-1.523206165	0.018837268
Mplkip	ENSMUSG00000012429	0.670076537	0.01888976
Plxna4	ENSMUSG00000029765	-0.825104321	0.018896993
Dync1li1	ENSMUSG00000032435	0.57382202	0.018896993
Cd68	ENSMUSG00000018774	0.597933955	0.018896993
Epb41	ENSMUSG00000028906	-0.643193596	0.018924463
Gm43197	ENSMUSG00000107215	-4.149179003	0.018951671
Cenpb	ENSMUSG00000068267	0.500353673	0.018960807
Pdzrn3	ENSMUSG00000035357	0.813123096	0.018967071
Stt3a	ENSMUSG00000032116	0.523310045	0.018967071
Man2a2	ENSMUSG00000038886	-0.654844196	0.018982493
Efcab11	ENSMUSG00000021176	3.380684212	0.019072754
Nmt2	ENSMUSG00000026643	0.658957832	0.019162992
Ercc2	ENSMUSG00000030400	-1.139930506	0.019162992
Btg3	ENSMUSG00000022863	0.751622528	0.019211724
Tmem150a	ENSMUSG00000055912	-0.717572683	0.0192604
Gm45012	ENSMUSG00000109052	-1.53959101	0.019270987
Mapk4	ENSMUSG00000024558	1.209881267	0.019270987
Cdk5rap3	ENSMUSG00000018669	0.537750378	0.019270987
Mars	ENSMUSG00000040354	0.655125805	0.019271312
Exosc2	ENSMUSG00000039356	0.74312354	0.019274988
Fbf1	ENSMUSG00000020776	-0.752702457	0.019345626
Nt5c1a	ENSMUSG00000054958	-1.648317781	0.019422057
Cbfb	ENSMUSG00000031885	0.493116901	0.0194352
Ubtd1	ENSMUSG00000025171	0.630005473	0.0194352
Cpxm1	ENSMUSG00000027408	0.615223209	0.019461382
Atg2a	ENSMUSG00000024773	-0.67820765	0.019507006
2-Mar	ENSMUSG00000073481	0.502060799	0.019507006
Pbdc1	ENSMUSG00000031226	0.715663882	0.0195319
3110053B16Rik	ENSMUSG00000085642	2.21940867	0.019562021
Med25	ENSMUSG00000002968	-0.508147468	0.019563878
Gm14267	ENSMUSG00000085682	-1.02407734	0.019598065
Serpine2	ENSMUSG00000026249	0.525290517	0.019632817

1810055G02Rik	ENSMUSG00000035372	0.773179822	0.019646084
Mtfr1	ENSMUSG00000027601	-0.510986962	0.019695341
Ces2e	ENSMUSG00000031886	1.33516185	0.019736173
Zrsr2	ENSMUSG00000031370	0.682347107	0.019768074
Galnt16	ENSMUSG00000021130	0.887534373	0.019771189
Abca2	ENSMUSG00000026944	-0.691502795	0.019771189
2310001H17Rik	ENSMUSG00000097354	-1.251036894	0.019771189
Acta1	ENSMUSG00000031972	1.393053999	0.019771189
Hdac1	ENSMUSG00000028800	0.565585981	0.019844605
P4ha2	ENSMUSG00000018906	0.573216656	0.019844605
Ccdc66	ENSMUSG00000046753	-0.76781021	0.019844605
Clcn3	ENSMUSG00000004319	-0.649908614	0.019877396
B3gnt7	ENSMUSG00000079445	-2.335351006	0.019877396
Ezh1	ENSMUSG00000006920	-0.548218277	0.019900156
Prelid2	ENSMUSG00000056671	0.856340475	0.019913169
Masp1	ENSMUSG00000022887	0.840940292	0.019952719
Zfp775	ENSMUSG00000007216	-0.653008984	0.019980255
Fstl3	ENSMUSG00000020325	1.295598584	0.019990659
Nlk	ENSMUSG00000017376	-0.699853192	0.020037518
Myrip	ENSMUSG00000041794	-0.794999156	0.020037518
Aig1	ENSMUSG00000019806	-0.646656699	0.020037518
Acvr1b	ENSMUSG00000000532	-0.725479534	0.020040142
Cand1	ENSMUSG00000020114	0.533390446	0.020064572
Cdc42ep5	ENSMUSG00000063838	0.830839983	0.020064572
Cript	ENSMUSG00000024146	0.489251302	0.020065565
Alyref	ENSMUSG00000025134	0.589246718	0.020127567
Spock2	ENSMUSG00000058297	-0.649487833	0.02017356
4932438A13Rik	ENSMUSG00000037270	-0.6423629	0.02017356
Smad1	ENSMUSG00000031681	0.642956144	0.02017356
Fig4	ENSMUSG00000038417	-0.560059697	0.020200013
Tmc7	ENSMUSG00000042246	-1.147785466	0.020200013
Kctd1	ENSMUSG00000036225	-0.744570196	0.020200013
Fosl2	ENSMUSG00000029135	0.775251065	0.020223366
Atp2a1	ENSMUSG00000030730	-5.32407264	0.020240855
Kif16b	ENSMUSG00000038844	-0.624737308	0.020240855
Tmem165	ENSMUSG00000029234	0.551258104	0.020267743
Polr1c	ENSMUSG00000067148	0.540417503	0.020343549
AC130815.1	ENSMUSG00000116617	1.725551424	0.020409886
Ankrd34a	ENSMUSG00000049097	-1.846168323	0.020422593
Pigv	ENSMUSG00000043257	-0.768918345	0.020497098

Clip2	ENSMUSG00000063146	0.649001249	0.020511987
Bcl11b	ENSMUSG00000048251	-2.114210781	0.020554025
Stx17	ENSMUSG00000061455	-0.559191581	0.020626785
Ddx3x	ENSMUSG00000000787	0.478179404	0.020626785
Srrm2	ENSMUSG00000039218	-0.595769612	0.020651183
Extl3	ENSMUSG00000021978	-0.614301689	0.020662215
Rin3	ENSMUSG00000044456	0.710666869	0.020806324
Rbbp4	ENSMUSG00000057236	0.455074062	0.020806324
Nudcd1	ENSMUSG00000038736	0.703984535	0.02086578
Irf2bp2	ENSMUSG00000051495	0.541430149	0.02086578
Sh3gl2	ENSMUSG00000028488	-2.549497767	0.02087344
Smu1	ENSMUSG00000028409	0.482065388	0.020933599
Ptpn13	ENSMUSG00000034573	-1.060121858	0.020948301
Prr5	ENSMUSG00000036106	0.859234488	0.02095039
Cpeb2	ENSMUSG00000039782	-0.684771714	0.020952645
Serpina3n	ENSMUSG00000021091	1.632168083	0.020959048
Tldc1	ENSMUSG00000034105	-0.664025504	0.020959048
Eif1ad	ENSMUSG00000024841	0.51950368	0.020960367
Cox4i2	ENSMUSG00000009876	0.892870687	0.020960367
Zmiz2	ENSMUSG00000041164	-0.59652353	0.020960367
Gm6419	ENSMUSG00000072407	-1.091893783	0.020970905
Ndufaf8	ENSMUSG00000078572	-0.63919817	0.021008626
Atad2	ENSMUSG00000022360	0.920775727	0.021066506
Pxn	ENSMUSG00000029528	0.609226587	0.021066506
Dapk1	ENSMUSG00000021559	-0.707263289	0.021134707
Gm16316	ENSMUSG00000087129	-1.951004468	0.021134707
Slc33a1	ENSMUSG00000027822	0.640678611	0.021148429
Atxn7	ENSMUSG00000021738	-0.711225414	0.021192976
Mrpl24	ENSMUSG00000019710	0.49459036	0.021192976
4921531C22Rik	ENSMUSG00000085704	-0.927045645	0.021196869
Camk2n2	ENSMUSG00000051146	1.279881399	0.021355963
Blvra	ENSMUSG00000001999	0.533429663	0.021379345
Klhl4	ENSMUSG00000025597	0.63738967	0.021401285
Ptpn18	ENSMUSG00000026126	0.985721117	0.02141458
Vamp3	ENSMUSG00000028955	0.489688389	0.021434657
Cfap43	ENSMUSG00000044948	0.807881024	0.021434657
Fndc3b	ENSMUSG00000039286	0.525443574	0.021450776
Acss3	ENSMUSG00000035948	-2.100108737	0.021462512
Mrpl28	ENSMUSG00000024181	-0.615345038	0.021493031
Tk1	ENSMUSG00000025574	1.160081308	0.021493031

Thyn1	ENSMUSG00000035443	0.546891455	0.021493031
Foxp2	ENSMUSG00000029563	0.909499701	0.021493031
Rnpepl1	ENSMUSG00000026269	-0.543785238	0.021493031
Gm12346	ENSMUSG00000083899	1.233592889	0.021496796
Rell1	ENSMUSG00000047881	0.666577834	0.021512994
Slc7a6	ENSMUSG00000031904	0.805424979	0.021528116
Smad3	ENSMUSG00000032402	-0.78474822	0.021534807
Setbp1	ENSMUSG00000024548	-0.879992011	0.021624723
Cnih1	ENSMUSG00000015759	0.453717633	0.021671149
Chrm3	ENSMUSG00000046159	-1.444725671	0.02167298
Gm12070	ENSMUSG00000069939	0.797548759	0.02167298
Coro2a	ENSMUSG00000028337	1.675723816	0.02167298
Hdc	ENSMUSG00000027360	1.04171626	0.021688965
Plxnb3	ENSMUSG00000031385	-1.496805087	0.021688965
0610040B10Rik	ENSMUSG00000089889	-0.873942291	0.021744411
Fzd8	ENSMUSG00000036904	-1.144347481	0.021750493
Asb12	ENSMUSG00000031204	-0.724217441	0.02175307
Cpm	ENSMUSG00000020183	1.141481686	0.021781045
Plec	ENSMUSG00000022565	-0.517684725	0.021869233
Tsc2	ENSMUSG00000002496	-0.650189031	0.021899427
Gm6311	ENSMUSG00000061833	0.733749037	0.021935378
Ager	ENSMUSG00000015452	5.163726393	0.021935378
Sox6os	ENSMUSG00000030664	-1.764977539	0.022047536
Scfd1	ENSMUSG00000020952	0.529978203	0.022070621
Lrtm1	ENSMUSG00000045776	-1.435613356	0.022101698
Ptprcap	ENSMUSG00000045826	2.309944197	0.02211563
Tmem218	ENSMUSG00000032121	-0.662046476	0.02218576
Ptges3-ps	ENSMUSG00000040078	0.580424656	0.022200364
Nup214	ENSMUSG00000001855	-0.784398406	0.022329472
Itga9	ENSMUSG00000039115	0.517390777	0.022428855
Psmb5	ENSMUSG00000022193	0.434241431	0.022445953
Pcdh15	ENSMUSG00000052613	-2.580924925	0.022458254
Degs1	ENSMUSG00000038633	0.437408656	0.022473481
Eif2b5	ENSMUSG00000003235	0.509697543	0.022503441
Sned1	ENSMUSG00000047793	-1.487558145	0.022519787
Slc45a4	ENSMUSG00000079020	-0.654018977	0.022638122
Zfp14	ENSMUSG00000053985	-1.183607697	0.022642319
Cd302	ENSMUSG00000060703	0.682958366	0.022668985
Inpp5k	ENSMUSG00000006127	-0.576317702	0.022668985
Gata2	ENSMUSG00000015053	-0.67111518	0.022668985

Mrpl44	ENSMUSG00000026248	-0.537833598	0.022668985
Begain	ENSMUSG00000040867	2.024307355	0.02268756
Asf1b	ENSMUSG00000005470	1.141952799	0.022696361
Serac1	ENSMUSG00000015659	-1.18878311	0.022731062
Asb13	ENSMUSG00000033781	-0.671343393	0.022731062
Myl1	ENSMUSG00000061816	-0.662533489	0.02276203
Bst2	ENSMUSG00000046718	0.793704869	0.02276203
Coq7	ENSMUSG00000030652	-0.563146293	0.022771738
Ccng1	ENSMUSG00000020326	-0.57331174	0.022771738
Smim3	ENSMUSG00000038059	-0.676088467	0.022801584
Mllt6	ENSMUSG00000038437	-0.577439831	0.02286545
Dram1	ENSMUSG00000020057	0.80308594	0.02286545
Phldb1	ENSMUSG00000048537	-0.499635004	0.022934536
Eif2b3	ENSMUSG00000028683	0.625992784	0.022979654
Tmem173	ENSMUSG00000024349	0.762973406	0.022984602
Sox17	ENSMUSG00000025902	0.7831107	0.023045922
Mga	ENSMUSG00000033943	-0.663495786	0.023090076
Prdx3	ENSMUSG00000024997	-0.489262581	0.023110418
Cyb5d2	ENSMUSG00000057778	-0.642192372	0.023141043
Tmcc2	ENSMUSG00000042066	-0.593084659	0.023207062
Emc1	ENSMUSG00000078517	-0.62377626	0.02321933
Trpc6	ENSMUSG00000031997	4.557534535	0.02321933
Gm11942	ENSMUSG00000094344	1.156881151	0.023224221
Them6	ENSMUSG00000056665	-0.688308172	0.023274496
Slc35b2	ENSMUSG00000037089	0.459835316	0.023276866
CAAA01118383.1	ENSMUSG00000063897	0.585614101	0.023281259
Vapa	ENSMUSG00000024091	0.426024638	0.023301311
Cdca7l	ENSMUSG00000021175	1.864249105	0.02330951
Msh2	ENSMUSG00000024151	-0.605951098	0.023376431
Foxc1	ENSMUSG00000050295	-0.577144786	0.023400665
Ndufa5	ENSMUSG00000023089	-0.565797022	0.023469053
Sntb1	ENSMUSG00000060429	1.0739479	0.023531712
Tspan17	ENSMUSG00000025875	0.733314192	0.023545569
Eef1g	ENSMUSG00000071644	0.411526779	0.023549057
Ttc30a2	ENSMUSG00000075272	-2.256235932	0.023561964
Arap2	ENSMUSG00000037999	-0.870542566	0.023571042
Cyp1b1	ENSMUSG00000024087	0.681880356	0.023613719
Polr1d	ENSMUSG00000029642	0.451453968	0.023613719
Gm20442	ENSMUSG00000092600	5.095533249	0.023614853
Pla1a	ENSMUSG00000002847	0.576509422	0.023658737

Lrig1	ENSMUSG00000030029	-0.573110065	0.023697093
Dmxl1	ENSMUSG00000037416	-0.586778004	0.023697641
Bace1	ENSMUSG00000032086	-0.673973904	0.02374415
Gsta3	ENSMUSG00000025934	-0.751819364	0.023745553
Mrps18b	ENSMUSG00000024436	0.634377832	0.023848869
Pgls	ENSMUSG00000031807	0.499469969	0.023848869
Psd3	ENSMUSG00000030465	-0.57514439	0.023862256
Uqcc1	ENSMUSG00000005882	-0.573062	0.023914822
Mcm3ap	ENSMUSG00000001150	-0.705854242	0.023914822
Gm13835	ENSMUSG00000086922	0.465436521	0.023934868
Myh7b	ENSMUSG00000074652	-0.87275833	0.023934868
Fblim1	ENSMUSG00000006219	-0.503214183	0.023934868
Entpd1	ENSMUSG00000048120	0.519174616	0.023998343
Gm4654	ENSMUSG00000098111	1.336819261	0.023998343
C1qa	ENSMUSG00000036887	0.620128236	0.023998343
2810430I11Rik	ENSMUSG00000085766	4.822193467	0.024053141
Lrpprc	ENSMUSG00000024120	-0.563202485	0.024099456
Klhl38	ENSMUSG00000022357	-1.044436449	0.024099456
Sec14l5	ENSMUSG00000091712	-2.45203557	0.024123672
Cox7b	ENSMUSG00000031231	-0.541289252	0.024124542
Pvt1	ENSMUSG00000097039	1.377871138	0.02416364
Cxcl9	ENSMUSG00000029417	1.131701342	0.024170895
Isca1	ENSMUSG00000044792	-0.545359814	0.024181662
Khdrbs3	ENSMUSG00000022332	-0.611336452	0.02435106
Fam84a	ENSMUSG00000020607	-1.625628256	0.02435106
Milr1	ENSMUSG00000040528	1.055203174	0.024370974
Gdpd5	ENSMUSG00000035314	-0.695603443	0.024486976
Rps9	ENSMUSG00000006333	0.523441605	0.024487331
Tfpi2	ENSMUSG00000029664	1.196377454	0.024516324
Rab39b	ENSMUSG00000031202	3.02758283	0.024568916
Fap	ENSMUSG00000000392	0.83753444	0.024634716
Fbxw7	ENSMUSG00000028086	-0.713966675	0.024635373
Pnck	ENSMUSG00000002012	-1.078321424	0.024666958
Retsat	ENSMUSG00000056666	-0.766900672	0.024666958
Nfe2l2	ENSMUSG00000015839	0.499005016	0.024666958
Pemt	ENSMUSG00000000301	1.192977518	0.024673577
Gnptab	ENSMUSG00000035311	-0.552330388	0.024677394
Prpf40a	ENSMUSG00000061136	0.529895228	0.0247039
2310075C17Rik	ENSMUSG00000089718	-4.641294672	0.024768297
Cnn1	ENSMUSG00000001349	1.27715207	0.024835593

Pdia6	ENSMUSG00000020571	0.516858691	0.024911971
Map2	ENSMUSG00000015222	1.138086769	0.024923536
Gm10705	ENSMUSG00000074506	-1.391698064	0.025001523
AC151284.1	ENSMUSG00000117192	-2.127469696	0.025001523
Rps6ka5	ENSMUSG00000021180	-0.813223867	0.025008638
Acad8	ENSMUSG00000031969	-0.538948462	0.025008638
Gm31166	ENSMUSG00000109695	-1.252099381	0.025012996
Rnd2	ENSMUSG00000001313	0.691745572	0.025029073
Sac3d1	ENSMUSG00000024790	0.566620429	0.025049467
Rreb1	ENSMUSG00000039087	-0.788400619	0.02514081
Ralgps2	ENSMUSG00000026594	-0.681159897	0.02514081
Il18r1	ENSMUSG00000026070	-1.680958766	0.025170099
H13	ENSMUSG00000019188	0.508755377	0.025217365
Card9	ENSMUSG00000026928	1.34707086	0.025242274
Fam207a	ENSMUSG00000032977	0.692235221	0.025458261
Sec13	ENSMUSG00000030298	0.442635204	0.025568713
Uba7	ENSMUSG00000032596	-0.611623184	0.025605551
B3galnt2	ENSMUSG00000039242	-0.671213662	0.025605551
Casd1	ENSMUSG00000015189	-0.703065774	0.025620699
Zfp422-ps	ENSMUSG00000091515	-1.77883091	0.025641293
Bphl	ENSMUSG00000038286	-0.709099079	0.025669909
Ube2e2	ENSMUSG00000058317	0.662238319	0.025669909
Btla	ENSMUSG00000052013	1.980887038	0.025868796
Atp6ap1l	ENSMUSG00000078958	-2.016831825	0.025875423
Nfkbil1	ENSMUSG00000042419	0.792916284	0.025875423
Ttc12	ENSMUSG00000040219	-0.689760977	0.025923792
Gm17473	ENSMUSG00000097805	-2.49154446	0.025928109
Pdlim5	ENSMUSG00000028273	0.485463098	0.025940827
Pparg	ENSMUSG00000000440	0.764619937	0.025944568
Rnf103	ENSMUSG00000052656	0.522656847	0.025944568
Tmem74b	ENSMUSG00000044364	1.035302849	0.026130327
Shc2	ENSMUSG00000020312	0.935610064	0.026139359
Abcc3	ENSMUSG00000020865	1.2301754	0.026139359
AC154478.1	ENSMUSG00000116725	-1.256177047	0.026140232
Fbxo17	ENSMUSG00000030598	1.220079072	0.026144227
Klk1b26	ENSMUSG00000053719	-4.086380191	0.026144227
Lat2	ENSMUSG00000040751	1.480952968	0.026148099
Zim1	ENSMUSG00000002266	-4.030638567	0.026148099
Pdp1	ENSMUSG00000049225	-0.537338861	0.026150298
Ttyh2	ENSMUSG00000034714	1.15190505	0.026240733

Cyp2d22	ENSMUSG00000061740	-0.642341657	0.026254356
Kif21a	ENSMUSG00000022629	-0.781316348	0.026360069
Ints11	ENSMUSG00000029034	0.644946461	0.026360069
BC067074	ENSMUSG00000021763	-2.776690669	0.026367813
Araf	ENSMUSG00000001127	-0.435619714	0.026438846
Asxl2	ENSMUSG00000037486	-0.695384264	0.026453541
Rpl18	ENSMUSG00000059070	0.501767184	0.026506845
2210011C24Rik	ENSMUSG00000074217	-1.073224065	0.026627226
Psmb8	ENSMUSG00000024338	0.701413907	0.026627226
mt-Co1	ENSMUSG00000064351	-0.614609263	0.026687476
Kctd6	ENSMUSG00000021752	0.671118702	0.026754162
Hrasls	ENSMUSG00000022525	-0.63615208	0.026792052
Gbp4	ENSMUSG00000079363	-0.790058357	0.026913902
Rgs19	ENSMUSG00000002458	0.694874553	0.026924422
Spsb3	ENSMUSG00000024160	0.849319178	0.026942751
Slc35d1	ENSMUSG00000028521	-0.878980606	0.027042007
Mad1l1	ENSMUSG00000029554	0.767346321	0.027042007
Mrpl30	ENSMUSG00000026087	-0.409908918	0.027047702
C530005A16Rik	ENSMUSG00000085408	-1.840079187	0.027053019
Zfp948	ENSMUSG00000067931	0.904432915	0.027077252
Spen	ENSMUSG00000040761	-0.914587082	0.027077252
Nsl1	ENSMUSG00000062510	1.726481021	0.027077252
Gm6658	ENSMUSG00000074171	-1.510504476	0.027152054
Gm36372	ENSMUSG00000113975	-0.789288753	0.027186614
Sacs	ENSMUSG00000048279	-0.859573959	0.027214856
Tcp1	ENSMUSG00000068039	0.502044719	0.0272452
Igsf9b	ENSMUSG00000034275	-1.471335671	0.027281734
Ndufa4l2	ENSMUSG00000040280	0.700357192	0.027286598
Atp6v1b2	ENSMUSG00000006273	0.503949681	0.027286598
Amz1	ENSMUSG00000050022	1.594060099	0.027374079
Cetn3	ENSMUSG00000021537	0.478834401	0.027374079
Smad9	ENSMUSG00000027796	-1.326835401	0.027374079
Gm12295	ENSMUSG00000085162	0.901022571	0.027374079
Mindy3	ENSMUSG00000026767	0.523235574	0.027374079
Ccnt2	ENSMUSG00000026349	-0.657949607	0.027375778
Tfdp2	ENSMUSG00000032411	-0.544745459	0.027437113
Gm10698	ENSMUSG00000079884	0.813250411	0.027437113
Agfg1	ENSMUSG00000026159	0.4838918	0.027441824
Isg20	ENSMUSG00000039236	0.738849946	0.027565401
Gm39822	ENSMUSG00000110618	2.106688582	0.027601524

N4bp3	ENSMUSG00000001053	0.755988634	0.027606286
Dnaja1	ENSMUSG00000028410	0.657522041	0.027657626
Rps18-ps3	ENSMUSG00000057657	-2.058704393	0.027657626
Rnf144a	ENSMUSG00000020642	0.707384833	0.027689717
Rpe	ENSMUSG00000026005	0.574037411	0.027710235
Zfp963	ENSMUSG00000092260	-1.622059851	0.027713706
Zfp446	ENSMUSG00000033961	-1.21927499	0.027713706
Cyc1	ENSMUSG00000022551	-0.420320232	0.027726869
Ndufa8	ENSMUSG00000026895	-0.43340897	0.027746632
Inhba	ENSMUSG00000041324	0.939407878	0.027803243
Otulinl	ENSMUSG00000056069	0.806561626	0.027803243
Rpl22	ENSMUSG00000028936	0.565202335	0.027817562
Bmper	ENSMUSG00000031963	-0.717217109	0.027851935
Camk2n1	ENSMUSG00000046447	0.519998829	0.027851935
Mir133a-1hg	ENSMUSG00000095438	-0.952337881	0.027851935
Nr1h3	ENSMUSG00000002108	-0.523375669	0.027851935
Ptpmt1	ENSMUSG00000063235	0.485966399	0.027854714
Sema5b	ENSMUSG00000052133	-2.000055606	0.027869444
Fam81a	ENSMUSG00000032224	-0.737254253	0.027869444
Arl4a	ENSMUSG00000047446	0.478997659	0.028024718
Hes6	ENSMUSG00000067071	-0.668547019	0.028064913
Cdc16	ENSMUSG00000038416	0.459471531	0.028073337
Tnrc18	ENSMUSG00000039477	-0.736965244	0.028079657
Slc39a1	ENSMUSG00000052310	0.55040694	0.028097747
Llgl2	ENSMUSG00000020782	-0.737075628	0.028133801
Rnf138	ENSMUSG00000024317	0.641831244	0.028334569
Hmcn2	ENSMUSG00000055632	-1.134482699	0.028348629
Csad	ENSMUSG00000023044	0.656110194	0.028442396
Rps15	ENSMUSG00000063457	0.467043421	0.028521067
Exo1	ENSMUSG00000039748	2.826889387	0.028521067
F2rl3	ENSMUSG00000050147	2.050912684	0.028654935
Foxd2os	ENSMUSG00000085399	1.641810068	0.028708913
Gpx1	ENSMUSG00000063856	0.57242635	0.028718169
Ing5	ENSMUSG00000026283	-0.671145037	0.028743614
Plek	ENSMUSG00000020120	0.769406301	0.028817363
Atp5c1	ENSMUSG00000025781	-0.431581913	0.028849752
Top1mt	ENSMUSG00000000934	-0.909902952	0.028854775
Insr	ENSMUSG00000005534	-0.556032758	0.028854775
Tdg	ENSMUSG00000034674	0.705037791	0.028861163
Cfl2	ENSMUSG00000062929	0.404250168	0.028965398

Dscc1	ENSMUSG00000022422	2.265955885	0.029007413
Zmat1	ENSMUSG00000052676	-0.815500575	0.029048855
Gm2574	ENSMUSG00000078162	1.712110911	0.029049617
Aqp11	ENSMUSG00000042797	-1.541086429	0.029115532
Acvrl1	ENSMUSG00000000530	0.504117469	0.029122897
Enkur	ENSMUSG00000026679	1.833077778	0.029126141
Usp35	ENSMUSG00000035713	1.545110345	0.029173994
Mrvi1	ENSMUSG00000005611	-1.064643254	0.029178329
Dse	ENSMUSG00000039497	0.58837597	0.029181383
Kif26b	ENSMUSG00000026494	1.284262612	0.029181383
Acsl5	ENSMUSG00000024981	0.535780439	0.029181383
Hspa13	ENSMUSG00000032932	0.530322036	0.02918227
Nomo1	ENSMUSG00000030835	-0.589027802	0.02918662
Gm15559	ENSMUSG00000086401	3.410278679	0.029186984
Cfap45	ENSMUSG00000026546	2.449139898	0.029220007
Gm9108	ENSMUSG00000115819	-0.687894581	0.029220007
Ier2	ENSMUSG00000053560	0.675114657	0.029220007
Parva	ENSMUSG00000030770	0.535310226	0.029245137
Spg21	ENSMUSG00000032388	0.645719232	0.029251184
Itgb7	ENSMUSG00000001281	1.230103486	0.029287274
Jarid2	ENSMUSG00000038518	-0.633762052	0.02938629
Oma1	ENSMUSG00000035069	-0.597772571	0.029482615
Gm5641	ENSMUSG00000069014	1.231526673	0.029514332
Gas5	ENSMUSG00000053332	0.753830605	0.02955137
Magi2	ENSMUSG00000040003	-0.943000753	0.029558352
Ctsf	ENSMUSG00000083282	-0.572714548	0.029558352
Stard13	ENSMUSG00000016128	-0.616144431	0.029785255
Ldb2	ENSMUSG00000039706	0.856268484	0.029785255
mt-Nd1	ENSMUSG00000064341	-0.647989177	0.029822476
Gm3531	ENSMUSG00000089782	0.793829139	0.029898256
Mtdh	ENSMUSG00000022255	0.525696027	0.029898595
Stx5a	ENSMUSG00000010110	0.534950398	0.029935329
Ccdc117	ENSMUSG00000020482	-0.533905324	0.029948155
M1ap	ENSMUSG00000030041	2.834493973	0.029978761
Cxcl1	ENSMUSG00000029380	-1.314838604	0.029981042
Gmpr	ENSMUSG00000000253	-0.486755009	0.030028139
Psma5	ENSMUSG00000068749	0.451392985	0.030130278
Lrrc8b	ENSMUSG00000070639	0.753545573	0.030141074
Amdhd2	ENSMUSG00000036820	0.726288342	0.030141074
Plaur	ENSMUSG00000046223	1.050883868	0.030145727

Zfp330	ENSMUSG00000031711	0.494133703	0.030261613
Crmp1	ENSMUSG00000029121	2.305302953	0.030261613
Sdr39u1	ENSMUSG00000022223	-0.595897719	0.030341506
Cnr2	ENSMUSG00000062585	1.696661391	0.030361848
Stap2	ENSMUSG00000038781	0.606393582	0.030379355
Hic1	ENSMUSG00000043099	-0.693053887	0.030405086
Lsamp	ENSMUSG00000061080	-0.985339441	0.030416387
Rps6kb2	ENSMUSG00000024830	-0.621386368	0.030423208
Azin1	ENSMUSG00000037458	0.416224961	0.030438593
Gngt2	ENSMUSG00000038811	0.697254618	0.030447504
Txndc12	ENSMUSG00000028567	0.524222573	0.030447504
Trim68	ENSMUSG00000073968	-0.742254855	0.030447504
Kifc1	ENSMUSG00000079553	1.592235228	0.030447504
Cdkn2b	ENSMUSG00000073802	1.522291848	0.030491895
Bcl2a1a	ENSMUSG00000102037	2.07192298	0.030491895
Pnpla8	ENSMUSG00000036257	-0.468329509	0.030561432
Golgb1	ENSMUSG00000034243	-0.599031076	0.030561432
Rdh11	ENSMUSG00000066441	0.86002157	0.030561432
Srgn	ENSMUSG00000020077	0.691558161	0.030561432
Pcgf6	ENSMUSG00000025050	0.978585095	0.030567208
Kcnj3	ENSMUSG00000026824	-1.87519549	0.030567208
2810004N23Rik	ENSMUSG00000031984	-0.439721588	0.030571598
Fam49b	ENSMUSG00000022378	0.535909262	0.030606612
Camkk1	ENSMUSG00000020785	1.057782721	0.030670983
Prickle1	ENSMUSG00000036158	-0.711314044	0.030681865
Ano5	ENSMUSG00000055489	-3.32351678	0.03074074
Slc22a23	ENSMUSG00000038267	-1.179431392	0.030804941
Sfxn4	ENSMUSG00000063698	-0.808222105	0.030822065
1010001B22Rik	ENSMUSG00000097863	-2.359639105	0.030839569
Slc5a3	ENSMUSG00000089774	1.21029388	0.030887732
Gm15163	ENSMUSG00000085711	-0.84956581	0.030887732
Lrrc27	ENSMUSG00000015980	-0.687221074	0.031073486
Lmod2	ENSMUSG00000029683	0.448097601	0.031204551
Gm33023	ENSMUSG00000110652	-1.693961947	0.031208519
Ppan	ENSMUSG00000004100	0.725286795	0.031270696
Sdad1	ENSMUSG00000029415	0.660901168	0.031270696
Nat8f3	ENSMUSG00000051262	-5.094750106	0.031358705
Alox5ap	ENSMUSG00000060063	0.76453038	0.031368413
Phax	ENSMUSG00000008301	0.533405483	0.031384995
Dpp9	ENSMUSG00000001229	-0.526886619	0.03143399

Mum1	ENSMUSG00000020156	-0.64988698	0.031482395
Wtip	ENSMUSG00000036459	0.607055116	0.031482395
Col22a1	ENSMUSG00000079022	2.677009023	0.031482395
Gpd2	ENSMUSG00000026827	-0.818398914	0.031482395
Uqcrc2	ENSMUSG00000030884	-0.505509374	0.031482395
Hspa1a	ENSMUSG00000091971	2.36887584	0.031508045
Gm43823	ENSMUSG00000106224	-1.872531939	0.031508045
Rpl34-ps1	ENSMUSG00000068396	0.781302571	0.031537527
Gm42732	ENSMUSG00000107331	-1.514467505	0.031546534
Timm21	ENSMUSG00000024645	-0.609278873	0.031567577
Gm11263	ENSMUSG00000083496	0.992493681	0.031604423
Tnxb	ENSMUSG00000033327	-0.516596437	0.031606289
Rere	ENSMUSG00000039852	-0.49695262	0.031620727
Tnfaip6	ENSMUSG00000053475	1.243970999	0.031674859
Pik3r4	ENSMUSG00000032571	-0.661193999	0.031674859
Fbxo33	ENSMUSG00000035329	0.643884056	0.031912206
Mrln	ENSMUSG00000019933	-2.85871051	0.031915546
Slc52a3	ENSMUSG00000027463	1.348990004	0.03208223
Igtp	ENSMUSG00000078853	-0.661449321	0.03208223
Rgs7	ENSMUSG00000026527	-1.299862916	0.032140684
Rhoa	ENSMUSG00000007815	0.406922999	0.032174055
BC004004	ENSMUSG00000052712	-0.47081096	0.032266671
Mill2	ENSMUSG00000040987	-0.615942838	0.032274526
P3h3	ENSMUSG00000023191	0.562811092	0.032290719
Arl6	ENSMUSG00000022722	0.646618285	0.032305951
Lrr1	ENSMUSG00000034883	4.518490004	0.032374207
Soat1	ENSMUSG00000026600	0.622139978	0.032431299
Sdhc	ENSMUSG00000058076	-0.471957391	0.032431299
Rpl10	ENSMUSG00000008682	0.489917499	0.032444517
Smtnl2	ENSMUSG00000045667	-0.502134786	0.032516711
Kctd10	ENSMUSG00000001098	0.588543545	0.032551163
Aunip	ENSMUSG00000078521	4.060961457	0.032647409
Hnrnpa0	ENSMUSG00000007836	0.424754129	0.032650114
Usp9x	ENSMUSG00000031010	-0.631795605	0.032747132
Lrrc20	ENSMUSG00000037151	-0.647870145	0.03275912
Sft2d1	ENSMUSG00000073468	0.604500848	0.032805124
Mef2d	ENSMUSG00000001419	-0.510654446	0.032825307
Rpl41	ENSMUSG00000093674	0.680478666	0.032825307
Ralb	ENSMUSG00000004451	0.462021865	0.032825307
Ttc1	ENSMUSG00000041278	0.442548646	0.032825307

Ppm1b	ENSMUSG00000061130	-0.510650312	0.032825307
Arl1	ENSMUSG00000060904	0.396536423	0.032962483
Ago4	ENSMUSG00000042500	-0.834707163	0.033020817
Tmed2	ENSMUSG00000029390	0.387991343	0.033104074
1010001N08Rik	ENSMUSG00000097222	-0.849222709	0.033155093
Borcs5	ENSMUSG00000042992	-0.604837873	0.033174468
Pnrc1	ENSMUSG00000040128	0.446878742	0.033192712
Mrto4-ps2	ENSMUSG00000091105	4.943898595	0.033194467
Nat8f4	ENSMUSG00000068299	-2.383384085	0.033220752
Nabp1	ENSMUSG00000026107	0.461928509	0.033235232
Ipo7	ENSMUSG00000066232	0.468765574	0.033300351
Cep104	ENSMUSG00000039523	-0.572847406	0.033310601
Zscan26	ENSMUSG00000022228	-0.651510123	0.033481678
Zmym5	ENSMUSG00000040123	-0.504896305	0.033539213
Vcpkmt	ENSMUSG00000049882	0.742840305	0.033629832
Vasp	ENSMUSG00000030403	0.517352763	0.033704721
Dpysl5	ENSMUSG00000029168	-2.026755685	0.033758708
Dhx57	ENSMUSG00000035051	-0.780873667	0.03381636
A330074K22Rik	ENSMUSG00000097960	2.810701703	0.03387266
Angptl1	ENSMUSG00000033544	-1.517838707	0.033960214
Eps15l1	ENSMUSG00000006276	-0.539657253	0.03407361
Plscr2	ENSMUSG00000032372	0.538870734	0.03407361
4930507D05Rik	ENSMUSG00000097129	5.061022543	0.03409297
Lin7b	ENSMUSG00000003872	-2.845683248	0.03409297
Arl11	ENSMUSG00000043157	0.975006973	0.034149945
Lrrc8c	ENSMUSG00000054720	0.647268448	0.034149945
Gm8394	ENSMUSG00000050490	0.763995808	0.03420743
Tagap	ENSMUSG00000033450	1.273912919	0.034217272
Stag2	ENSMUSG00000025862	0.461598627	0.034265474
1110059E24Rik	ENSMUSG00000035171	0.567563167	0.034330041
Eif3h	ENSMUSG00000022312	0.472550685	0.034346067
Kif1b	ENSMUSG00000063077	-0.573475921	0.034350076
Cep83	ENSMUSG00000020024	0.513036167	0.034350076
Metap1d	ENSMUSG00000041921	-0.54789527	0.034426662
Olfm1	ENSMUSG00000026833	0.763867304	0.034433222
Mefv	ENSMUSG00000022534	2.721913804	0.034459179
Dstn	ENSMUSG00000015932	0.479638378	0.034459179
Ppef1	ENSMUSG00000062168	4.437796209	0.034459179
Zap70	ENSMUSG00000026117	2.762161142	0.034684819
Cbx3	ENSMUSG00000029836	0.583173594	0.034753277

Clk1	ENSMUSG00000026034	-0.618746596	0.034789058
Mttp	ENSMUSG00000028158	-0.954359027	0.034856405
Gda	ENSMUSG00000058624	0.554956689	0.034856405
Ythdf2	ENSMUSG00000040025	0.513336556	0.034953361
Gtf2f1	ENSMUSG00000002658	0.530343416	0.034980331
Cd8a	ENSMUSG00000053977	3.010129326	0.034983286
Lpcat3	ENSMUSG00000004270	-0.597951119	0.035055057
9130015G15Rik	ENSMUSG00000108950	2.708691081	0.035104574
Rab3ip	ENSMUSG00000064181	0.454457517	0.035139502
Atp5g3	ENSMUSG00000018770	-0.483578596	0.035190744
Baiap2	ENSMUSG00000025372	0.625238855	0.035196513
Lcn2	ENSMUSG00000026822	0.736834284	0.035273116
Gm43980	ENSMUSG00000107962	-5.004133089	0.035434502
Polr1e	ENSMUSG00000028318	0.636847092	0.035459785
Calhm6	ENSMUSG00000046031	1.571071496	0.035500423
Slco2b1	ENSMUSG00000030737	-0.564619404	0.035500423
Syt7	ENSMUSG00000024743	-0.769362931	0.035750736
Vil1	ENSMUSG00000026175	-5.006532326	0.035774904
Hspa14	ENSMUSG00000109865	0.5129426	0.035790258
Clec4a2	ENSMUSG00000030148	0.947047251	0.035816316
Spred1	ENSMUSG00000027351	0.534231506	0.035831776
Opn4	ENSMUSG00000021799	-0.978945956	0.035875807
Ifi203	ENSMUSG00000039997	0.535725665	0.035875807
Kcnk3	ENSMUSG00000049265	-0.626099537	0.035875807
Sesn2	ENSMUSG00000028893	1.02159761	0.035911803
Fbrsl1	ENSMUSG00000043323	-0.54189252	0.035938009
Eif3m	ENSMUSG00000027170	0.41701591	0.036089315
Alms1	ENSMUSG00000063810	-1.509315599	0.036092229
Rgs3	ENSMUSG00000059810	-0.531855103	0.03611529
Flrt2	ENSMUSG00000047414	-0.678865272	0.036229047
Tek	ENSMUSG00000006386	-0.450390038	0.036229047
Klhl10	ENSMUSG00000001558	-3.496777556	0.036229047
Pdlim1	ENSMUSG00000055044	0.434354939	0.036262148
Phf24	ENSMUSG00000036062	-1.007970936	0.036380684
Tet1	ENSMUSG00000047146	-1.348241742	0.036503301
Hist1h2ao	ENSMUSG00000094248	4.95127035	0.03665609
Slc2a8	ENSMUSG00000026791	-0.596910073	0.036712905
Ube2s	ENSMUSG00000060860	0.472548338	0.036749739
Adgrd1	ENSMUSG00000044017	-0.770956726	0.036763852
Gorasp2	ENSMUSG00000014959	0.439417377	0.036781358

Gm38388	ENSMUSG00000103215	-5.061711613	0.036814056
Mlh3	ENSMUSG00000021245	-0.726552637	0.036859396
Tmem41a	ENSMUSG00000022856	-0.632119396	0.03692677
Clca3a1	ENSMUSG00000056025	1.018634292	0.036932406
Gm12551	ENSMUSG00000081169	4.974106812	0.037165653
Prim2	ENSMUSG00000026134	0.919703227	0.037168064
Ankrd63	ENSMUSG00000078137	-1.604341825	0.037265772
Bcl7a	ENSMUSG00000029438	-0.847558533	0.037274701
Itgb6	ENSMUSG00000026971	-1.297479304	0.03728427
Psmd10	ENSMUSG00000031429	0.53486917	0.037296488
Pigg	ENSMUSG00000029263	-0.967308159	0.03744465
Smug1	ENSMUSG00000036061	-0.666323524	0.03748594
Dnajc16	ENSMUSG00000040697	-0.642722621	0.037533868
Rnmt	ENSMUSG00000009535	0.520851394	0.037533868
Got2	ENSMUSG00000031672	-0.433762486	0.037555515
Nmral1	ENSMUSG00000063445	0.832988341	0.037555515
Mical1	ENSMUSG00000019823	0.872293202	0.037556723
Gm13841	ENSMUSG00000083833	0.612524332	0.037643571
Atxn2	ENSMUSG00000042605	-0.600895425	0.037651233
Bag5	ENSMUSG00000049792	0.485196891	0.037835632
Myot	ENSMUSG00000024471	-0.675807451	0.037848391
Amot	ENSMUSG00000041688	-0.637982914	0.037868704
Arhgef11	ENSMUSG00000041977	-0.765119784	0.037868704
BC048679	ENSMUSG00000061877	-5.01637595	0.037893128
Bub3	ENSMUSG00000066979	0.490786114	0.037943985
Adgrg3	ENSMUSG00000060470	1.000782854	0.038004011
Bloc1s5	ENSMUSG00000038982	0.484402381	0.038107077
Pdcl3	ENSMUSG00000026078	0.429460719	0.038171151
Taf1a	ENSMUSG00000072258	-0.60056576	0.038216222
Mrps36	ENSMUSG00000061474	-0.540460232	0.038225818
Ywhaq-ps3	ENSMUSG00000080902	1.367220631	0.038225818
Nap1l3	ENSMUSG00000055733	-1.549550546	0.038225818
Snrpd1	ENSMUSG00000002477	0.609499166	0.038250668
Mmp9	ENSMUSG00000017737	-1.39488616	0.038262398
Gpr141	ENSMUSG00000053101	2.610289186	0.038276234
Mef2c	ENSMUSG00000005583	-0.529329776	0.038347333
Zmat2	ENSMUSG00000001383	0.455569856	0.038347333
Cenpt	ENSMUSG00000036672	0.89209119	0.038347333
Mgst3	ENSMUSG00000026688	-0.437911005	0.038393792
Itgax	ENSMUSG00000030789	1.66902535	0.038410518

Ift20	ENSMUSG00000001105	0.424861953	0.038414363
Gm43618	ENSMUSG00000105403	-2.84332728	0.038546714
Gria3	ENSMUSG00000001986	0.964047317	0.038546714
Dhfr	ENSMUSG00000021707	0.792404947	0.038546714
Ncoa2	ENSMUSG00000005886	-0.554719048	0.038556754
Slc25a37	ENSMUSG00000034248	-0.658758743	0.038556754
Atp6v0a2	ENSMUSG00000038023	-0.532485021	0.038624778
Fgf11	ENSMUSG00000042826	-1.081057639	0.038692225
Mrps18a	ENSMUSG00000023967	0.411612007	0.038724585
Card10	ENSMUSG00000033170	-0.601903817	0.038753449
Lvrn	ENSMUSG00000024481	-1.760715048	0.038886588
Calhm5	ENSMUSG00000049872	0.991217418	0.038981854
Iqub	ENSMUSG00000046192	-5.015882471	0.038981854
Slc2a12	ENSMUSG00000037490	-1.048721908	0.038981854
Wdr35	ENSMUSG00000066643	-0.612124352	0.039000508
Mxd4	ENSMUSG00000037235	0.484664813	0.039008643
Dnaja3	ENSMUSG00000004069	-0.471316738	0.039008643
Esrrb	ENSMUSG00000021255	-0.588411672	0.039008643
Pask	ENSMUSG00000026274	2.354314658	0.039011927
Ctsd	ENSMUSG00000007891	0.416512164	0.039041013
Ppp1r2	ENSMUSG00000047714	0.388720614	0.03904653
Cyhr1	ENSMUSG00000053929	-0.414383885	0.039111631
Phf11a	ENSMUSG00000044703	1.790213308	0.039143586
Hnrnpf	ENSMUSG00000042079	0.479637239	0.039178092
Rsl1d1	ENSMUSG00000005846	0.492017742	0.039269768
Ubash3a	ENSMUSG00000042345	4.873372386	0.039280015
Mlxip	ENSMUSG00000038342	-0.557898393	0.039308801
Clybl	ENSMUSG00000025545	-0.451217292	0.03937964
Gm17066	ENSMUSG00000091509	0.942600366	0.039420816
Rab5b	ENSMUSG00000000711	-0.442516514	0.039478381
Nckap5	ENSMUSG00000049690	-1.155059298	0.039743574
U2af1	ENSMUSG00000061613	0.493765617	0.03974411
Zranb1	ENSMUSG00000030967	0.615247778	0.039754587
Lpin1	ENSMUSG00000020593	-0.654112358	0.039802671
Cyb561d2	ENSMUSG00000037190	0.679120245	0.039846745
Herc6	ENSMUSG00000029798	0.941029998	0.039846745
Ccdc146	ENSMUSG00000064280	-1.012660634	0.039885094
mt-Th	ENSMUSG00000064364	-3.424738485	0.03989479
Pacsin2	ENSMUSG00000016664	-0.426747825	0.039905414
Afg1l	ENSMUSG00000038302	-0.538935421	0.039909777

Tceal1	ENSMUSG00000049536	-0.566504891	0.03991029
E2f2	ENSMUSG00000018983	1.449084522	0.039998043
Ace	ENSMUSG00000020681	0.572899063	0.039998043
Cops2	ENSMUSG00000027206	0.414414838	0.039998043
2900041M22Rik	ENSMUSG00000054418	1.137512707	0.039998043
Fbn2	ENSMUSG00000024598	1.727887207	0.039998043
Slc12a2	ENSMUSG00000024597	0.582892727	0.040021882
Fam160a1	ENSMUSG00000051000	-0.545929548	0.040040175
Ppp1r18	ENSMUSG00000034595	0.57659067	0.040040175
Sec61g	ENSMUSG00000078974	0.636307099	0.040060512
Igf2r	ENSMUSG00000023830	-0.500979476	0.040079393
Gosr2	ENSMUSG00000020946	0.528983579	0.040079393
Rplp0	ENSMUSG00000067274	0.432432543	0.040079393
Kif18a	ENSMUSG00000027115	1.217995094	0.040079393
Sfpq	ENSMUSG00000028820	0.481128061	0.040095817
Casp8	ENSMUSG00000026029	0.61228695	0.040127353
AC133498.1	ENSMUSG00000114053	-0.818165721	0.040127353
Idh3g	ENSMUSG00000002010	-0.399941256	0.040127353
Gm16271	ENSMUSG00000089656	-3.852665688	0.040127353
Col4a5	ENSMUSG00000031274	0.63121573	0.04012796
Gm8818	ENSMUSG00000079138	4.973507265	0.040132019
Gprasp2	ENSMUSG00000072966	-1.474785687	0.040369737
Tbxa2r	ENSMUSG00000034881	1.170593336	0.040437232
Slc16a2	ENSMUSG00000033965	-0.641897769	0.040503327
Mylip	ENSMUSG00000038175	-0.566855601	0.040503327
Trim27	ENSMUSG00000021326	0.499413607	0.040550608
Pappa	ENSMUSG00000028370	1.666308374	0.040554573
9130401M01Rik	ENSMUSG00000101892	0.687807566	0.040600409
Derl2	ENSMUSG00000018442	0.437649605	0.040642497
Rtcb	ENSMUSG00000001783	0.427368157	0.040668047
Klrk1	ENSMUSG00000030149	2.162851291	0.040668047
Pecr	ENSMUSG00000026189	-0.642585086	0.040724303
Luzp2	ENSMUSG00000063297	-5.028787081	0.04073791
Ift46	ENSMUSG00000002031	0.527581419	0.04073791
Actg2	ENSMUSG00000059430	1.756348133	0.040743994
Ednra	ENSMUSG00000031616	-0.667798298	0.040831218
Smim15	ENSMUSG00000071180	0.49224689	0.040867021
Letm2	ENSMUSG00000037363	-1.004269348	0.040867021
Ggt5	ENSMUSG00000006344	-0.758929021	0.040867021
Dnajc4	ENSMUSG00000024963	-0.432579024	0.040867021

Gm15535	ENSMUSG00000085527	-2.364016377	0.040896202
Zfp174	ENSMUSG00000054939	-2.004633577	0.040896202
Gm867	ENSMUSG00000050157	4.328957225	0.040898546
Agk	ENSMUSG00000029916	-0.718453437	0.040898546
Dnajc5b	ENSMUSG00000027606	-4.483485811	0.040898546
Dpp3	ENSMUSG00000063904	0.459983941	0.040898546
Gtf3c1	ENSMUSG00000032777	-0.483239835	0.041021586
Zfp791	ENSMUSG00000074194	-1.319929358	0.041025847
Cobll1	ENSMUSG00000034903	-0.557936317	0.041025847
Dhps	ENSMUSG00000060038	0.467538336	0.041025847
Plk5	ENSMUSG00000035486	-2.037253522	0.041025847
Ddx6	ENSMUSG00000032097	-0.434924509	0.041025847
Pbld1	ENSMUSG00000112129	-5.028172184	0.041045212
Cers4	ENSMUSG00000008206	-0.488219313	0.041103535
Cct7	ENSMUSG00000030007	0.446373308	0.041202343
Akr7a5	ENSMUSG00000028743	-0.405396852	0.041202343
Snhg6	ENSMUSG00000098234	0.717690783	0.041332893
Klhl33	ENSMUSG00000090799	-1.968077367	0.041332893
Ube2e3	ENSMUSG00000027011	-0.458817705	0.04142081
Rhog	ENSMUSG00000073982	0.577248465	0.04150698
Atp6v0b	ENSMUSG00000033379	0.421737262	0.041568177
Spidr	ENSMUSG00000041974	0.919424913	0.041568177
Fabp3-ps1	ENSMUSG00000056366	-0.569370586	0.041608062
Arhgef18	ENSMUSG00000004568	-0.626784009	0.041641911
Scp2	ENSMUSG00000028603	-0.461837649	0.041664175
Fxr2	ENSMUSG00000018765	-0.397470446	0.041860039
1810021B22Rik	ENSMUSG00000087331	-1.196250419	0.041962464
Slc25a22	ENSMUSG00000019082	-0.772550827	0.041984214
B430212C06Rik	ENSMUSG00000046415	-1.544517204	0.042003372
Slc22a5	ENSMUSG00000018900	-0.592203145	0.042027017
Focad	ENSMUSG00000038368	-0.776468474	0.042076493
Sirt4	ENSMUSG00000029524	-0.715551364	0.04208669
Gm29560	ENSMUSG00000101641	-3.486638852	0.042092957
4930458D05Rik	ENSMUSG00000087611	-1.555589037	0.042110586
Gm7665	ENSMUSG00000063628	1.224040127	0.042110586
Casz1	ENSMUSG00000028977	-0.766583679	0.042110586
Smim24	ENSMUSG00000078439	-1.061267557	0.04232472
Emc8	ENSMUSG00000031819	0.474768301	0.042325291
Scn5a	ENSMUSG00000032511	-0.619047764	0.042343158
Phf11b	ENSMUSG00000091649	0.769284478	0.042381904

Map10	ENSMUSG00000050930	-0.858621879	0.042432114
Pick1	ENSMUSG00000068206	-0.821118431	0.042544783
Fbxl17	ENSMUSG00000023965	-0.498404897	0.042845873
Runx1t1	ENSMUSG00000006586	-1.20978313	0.0428772
Fam241b	ENSMUSG00000020083	-0.971371082	0.043021152
Axl	ENSMUSG00000002602	0.476305723	0.043028626
Myo9b	ENSMUSG00000004677	0.552244025	0.043038653
Pgf	ENSMUSG00000004791	-0.755965092	0.043038653
Surf4	ENSMUSG00000014867	0.438558339	0.043150213
Hcst	ENSMUSG00000064109	1.671785951	0.043257596
Fry	ENSMUSG00000056602	-0.618819738	0.04325837
Hint3	ENSMUSG00000019791	-0.431788196	0.043355728
Acy1	ENSMUSG00000023262	-0.632114313	0.043467147
Rpl35	ENSMUSG00000062997	0.621961001	0.043467147
Nsd1	ENSMUSG00000021488	-0.525150091	0.043478253
Csnk1a1	ENSMUSG00000024576	0.391855775	0.043486376
Sema3a	ENSMUSG00000028883	1.073037221	0.043575237
Il17rc	ENSMUSG00000030281	0.62963595	0.043671971
Gm36670	ENSMUSG00000109696	-1.29567791	0.04368179
Adam8	ENSMUSG00000025473	1.369723371	0.04385619
Eme1	ENSMUSG00000039055	2.896387439	0.04385619
Chmp2b	ENSMUSG00000004843	0.412042139	0.04385619
Kansl1	ENSMUSG00000018412	-0.60491103	0.043872808
Fkbp8	ENSMUSG00000019428	0.389191384	0.043887614
Kif5c	ENSMUSG00000026764	1.419702276	0.043909149
Serpina3h	ENSMUSG00000041449	2.318606543	0.043937626
Eif3e	ENSMUSG00000022336	0.444032228	0.043981334
Apoo	ENSMUSG00000079508	-0.402928987	0.044082054
Rps27l	ENSMUSG00000036781	0.485414433	0.044082054
Usp1	ENSMUSG00000028560	0.473572006	0.044193112
mt-Ts2	ENSMUSG00000064365	-2.905944092	0.044193112
Synrg	ENSMUSG00000034940	-0.650602319	0.044193112
Malat1	ENSMUSG00000092341	-1.500169786	0.044223234
Gm49172	ENSMUSG00000115049	0.840610648	0.044223411
Nedd4	ENSMUSG00000032216	-0.416615293	0.04429857
Klc1	ENSMUSG00000021288	0.437982038	0.04429857
Gm14261	ENSMUSG00000085322	-0.962253954	0.044301639
Zfp991	ENSMUSG00000067916	0.676200251	0.044425386
Ylpm1	ENSMUSG00000021244	-0.629008166	0.044425386
Hsp25-ps1	ENSMUSG00000078915	2.321795153	0.044506612

Gfer	ENSMUSG00000040888	0.473841337	0.044506612
Fam117a	ENSMUSG00000038893	-0.719109684	0.044553424
Sel1l3	ENSMUSG00000029189	-2.185951162	0.044647792
Ptms	ENSMUSG00000030122	0.566642362	0.044816191
Gm30556	ENSMUSG00000115180	-1.394431645	0.044816191
Ppard	ENSMUSG00000002250	-0.550850217	0.044845757
Cenpn	ENSMUSG00000031756	1.476289358	0.044913614
Pygo1	ENSMUSG00000034910	-0.723130472	0.044969079
Stard9	ENSMUSG00000033705	-0.873728501	0.044969079
Dgkeos	ENSMUSG00000063109	-2.330569862	0.044987364
Aimp2	ENSMUSG00000029610	0.51650305	0.045002719
C1galt1c1	ENSMUSG00000048970	0.59498469	0.045084771
Hoxd8	ENSMUSG00000027102	1.253581896	0.04512603
Mgll	ENSMUSG00000033174	0.582787898	0.04512603
4930452B06Rik	ENSMUSG00000021747	-1.167256195	0.045159314
Chmp2a	ENSMUSG00000033916	0.380701038	0.045276262
Celsr2	ENSMUSG00000068740	-1.770525975	0.045276262
Pon2	ENSMUSG00000032667	0.477440836	0.045293569
Lypd2	ENSMUSG00000022595	-1.357829253	0.045329388
Tnik	ENSMUSG00000027692	-0.670417475	0.045394695
Arhgap21	ENSMUSG00000036591	-0.636531661	0.045524959
Stard7	ENSMUSG00000027367	-0.501716205	0.045573456
Crb2	ENSMUSG00000035403	-1.74101755	0.045573456
Vnn1	ENSMUSG00000037440	-2.117081585	0.045575973
Rsrc1	ENSMUSG00000034544	0.491837627	0.045592773
Cct4	ENSMUSG00000007739	0.417557754	0.045630816
Sp1	ENSMUSG00000001280	-0.554678287	0.045630816
Gm43813	ENSMUSG00000106636	4.406499603	0.045648999
Vav1	ENSMUSG00000034116	0.686742435	0.045660585
Tmem106b	ENSMUSG00000029571	-0.398408027	0.045712252
Gm11431	ENSMUSG00000087407	-4.928938293	0.045726336
Npm3	ENSMUSG00000056209	0.497681278	0.045765533
Rdh14	ENSMUSG00000020621	-0.49757455	0.045768969
Matr3	ENSMUSG00000037236	0.403652732	0.045823212
Dsn1	ENSMUSG00000027635	1.077071619	0.045823212
Tpsb2	ENSMUSG00000033825	0.941401699	0.045908756
Rps27	ENSMUSG00000090733	0.687856195	0.045963356
Dock3	ENSMUSG00000039716	-4.980194835	0.04607839
Cxxc5	ENSMUSG00000046668	-0.535139297	0.046120832
Eya1	ENSMUSG00000025932	-1.986444569	0.046134647

Gm4924	ENSMUSG00000073427	1.398242619	0.046136658
Stk3	ENSMUSG00000022329	0.649834007	0.046145342
Faap24	ENSMUSG00000030493	0.982011757	0.046348342
Pard6a	ENSMUSG00000005699	0.607347747	0.046382702
Ptov1	ENSMUSG00000038502	-0.409981233	0.046415031
Mks1	ENSMUSG00000034121	-1.194966464	0.046433828
Sec16a	ENSMUSG00000026924	-0.599162852	0.046439095
Rnf167	ENSMUSG00000040746	-0.482114033	0.046439095
Bzw2	ENSMUSG00000020547	-0.499964782	0.046439095
Lrrn2	ENSMUSG00000026443	0.907637001	0.046481339
Ptpa	ENSMUSG00000039515	0.399158227	0.046575037
Ano8	ENSMUSG00000034863	-0.68888599	0.046575037
Tor1a	ENSMUSG00000026849	0.543656173	0.046575037
Extl1	ENSMUSG00000028838	-0.73974013	0.046575037
Stt3b	ENSMUSG00000032437	0.451912959	0.046642207
Gm26772	ENSMUSG00000097136	4.270436556	0.0467834
Mrps24	ENSMUSG00000020477	-0.416259501	0.0467834
Arhgap28	ENSMUSG00000024043	-1.111424494	0.046833514
Pter	ENSMUSG00000026730	-0.595796011	0.046886549
Cbr4	ENSMUSG00000031641	-0.476591873	0.046886549
Ndufa3	ENSMUSG00000035674	-0.658293158	0.047002519
Stradb	ENSMUSG00000026027	-0.426895867	0.047018098
Cpn2	ENSMUSG00000023176	-2.717262793	0.047060913
Usp16	ENSMUSG00000025616	0.442209293	0.047149416
Gmds	ENSMUSG00000038372	0.656626083	0.047236054
Atp6v0d2	ENSMUSG00000028238	2.022723207	0.047319377
Ypel2	ENSMUSG00000018427	-0.776638498	0.047364103
Taz	ENSMUSG00000009995	-0.590295361	0.047584894
Pdcd1	ENSMUSG00000026285	3.31146943	0.047606499
Rnf126	ENSMUSG00000035890	0.451202605	0.047633914
Plbd1	ENSMUSG00000030214	-0.48954994	0.047642049
Hipk3	ENSMUSG00000027177	-0.499909971	0.04775647
Cyp2b10	ENSMUSG00000030483	-2.566054262	0.047803543
Tap1	ENSMUSG00000037321	0.677978139	0.047856875
Gm3756	ENSMUSG00000091639	0.944740722	0.047856875
Afg3l2	ENSMUSG00000024527	0.498997983	0.047878496
Mrpl41	ENSMUSG00000036850	-0.56024256	0.047888742
D530033B14Rik	ENSMUSG00000108332	-2.299176488	0.047915341
Ddias	ENSMUSG00000030641	1.579804848	0.048018762
Tcf24	ENSMUSG00000099032	1.670876257	0.048048933

Irf4	ENSMUSG00000021356	-1.349359345	0.048229004
Gm7658	ENSMUSG00000082935	0.68756525	0.048329826
Apex2	ENSMUSG00000025269	-0.859199035	0.048357925
Zcchc3	ENSMUSG00000074682	-0.648259563	0.048357925
Gm49085	ENSMUSG00000115249	4.84378008	0.048357925
Lrfn4	ENSMUSG00000045045	-0.672375481	0.048357925
Arhgef15	ENSMUSG00000052921	-0.598167365	0.048357925
Ahcy	ENSMUSG00000027597	0.50176959	0.048406242
Ppp1r14b	ENSMUSG00000056612	0.452252991	0.048415832
Rcc1	ENSMUSG00000028896	0.701587698	0.048607252
Akt3	ENSMUSG00000019699	0.548118551	0.048656755
Smyd1	ENSMUSG00000055027	-0.473759606	0.048656755
Eif4a2	ENSMUSG00000022884	-0.475884366	0.048739275
Otub1	ENSMUSG00000024767	0.43891953	0.048905268
Crocc	ENSMUSG00000040860	-1.207012571	0.048929945
Cmtm3	ENSMUSG00000031875	0.514788365	0.048940846
Ecsit	ENSMUSG00000066839	-0.472868588	0.048993674
Rnf123	ENSMUSG00000041528	-0.535671253	0.049035111
Rexo1	ENSMUSG00000047417	-0.634737333	0.049157274
Sncaip	ENSMUSG00000024534	-0.67538239	0.049253706
Dcaf10	ENSMUSG00000035572	0.58597458	0.049315745
Ttc39a	ENSMUSG00000028555	-1.664958798	0.049315745
Gm15956	ENSMUSG00000086935	-3.081867457	0.049315745
Chd2	ENSMUSG00000078671	-0.643361951	0.049315745
Fam83e	ENSMUSG00000054161	-4.890735554	0.049318177
Nop16	ENSMUSG00000025869	0.537900196	0.049374178
9430091E24Rik	ENSMUSG00000084808	-1.084782866	0.049384338
Plekhh1	ENSMUSG00000060716	-2.90819733	0.049404902
Fcer1g	ENSMUSG00000058715	0.590833054	0.049428388
Pa2g4	ENSMUSG00000025364	0.437290551	0.049459502
Orai2	ENSMUSG00000039747	1.357157626	0.049494745
Tbl3	ENSMUSG00000040688	0.575598944	0.049702161
Mrpl39	ENSMUSG00000022889	-0.453209028	0.049710398
Cebpzos	ENSMUSG00000062691	-0.669999705	0.049710398
Klhl2	ENSMUSG00000031605	0.609737655	0.049774062
Gm11585	ENSMUSG00000082045	0.795593916	0.049787429
A430033K04Rik	ENSMUSG00000056014	-1.027424618	0.049787429
Rasl10a	ENSMUSG00000034209	-1.066672085	0.049787429
6030458C11Rik	ENSMUSG00000022195	-0.550979979	0.049885014
C330013E15Rik	ENSMUSG00000097093	-1.477784995	0.049932095

Figure S1. Expression abundance of SLC40A1 in different tissues of mice.

(A) Western blot analysis of SLC40A1 expression in wild-type mice's heart, liver, spleen, lung, kidney, and small intestine, normalized to β-actin. The data is presented as the mean value plus or minus the standard error of the mean (n = 4).

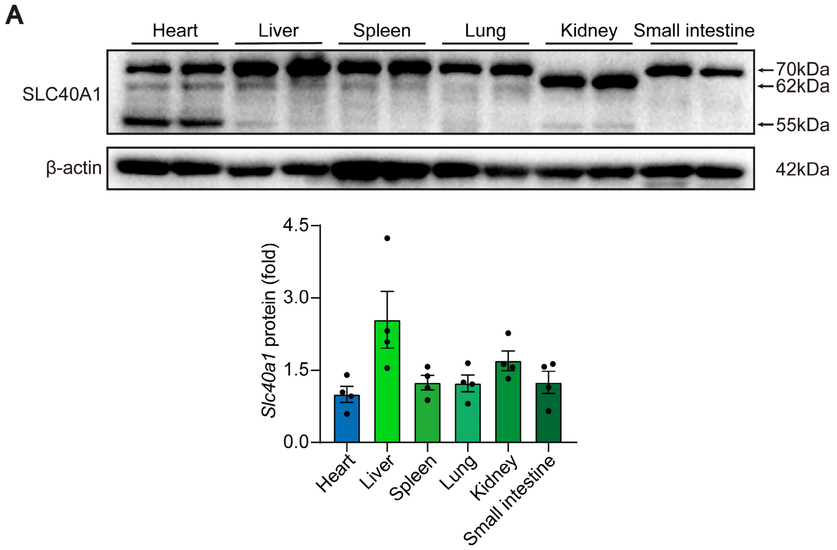

Figure S2. Identification of mice genotypes.

(A) The genotypes of Rosa26-LSL (LoxP-Stop-LoxP)-*Slc40a1* and *Myh6*-Cre were determined using PCR and gel electrophoresis. WT, wild type; MT, mutant.

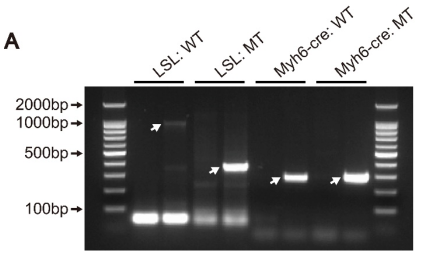

Figure S3. Increased heart weight in TG mice.

(A) Measurements of body weight and heart/body weight ratios were taken for both TG and NTG mice. The data is presented as the mean value plus or minus the standard error of the mean (n = 8). The data is presented as the mean value plus or minus the standard error of the mean. Statistical significance is denoted by *p < 0.05.

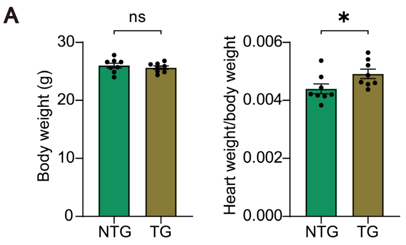

Figure S4. There were notable alterations observed in various pathways that are closely linked to mitochondrial function, ROS production, and apoptosis in TG mice.

(A) GO analysis was conducted to identify up- and down-regulated pathways in the hearts of TG mice compared to NTG

mice. The top 10 pathways in the biological process, cellular component, and molecular function classifications are presented.

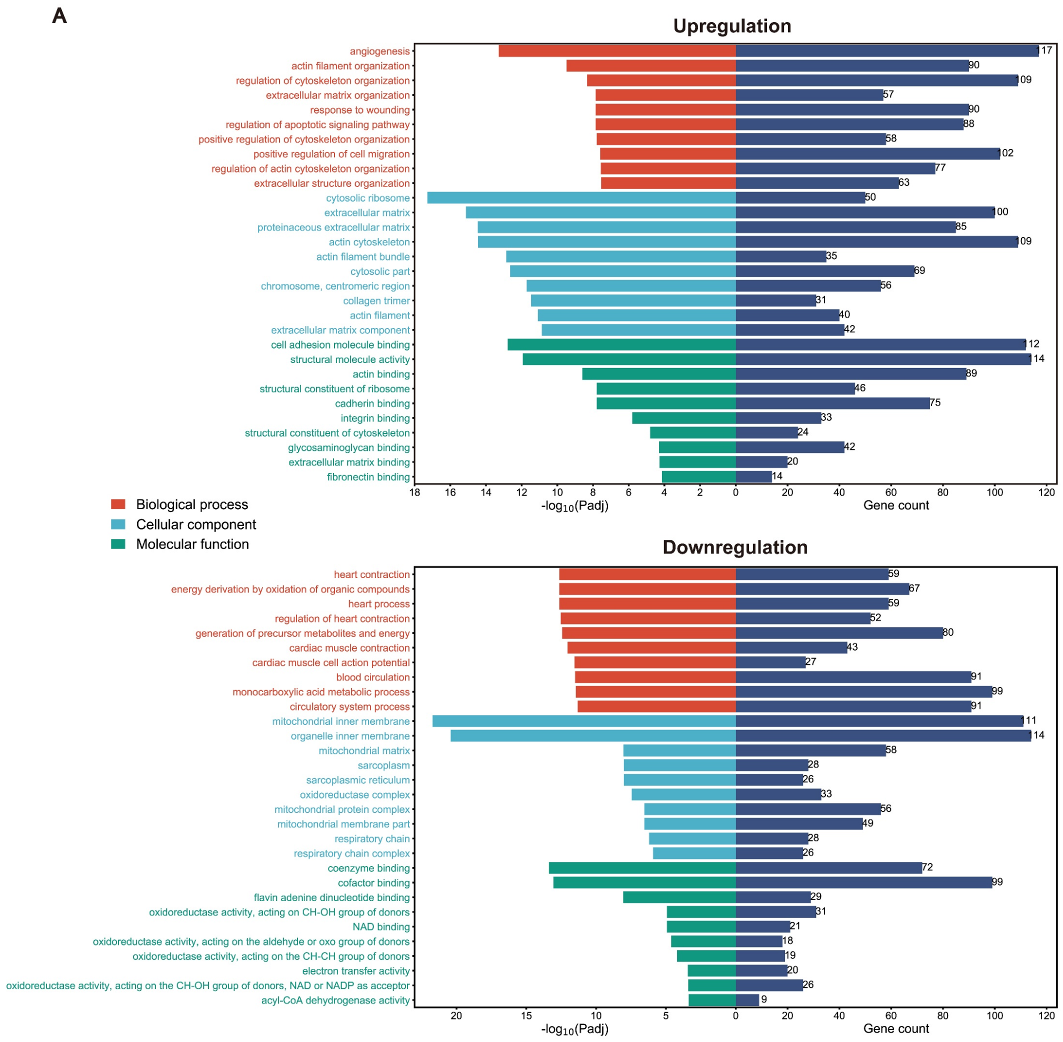

Figure S5. There was an absence of substantial alteration in the myocardial expression of crucial genes associated with ferroptosis in TG mice.

(A) The mRNA sequencing results revealed no differential expression of myocardial *Gpx4*, *Acsl4*, *Slc7a11*, *Alox15*, *Coq10a*, *Coq10b*, and *Ncoa4* in TG mice compared to NTG mice (n = 3). (B) Western blot analysis of Gpx4 expression of myocardial in TG mice compared to NTG mice. The data is presented as the mean value plus or minus the standard error of the mean (n = 4).

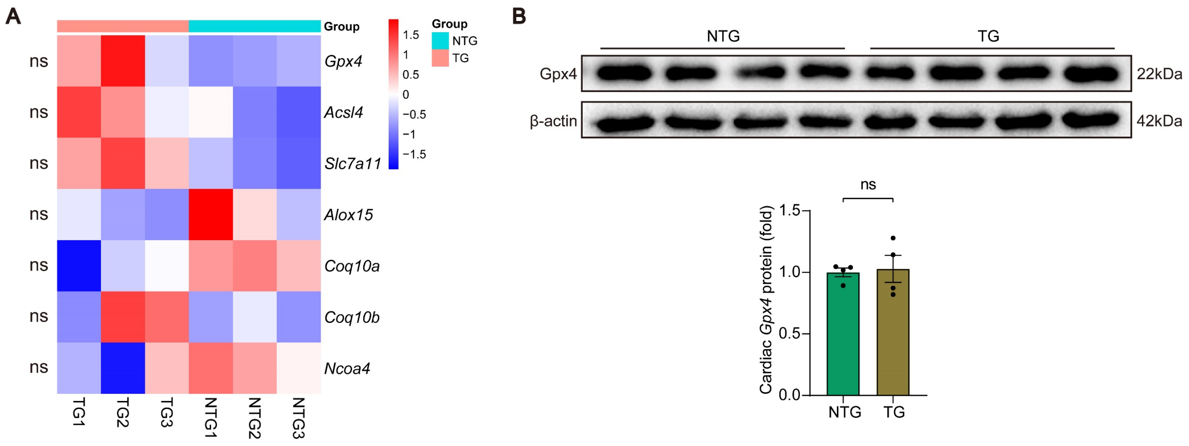

Figure S6. The effectiveness of SLC40A1 overexpression was assessed.

(A) NRMCs *Slc40a1* protein was measured using western blot analysis, normalized to β-actin (n = 3). The data is presented as the mean value plus or minus the standard error of the mean. Statistical significance is denoted by **p < 0.01.

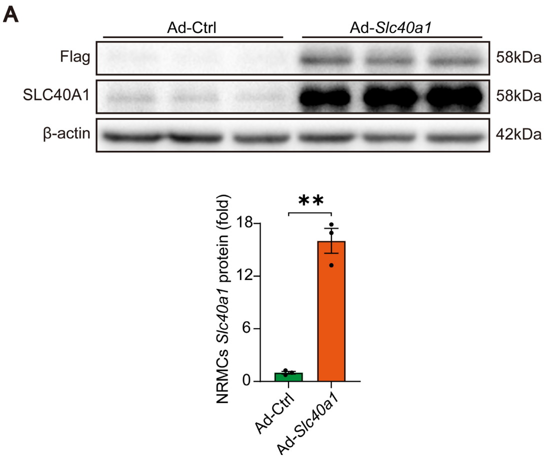

Figure S7. *Slc40a1* were up-regulated in human heart with heart failure.

(A) *Slc40a1* mRNA levels in the heart samples of non-failing (NF) subjects (n = 15) and dilated cardiomyopathy (DCM) patients (n =13) based on RNA-seq data. (B) *Slc40a1* mRNA levels in the heart samples of non-failing (NF) subjects (n = 11), idiopathic dilated cardiomyopathy (IDC) patients (n =15), and ischemic cardiomyopathy (ICM) patients (n =11). The data is presented as the mean value plus or minus the standard error of the mean. Statistical significance is denoted by *p < 0.05 and **p < 0.01.

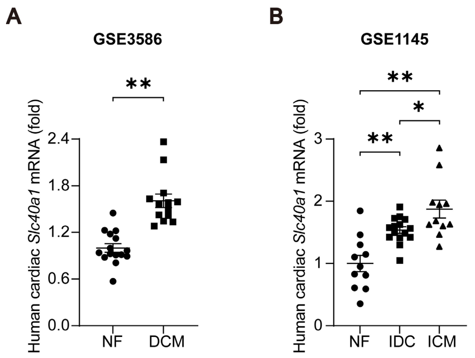

Figure S8. The effectiveness of SLC40A1 knock-down was assessed.

(A) NRMCs *Slc40a1* protein was measured using western blot analysis, normalized to β-actin (n = 3). We used Ad-sh*Slc40a1*-3 for further research. The data is presented as the mean value plus or minus the standard error of the mean. Statistical significance is denoted by *p < 0.05 and **p < 0.01.

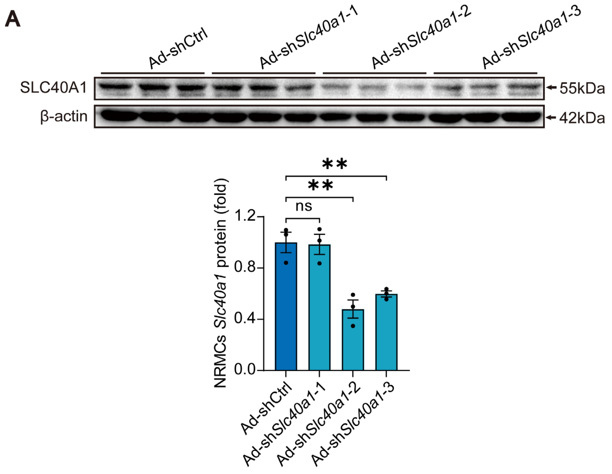

Figure S9. Knockdown of Steap4 in cardiomyocytes resulted in improved mitochondrial morphology, inhibition of NADPH content decrease, and reduced apoptosis in TG mice.

(A) Electron micrographs of heart tissue illustrate the morphology of mitochondria. (B) Cardiac NADPH content was measured (n = 5). (C) Mouse hearts were stained with TUNEL (red) and DAPI (blue) in order to detect apoptotic cells (n = 4). TUNEL positive nuclei are quantified. The data is presented as the mean value plus or minus the standard error of the mean. Statistical significance is denoted by *p < 0.05 and **p < 0.01.

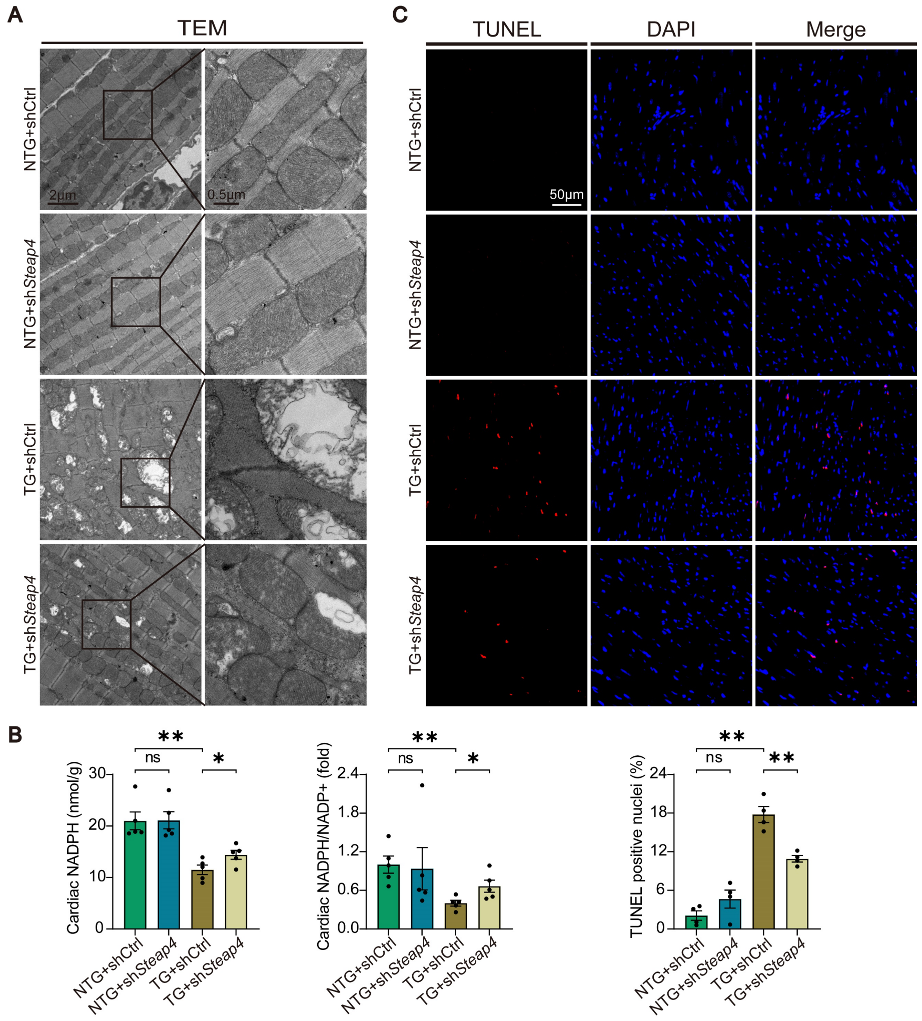
A
TEM
NTG+shCtrl
NTG+shSteap4
TG+shCtrl
TG+shSteap4
2μm
0.5μm
C
TUNEL
DAPI
Merge
50μm
B
Cardiac NADPH (nmol/g)
Cardiac NADPH/NADP+ (fold)
TUNEL positive nuclei (%)
ns
**
*
